# Transcriptional profiling of β-2M^−^SPα-6^+^THY1^+^ spermatogonial stem cells in human spermatogenesis

**DOI:** 10.1016/j.stemcr.2022.02.017

**Published:** 2022-03-24

**Authors:** Maelle Givelet, Virginie Firlej, Bruno Lassalle, Anne Sophie Gille, Clementine Lapoujade, Isabelle Holtzman, Amandine Jarysta, Farahd Haghighirad, Florent Dumont, Sébastien Jacques, Franck Letourneur, Françoise Pflumio, Isabelle Allemand, Catherine Patrat, Nicolas Thiounn, Jean Philippe Wolf, Lydia Riou, Virginie Barraud-Lange, Pierre Fouchet

**Affiliations:** 1 Université de Paris and Université Paris-Saclay, CEA, UMR Stabilité Génétique Cellules Souches et Radiations, iRCM/IBFJ, Laboratoire des Cellules Souches Germinales, 92265 Fontenay-aux-Roses, France; 2 Institut Cochin, INSERM U1016, Département de Génétique, Développement et Cancer, Équipe Génomique Epigénétique et Physiopathologie de la Reproduction, 75014 Paris, France; 3 UFR Médecine Paris Centre-Université de Paris, 15 rue de l’école de Médecine, 75006 Paris, France; 4 Assistance Publique-Hôpitaux de Paris, Hôpitaux Universitaires Paris Centre, CHU Cochin, Histologie-Embryologie-Biologie de la Reproduction, 75014 Paris, France; 5 Université Paris Saclay, UMS IPSIT, 92296 Châtenay-Malabry, France; 6 Université de Paris, Institut Cochin, INSERM, U1016, CNRS UMR8104, Plateforme Séquençage et Génomique, 75014 Paris, France; 7 Université de Paris and Université Paris-Saclay, INSERM, CEA, UMR Stabilité Génétique Cellules Souches et Radiations, iRCM/IBFJ, LSHL, 92265 Fontenay-aux-Roses, France; 8 Department of urology and transplant surgery, Hôpital européen Georges-Pompidou, AP-HP, Université de Paris, 20 rue Leblanc, 75015 Paris, France

**Keywords:** human, spermatogenesis, stem cell, transcriptome

## Abstract

Male infertility is responsible for approximately half of all cases of reproductive issues. Spermatogenesis originates in a small pool of spermatogonial stem cells (SSCs), which are of interest for therapy of infertility but remain not well defined in humans. Using multiparametric analysis of the side population (SP) phenotype and the α-6 integrin, THY1, and β-2 microglobulin cell markers, we identified a population of human primitive undifferentiated spermatogonia with the phenotype β-2 microglobulin (β-2M)^−^SPα-6^+^THY1^+^, which is highly enriched in stem cells. By analyzing the expression signatures of this SSC-enriched population along with other germinal progenitors, we established an exhaustive transcriptome of human spermatogenesis. Transcriptome profiling of the human β-2M^−^SPα-6^+^THY1^+^ population and comparison with the profile of mouse undifferentiated spermatogonia provide insights into the molecular networks and key transcriptional regulators regulating human SSCs, including the basic-helix-loop-helix (bHLH) transcriptional repressor HES1*,* which we show to be implicated in maintenance of SSCs *in vitro.*

## Highlights

• Human β-2M^−^SPα-6^+^THY1^+^ undifferentiated spermatogonia are enriched in stem cells
• Comparative transcriptomics analysis of human and murine spermatogonia
• HES1 is involved in the physiology of SSCs *in vitro*

## Teaser

Givelet et al. identified a population of human primitive undifferentiated spermatogonia, highly enriched in stem cells, with the phenotype β-2M^−^SPα-6^+^THY1^+^. They established transcriptome profiling of this primitive undifferentiated spermatogonium along with other germinal progenitors, providing an exhaustive transcriptome of human spermatogenesis.

## Introduction

Throughout a male’s reproductive life, the pool of spermatogonial stem cells (SSCs) can self-renew or differentiate before undergoing meiosis and then spermiogenesis to produce sperm. SSCs constitute a potential source of cells that can be used to develop regenerative medicines for infertility, especially after cancer treatments during childhood. Testicular transplantation of SSCs has been found to lead to efficient production of functional sperm and restoration of fertility in several animal models (Brinster, 2007), including non-human primates (Hermann et al., 2012).

In mice, the best-characterized mammal model, SSCs are a subpopulation of A_single_ (A_s_) spermatogonia in the adult testis (de Rooij, 2017). These stem cells can self-renew or differentiate into committed paired (A_p_) and aligned (A_al_) spermatogonia, which are collectively called undifferentiated spermatogonia. However, this hierarchical model has been questioned because of recent evidence showing some equipotency in terms of regenerative potential among GFRA1^+^ undifferentiated spermatogonia, with stemness apparently not restricted to only A_s_ spermatogonia (Hara et al., 2014). Compared with mice, identification of the SSC pool and the molecular mechanisms governing their self-renewal and differentiation remains largely elusive in humans. The prevailing model holds that human spermatogenesis arises from A_dark_ and A_pale_ spermatogonia, which are thought to represent reserve and active stem cells, respectively (Ehmcke and Schlatt, 2006). Recent single-cell RNA sequencing (scRNA-seq) studies greatly contributed to highlight the heterogeneity of the premeiotic spermatogonial population and helped to define different cell states in the population of primitive spermatogonia (Wang et al., 2018; Guo et al., 2018; Hermann et al., 2018; Sohni et al., 2019). As illustrated by the previous studies describing several cell surface proteins enabling selection of populations of human spermatogonia with repopulation potential after testicular transplantation (Zohni et al., 2012; Dovey et al., 2013; Nickkholgh et al., 2014; Valli et al., 2014; Tan et al., 2020; Shami et al., 2020), reliable phenotypic markers that allow purification and study of populations of stem cells and progenitors are of significant importance to understand the self-renewal of human SSCs.

Here we developed a fluorescence-activated cell sorting (FACS)-based method that enabled us to purify a population of primitive undifferentiated spermatogonia, highly enriched in SSCs, and five premeiotic, meiotic, and postmeiotic populations of human germ cells. We established an exhaustive transcriptome of human spermatogenesis and examined the dynamics of gene expression throughout the developmental process. This study provides insights into the molecular networks potentially implicated in regulation of primitive spermatogonia and SSCs in humans.

## Results

### Characterization of adult human spermatogenesis using β-2 microglobulin, side population, α-6 integrin, THY1, and DNA content markers

**Figure 1:**
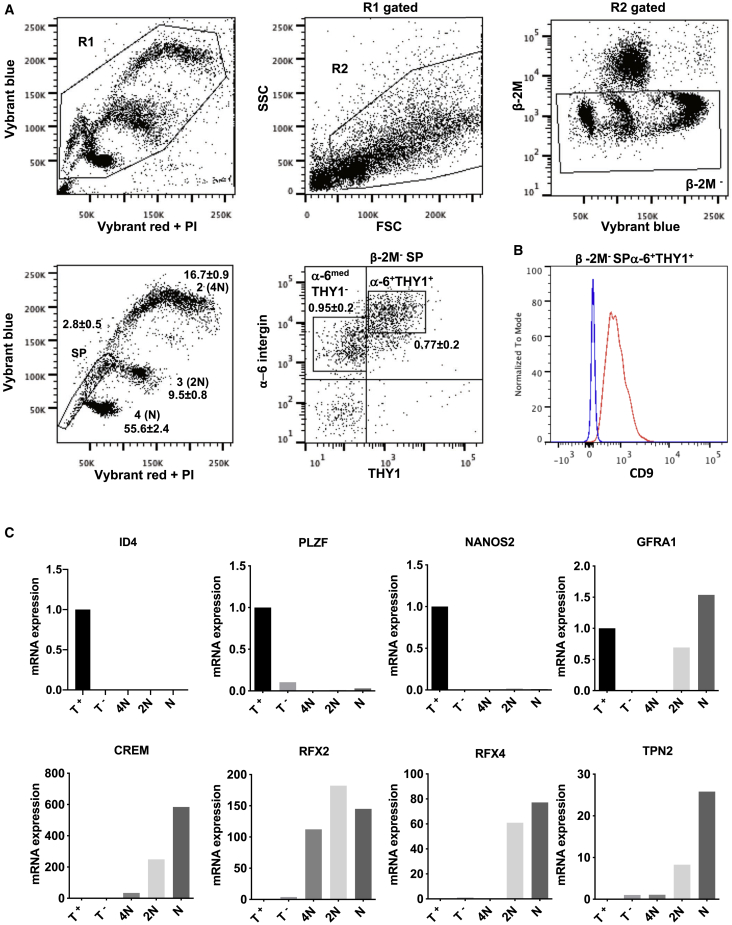
Characterization of the different steps of human spermatogenesis (A) Characterization of the different steps of human spermatogenesis by flow cytometry according to the forward scatter (FSC), side scatter (SSC), blue and red Vybrant fluorescence, β-2M, α-6 integrin, and THY1 parameters as well as side population (SP) and meiotic and postmeiotic subpopulations 2 (4N), 3 (2N), and 4 (N). The frequency (percentage) of the subpopulations in the whole β-2M^−^ population is indicated (mean ± SEM, n = 5). (B) CD9 expression in the β-2M^−^ SPα-6^+^ THY1^+^ population (red line). The control (blue line) corresponds to the signal in CD9^−^/low-expressing postmeiotic round spermatids. (C) Analysis by qRT-PCR of the expression of different spermatogonial (*ID4*, *NANOS2*, *PLZF*, and *GFRA1*) and meiotic (*CREM*, *RFX2*, *RFX4*, and *TNP2*) markers in meiotic and postmeiotic subpopulations 2 (4N), 3 (2N), and 4 (N) and the spermatogonial β-2M^−^SPα-6^+^THY1^+^ (T^+^) and β-2M^−^SPα-6^med^THY1^−^ (T^−^) populations

We and others have shown previously that, in mice, SSCs and spermatogonial progenitors express α-6 integrin and Thy-1 and harbor the side population (SP) phenotype according to an analysis of vital DNA dye efflux by the (ATP Binding Cassette) ABC transporter *Bcrp1/Abcg2* (Lassalle et al., 2004; Falciatori et al., 2004; Barroca et al., 2009). Assuming that mouse and human models of SSCs and spermatogonial progenitors share some characteristics, we applied this multi-parameter flow cytometry strategy (Figure 1A) to characterize human spermatogenesis using this set of markers and the pan-somatic marker β-2 microglobulin (Figure S1A). Viable cells (gate R1) were analyzed using forward and side scatter to remove elongated spermatids and sperm from further analyses (gate R2). After gating for β-2 microglobulin (β-2M)^−^ cells to remove somatic cells, we were able to resolve the complex Vybrant red and blue fluorescently labeled cells into five major cell populations, including an SP containing cells that actively excluded the DNA dye. Subpopulations 2 (4N DNA content), 3 (2N DNA content), and 4 (N DNA content) were assumed to correspond to spermatocyte I, spermatocyte II, and haploid (N) spermatids cells, respectively. To further this characterization, the SP population was divided based on α-6 integrin and THY1 expression. β-2M^−^α-6^+^THY-1^+^ cells and β-2M^−^α-6^med^THY1^−^ cells represented, respectively, 0.77% ± 0.2% and 0.95% ± 0.2% of the β-2M^−^ cell population (n = 5). Using the opposite gating scheme, we confirmed that β-2M^−^α-6^+^THY1^+^ cells were found mainly in the SP population and represented 69.7% ± 2.7% (n = 11) of the SP cells (Figures S1B–S1H). β-2M^−^SPα-6^+^THY1^+^ and β-2M^−^ SP α-6^med^ THY1^−^ cells also expressed the human spermatogonial marker CD9 (Figures 1B and S1I). In addition to expression of spermatogonial markers *ID4*, *NANOS2*, *PLZF*, and *GFRA1* (Figure 1C), we found that *PIWIL4*, *C19orf84*, *TSPAN33*, *PLPPR3*, *FGFR3*, and *UTF1* primitive spermatogonial markers were expressed in the β-2M-SPα-6^+^THY1^+^ population, whereas *KIT* and *STRA8* differentiating markers were expressed in the β-2M-SPα-6^med^THY1^−^ population (Figure S2A). The vimentin somatic marker was not detected in β-2M^−^SPα-6^+^THY1^+^ cells, showing that the β-2M^−^SPα-6^+^THY1^+^ population is not composed of somatic cells (Figure S2B). On the other hand, vimentin was highly expressed in the β-2M^+^ cell population (Figures S1B and S2B), and the pan-germinal *VASA* (*DDX4*) marker and *FGFR3* and *UTF1* undifferentiated spermatogonial markers were detected at very low levels, the β-2M^+^ population being composed of somatic cells. The lower expression level of α-6 integrin observed in the β-2M^−^SP α-6^med^THY1^−^ fraction, in addition to the lack of expression of markers of primitive spermatogonia and meiotic cells (Figures 1C and S2A), indicated that these cells were in a more advanced differentiation stage and represented a differentiating spermatogonial population. The expression levels of meiotic markers (*CREM*, *RFX2*, *RFX4*, and *TNP2*) suggested that subpopulations 2 (4N), 3 (2N), and 4 (N) corresponded to the spermatocyte I, spermatocyte II, and spermatid populations, respectively. These data show that the β-2M^−^SPα-6+THY1^+^ population contains primitive spermatogonia and lacks somatic cells.

### The human spermatogonial SP phenotype depends on ABC transporter activity of the *BCRP1/ABCG2* gene

**Figure 2:**
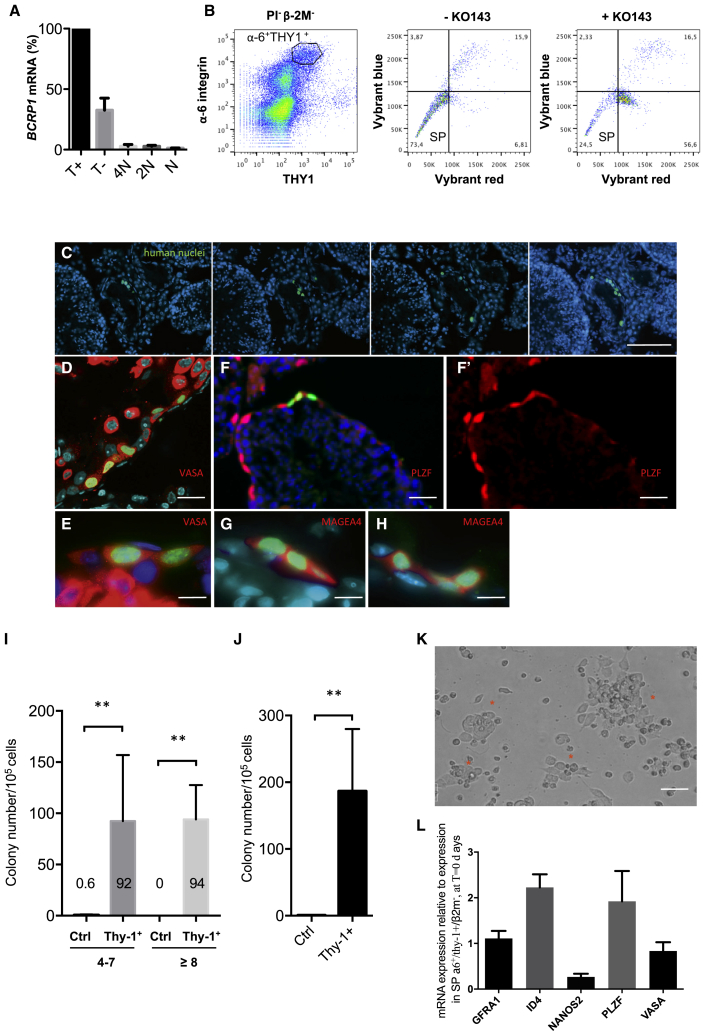
The SP phenotype is BCRP1/ABCG2 dependent, and the β-2M^−^SPα-6^+^THY1^+^ (T^+^) population possesses SSC potential (A) The BCRP1/ABCG2 gene is expressed in the T^+^ subpopulation. Analysis by qRT-PCR of the expression of *BCRP1* in T^+^, T^−^, spermatocyte I (4N), spermatocyte II (2N), and spermatid (N) populations (n = 3 experiments). (B) Ko-143 inhibits the SP phenotype in the T^+^ subpopulation. The frequencies of the populations are indicated. (C) Serial sections of testes obtained from NSG mice 2 months after transplantation with T^+^ cells. Human cells (green, antibody specific to human nuclei) observed at the murine basement membrane on successive sections indicate human colonizing cell clusters. (D and E) VASA (red) expression in human cells (green nuclei) from colonizing cell clusters. (F and F’) PLZF expression in human cells (green, human nuclei; red, PLZF) from colonizing cell clusters. (G and H) MAGE-4 expression in human cells (green, human nuclei; red, MAGEA-4 Blue indicates 4′,6-diamidino-2-phenylindole (DAPI) from colonizing cell clusters. (I) Comparison of the colonization efficiency of recipient testes transplanted with T^+^ cells or control β-2M^−^not(SPα-6^+^ THY1^+^) cells. Human colonizing cell clusters were separated in two groups according to their size: one group composed of cluster of 4–7 cells and the other of cluster of 8 or more cells (control, n = 4 recipient testes; THY1^+^, n = 5 recipient testes; transplantations from five human donors). (J) The total number of cell clusters generated per 10^5^ cells injected (control, n = 4 recipient testes; THY1^+^, n = 5 recipient testes; transplantations from five human donors). (K) Cell clusters observed *in vitro* after 14 days of culture starting with the T^+^ cell population. (L) Expression of markers of immature spermatogonia in cell clusters after 15 days of culture (n = 6 replicates from 2 independent cultures). Scale bars: 50 μm (C), 20 μm (D), 40 μm (F, F’, and K), and 10 μm (E, G, and H).

The SP phenotype is caused by active efflux of DNA dye via members of the ABC transporter superfamily. Specifically, *BCRP1/ABCG2*, an ABC transporter, has been shown to play a major role in development of the SP phenotype in stem and progenitor cells during murine hematopoiesis and spermatogenesis and in human cancer stem-like cells (Zhou et al., 2001; Lassalle et al., 2004; Bleau et al., 2009). We found that the *BCRP1/ABCG2* gene was expressed in the β-2M^−^SPα-6^+^THY1^+^ population of primitive spermatogonia (Figure 2A). In the presence of the specific BCRP1 inhibitor Ko143 (Allen et al., 2002), Vybrant efflux was markedly reduced in the β-2M^−^SPα-6^+^ THY1^+^ population (Figure 2B) and resulted in a reduction of the SP population by 68.5% ± 2%. These results suggest that BCRP1 transporter activity is involved in the human germinal SP phenotype.

### In adults, the β-2M^−^SPα-6^+^THY1^+^ population of primitive spermatogonia is highly enriched in human SSCs

Transplantation assays are a critical tool to test the capacity of putative SSCs to regenerate spermatogenesis after transplantation into germ cell-depleted testes. When transplanted, human SSCs show only a limited capacity to proliferate and differentiate in the testes of immunodeficient mice. However, these experiments allow evaluation of the potential of transplanted putative SSCs to migrate, survive, and colonize and are currently the gold-standard test for human SSCs (Dovey et al., 2013; Valli et al., 2014). β-2M^−^SPα-6^+^THY1^+^ cells obtained from human testicular biopsies were sorted and transplanted (3,700–9,600 cells per testis) into busulfan-depleted testes of *NOD*^*−*^*Scid/IL2Rγc*^*−/−*^ (NSG) humanized mice. As a control, a population including all germinal cells excluding SPα-6^+^ THY1^+^cells (i.e., all β-2M^−^ cells excluding SPα-6^+^ THY1^+^ cells) was sorted and transplanted (39,600–83,000 cells per testis). The rate of colonization by human SSCs was estimated via immunofluorescence analysis using an antibody against human DNA nuclear protein in serial sections of recipient testes (Bissig-Choisat et al., 2015). Clusters of human cells residing close to the basal membrane were observed 2 months after transplantation (Figure 2C). The germinal markers VASA (Figures 2D and 2E) and MAGEA4 (Figures 2G and 2H) were expressed in all cells from the human cluster, and 78% ± 10% of the cells in these clusters (n = 8 clusters) were positive for PLZF (Figures 2F and 2F’), indicating that the cells derived from human β-2M^−^SPα-6^+^THY1^+^ donor cells were mainly spermatogonia (Eildermann et al., 2012). The clusters were counted and separated into two classes according to the number of cells per cluster (4–7 cells and ≥8 cells). These data show that human SSCs, having the capacity to colonize murine recipient testes, are found in the β-2M^−^SPα-6^+^ THY1^+^ population and that germinal populations that do not have this phenotype (all β-2M^−^ cells except SPα-6^+^ THY1^+^ cells) are devoid of SSCs (Figure 2I). Hence, the β-2M^−^SPα-6^+^THY1^+^ population is highly enriched in human SSCs. The stem cell activity of this population (187 colonies/10^5^ donor cells; Figure 2J) appeared to fall within the same range but was roughly 4-fold higher than what has been reported in previously published studies, approximately 50 colonies/10^5^ donor cells when using EPCAM or α6-integrin as markers (Dovey et al., 2013; Valli et al., 2014). Human SSCs can be cultured *in vitro* for 2–3 weeks (Medrano et al., 2016). Germinal cluster formation was observed clearly only in wells seeded with β-2M^−^SPα-6^+^ THY1^+^ cells in the presence of the growth factors glial cell line-derived neurotrophic factor (GDNF) and fibroblast growth factor 2 (FGF2) up to 2 weeks of culture (Figure 2K). Cell clusters continued to express markers of immature spermatogonia after 15 days of culture (Figure 2L). These data show that the β-2M^−^SPα-6^+^ THY1^+^ population is highly enriched in SSCs.

### The transcriptome of human spermatogenesis reveals a specific profile of expression for each differentiation stage

We took advantage of the FACS-based method of purification of human germinal cells to examine the transcriptomic activity of the differentiation stages of human spermatogenesis. The β-2M^−^SPα-6^+^THY1^+^ and β-2M^−^SPα-6^med^THY1^−^ spermatogonial populations were sorted along with an additional subpopulation referred to here as β-2M^−^α-6^med^THY1^−^-“S-phase,” with a Vybrant profile exhibiting a continuum between 2N and 4N in DNA content (Figure S1E). We assumed that this population would contain actively replicating S-phase cells destined to differentiate into spermatocyte I cells and should therefore correspond to late-differentiating spermatogonia. The meiotic spermatocyte I and II populations and round spermatids were also purified. A somatic RNA reference sample was prepared from a mixture of CD45^+^ blood cell RNA, prepuce tissue RNA, and esophageal tissue RNA.

**Figure 3:**
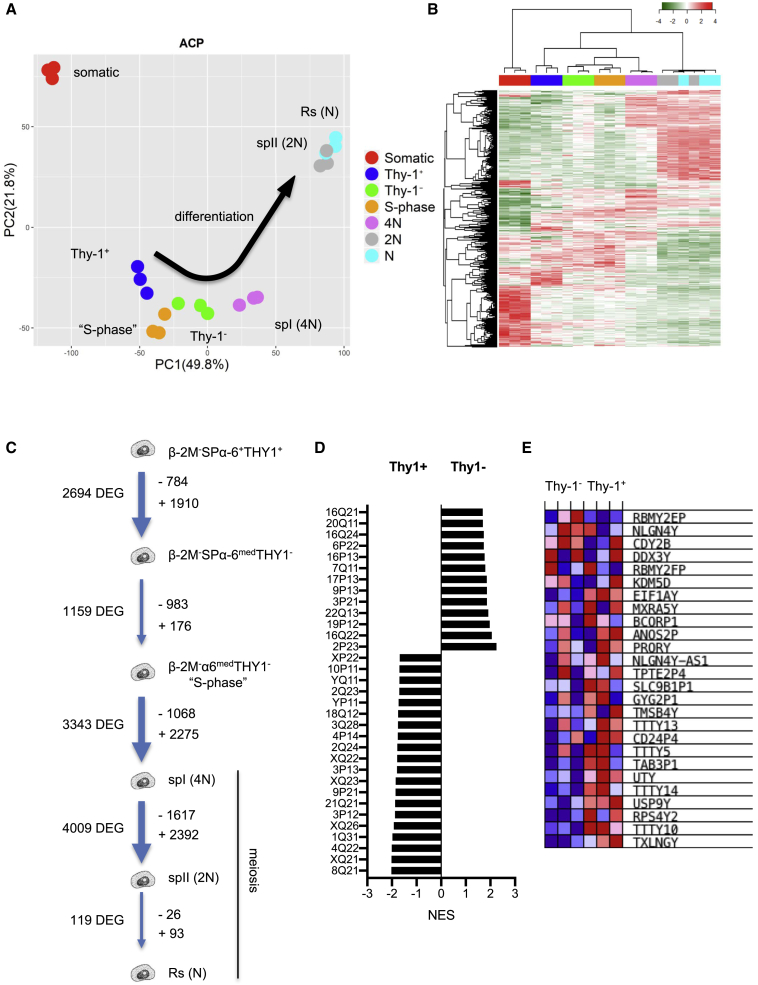
Differential expression of genes during human spermatogenesis (A) PCA of differentially expressed genes in human germinal populations throughout spermatogenesis and control somatic tissue (PC1–PC2 contributed to intersample variation, as shown in parentheses). (B) Heatmap of the gene expression of the differentiation stages during spermatogenesis and associated hierarchical clustering. (C) Schematic recapitulating the number of differentially expressed genes (DEGs) at transitions between the different differentiation stages. (D) GSEA of the expression profile signature at the transition of β-2M^−^SPα-6^+^THY1^+^immature spermatogonia to more differentiated spermatogonia according to the chromosomal position of the genes (FDR < 0.03). (E) Heatmap of DEGs found in the Yq11 region.

Of the 29,597 gene probes tested, paired comparisons of these populations identified 10,161 genes differentially expressed during the differentiation process (p < 5 × 10^−4^). A principal-component analysis (PCA) of these genes revealed a clear trend for the transcriptomes to diverge along the germinal differentiation process (Figure 3A). Premeiotic spermatogonial populations clustered together, whereas meiotic and postmeiotic populations diverged, and the somatic transcriptome was clearly distinct from the germinal expression. A heatmap analysis and associated hierarchical clustering of the sorted populations confirmed the sequence of steps during differentiation from the most primitive spermatogonia to haploid spermatids (Figure 3B). Strikingly, the spermatocyte II and round spermatid clusters overlapped, indicating that they have similar gene expression profiles and that developmental determination of haploid cells is initiated at the spermatocyte II stage (Figures 3A and 3B). The dynamics of the germinal transcriptome reveal that transitions between adjacent steps of differentiation were associated with dramatic changes in gene expression during adult human spermatogenesis (Figure 3C).

The transcription status of the sexual chromosomes plays an important role during male germ cell development, especially at meiosis, because of the epigenetic inactivation of meiotic sex chromosomes (MSCI) (Turner, 2007). We observed a dramatic decline of expression of X- and Y-linked genes, as expected, at the cell transition to the spermatocyte I stage (spermatocyte I/β-2M^−^α-6^med^THY1^−^-“S-phase” comparison). The contribution of sexual chromosomes to the transcriptional program decreased, respectively, from 15.4% (165 X-linked genes/1,068 genes) to 0.92% of the transcriptome (21 X-linked genes/2,275 genes) and 0.65% (7 Y-linked genes/1,068 genes) to 0% of the transcriptome (0 Y-linked genes/2,276 genes) for X and Y chromosomes (Table S1; p < 0.02, fold change-fc > 2). During transition from spermatocyte I to spermatocyte II, some X- and Y-linked genes continue to be downregulated (64 X-linked genes, 3 X/Y-linked genes, and 1 Y-linked gene), but transcription of other X and Y chromosome genes appeared to be reactivated as soon as the cells reached the spermatocyte II stage (92 X-linked, 3 XY-linked, and 5 Y-linked genes over 2,392 genes upregulated in spermatocyte II), suggesting that X and Y chromosomes were transcriptionally active in postmeiotic spermatids, as shown recently in mice (Margolin et al., 2014).

X-linked genes have also been suggested to be expressed preferentially in premeiotic spermatogonia in spermatogenesis (Wang et al., 2001). Higher numbers of X- and Y-linked genes were expressed in the β-2M^−^SPα-6^+^THY1^+^ transcriptome compared with β-2M^−^SPα-6^med^THY1^−^ at the transition of immature spermatogonia to differentiated spermatogonia: genes in Xq21, Xq22, Xp22, Xq23, Xq26, Yp11, and Yq11 chromosomal regions (Figures 3D and 3E). Interestingly, the azoospermia factor (AZF) locus is located in the Yq11 region, which contains three regions, AZFa (*UTY* and *USP9Y* genes), AZFb (*RPS4Y2* and *TTTY14*, *TTTY5*, *TTTY13*, and *TTTY10* genes), and AZFc, in which microdeletions are associated with fertility issues (Vogt et al., 2017).

### Expression profiles of primitive spermatogonial populations enriched in SSCs

**Figure 4:**
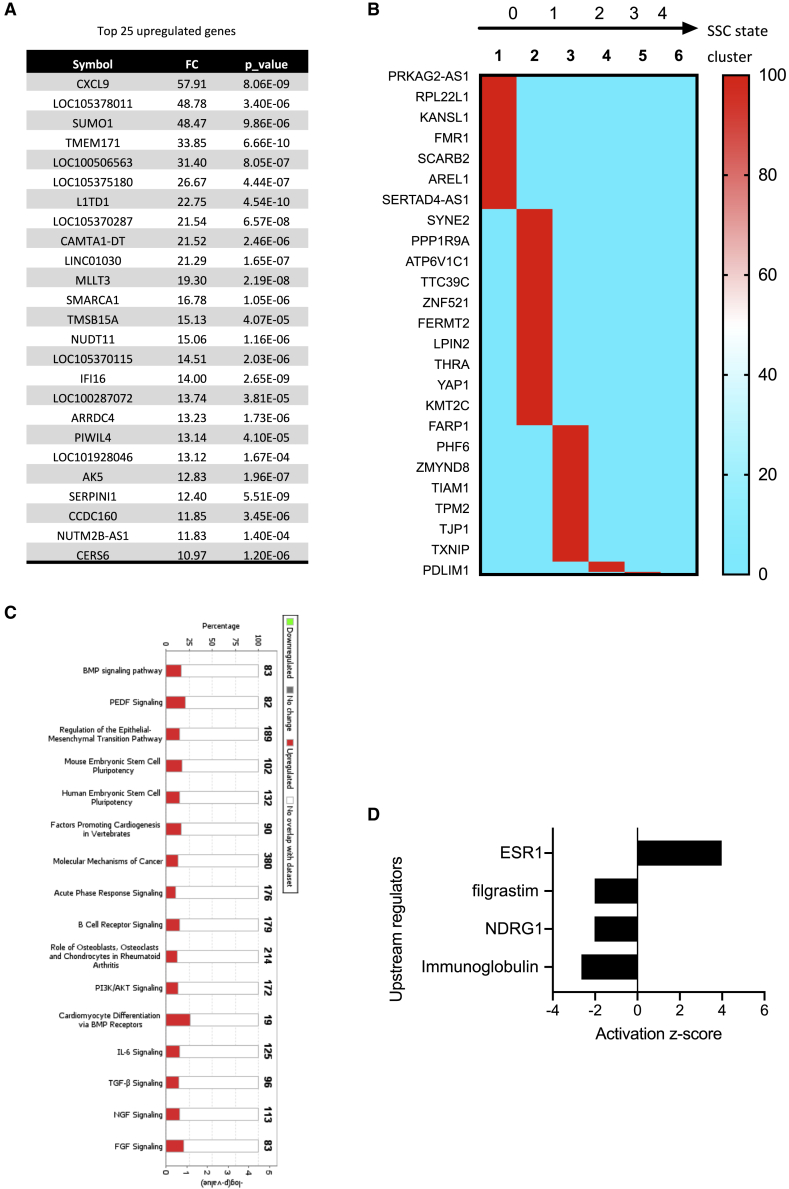
Expression signature of the primitive spermatogonial T^+^ population enriched in SSCs (A) List of the top 25 DEGs. (B) Heatmap showing the distribution of the expression of genes of the T^+^ population according to the six gene clusters defining the states 0–4 in human adult SSC development as defined by Guo et al. (2018). (C) List of the top canonical pathways identified via an Ingenuity Pathway Analysis (IPA). (D) Putative upstream regulators identified using IPA.

Gene lists were extracted from paired comparisons of cells in adjacent stages of differentiation to display the transcriptomic signatures of the different steps of spermatogenesis. We focused our analysis on the β-2M^−^SPα-6^+^THY1^+^ population of immature spermatogonia that contains SSCs. The transcriptomics profiles of populations of differentiating spermatogonia, meiotic and postmeiotic populations, are provided in Figures S3–S5 and Table S2. We identified 784 genes (fc > 2, p < 0.02) with higher expression in the β-2M^−^SPα-6^+^THY1^+^ population than in the downstream progeny, the β-2M^−^SPα-6^med^THY1^–^ population (Table S2). Several of these genes have been reported previously to be relevant in murine SSCs and spermatogonial progenitors. These include *PIWIL4*, *FGFR2*, *GFRA1*, *ADGRA3/GPR125*, *PIK3CA*, *SALL1*, *SALL3*, *TEC*, *ZBTB33*, and *ABCG2/BCRP1.* The top 25 genes are listed in Figure 4A, and the expression of some of these was confirmed by qRT-PCR (Figure S6A). Using an scRNA-seq approach, Guo et al. (2018) identified six gene clusters defining states 0–4 in human adult SSC development. When the β-2M^−^SPα-6^+^THY1^+^ gene list was compared with genes expressed in these six clusters, we found 194 genes common to both lists, of which 97.4% (189 of 194) were distributed in clusters 1, 2, and 3, corresponding to states 0 and 1 in SSC development (Figure 4B). Similar results were obtained when the β-2M^−^SPα-6^+^THY1^+^ gene list was compared with another single-cell transcriptomic analysis of human SSCs (Sohni et al., 2019); 113 common genes were found, of which 71.7% (81 of 113) were distributed in clusters 1A, 1B, 1C, and 2 describing the SSC population (Figure S6B). Compared with the transcriptome of the SSC highly enriched PLPPR3^+^ population (Tan et al., 2020), we observed that 29% of genes (228 of 784) of the β-2M^−^ SPα-6^+^THY1^+^ population was shared with the PLPPR3^+^ population, whereas only 11.3% (216 of 1,910) of the transcriptome of PLPPR3^+^ cells was found in the β-2M-SPα-6^med^THY1^−^ population (Figure S6C). This suggested that the β-2M^−^SPα-6^+^THY1^+^ population contained the primitive states of SSC development.

Molecular function and cellular component annotations of the β-2M^−^SPα-6^+^ THY1^+^ gene list according to PANTHER Gene Ontology classification showed that the top biological processes are involved in control of cell movement (motility and cell migration) and in transcriptional regulation of gene expression (Tables S3 and S4). Using Ingenuity Pathway Analysis (IPA), we found that the bone morphogenetic protein pathway, pigment epithelium-derived factor (PEDF) pathway, epithelial-mesenchymal transition (EMT) pathway, and embryonic stem cell pluripotency pathway were among the most highly regulated canonical pathways in the β-2M^−^SPα-6^+^THY1^+^ population (–log p > 3; Figure 4C). In addition, nuclear hormone receptor estrogen receptor ESR1, N-myc downstream-regulated gene 1 (NDRG1), and granulocyte-colony-stimulating factor (G-CSF; filgrastim) signaling were predicted to be potential top upstream regulators of the physiology of primitive spermatogonia (Figure 4D).

**Figure 5:**
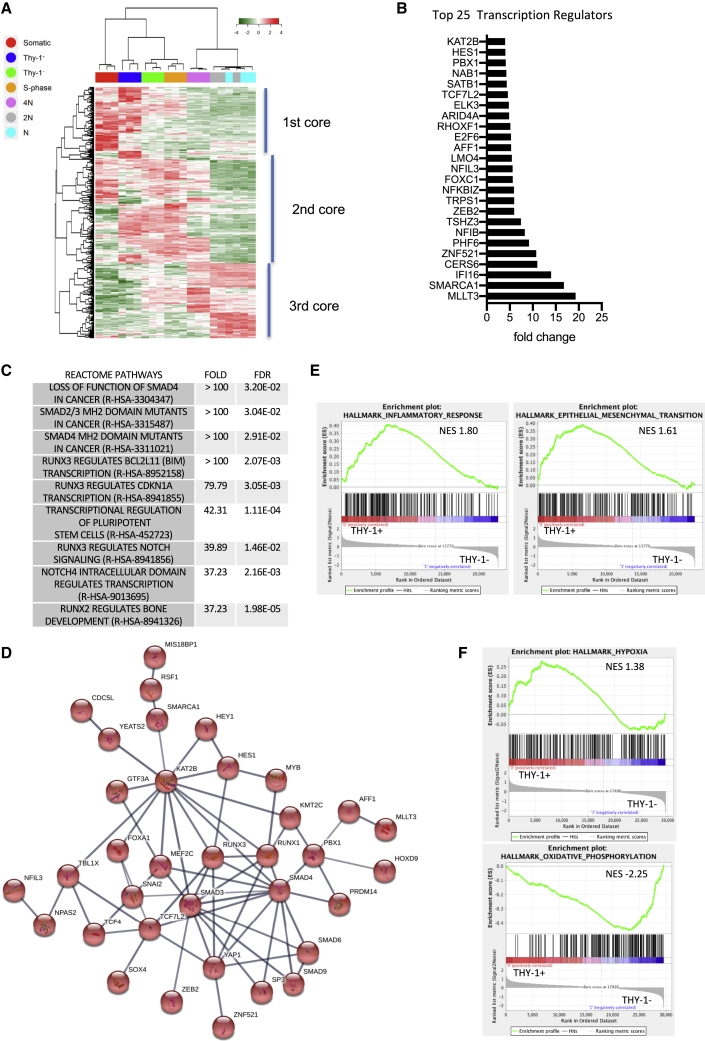
Identification of TRs enriched in the primitive spermatogonial T^+^ population (A) Heatmap of TRs expressed during spermatogenesis and associated hierarchical clustering. (B) List of the top 25 differentially expressed TRs in the T^+^ population. (C) PANTHER enrichment analysis of the T^+^ set of TRs. (D) Network of 35 TRs identified using String analysis. (E and F) GSEA of the cell state transition between the T^+^ and T^−^ spermatogonial populations

Next we focused on transcriptional regulators (TRs) because they play crucial roles in differentiation. Among the 492 TRs that varied across the stages of spermatogenesis (fc > 2, p < 0.02; Table S2), we observed 3 core groups (Figure 5A). The first core was expressed in immature spermatogonia and partially overlapped with the set of TRs expressed in somatic tissues and differentiating spermatogonia. The second core seemed to be activated when spermatogonia became committed to differentiate up to meiosis. The third core began to activate at the onset of meiosis and was fully expressed in later stages. We identified 109 TRs that were preferentially enriched in the β-2M^−^SPα-6^+^THY1^+^ population of immature spermatogonia (Figure 5B; Table S2), including *TAF4B*, *SALL1*, *PRDM1*, and *PRDM14*, known to play a role in germinal lineage development. Enrichment analysis using PANTHER highlighted a role of RUNX and transforming growth factor (TGF)/bone morphogenetic protein (BMP)/SMAD pathways (Figure 5C), which are involved in stem cell biology (Mevel et al., 2019; Mullen and Wrana, 2017). Interestingly, String analysis identified a network of interactions between 35 of these TRs involved in the regulation of the β-2M^−^SPα-6^+^THY1^+^ population (Figure 5D).

We analyzed the cell state transition between the β-2M^−^ SPα-6^+^THY1^+^ and β-2M^−^ SPα-6^med^ THY1^−^ populations by performing gene set enrichment analysis (GSEA). The genes involved in the inflammatory transcriptional program were enriched in β-2M^−^ SPα-6^+^THY1^+^ cells (Table S5; Figure 5E; false discovery rate [FDR] < 0.1). Of note, GSEA also suggests also a role for RAS, transforming growth factor β (TGF-β), Hedgehog, Notch, and the EMT pathway in regulation of differentiation of primitive spermatogonia. Transition from the β-2M^−^ SPα-6^+^THY1^+^ state to the β-2M^−^ SPα-6^med^THY1^−^ state seems to be elicited by genes involved in the MYC, E2F, or unfolded protein response pathway (Table S6). This cell state transition appears to be supported by a switch from hypoxic to oxidative phosphorylation metabolism (Figure 5F).

### Comparative transcriptomics analysis of populations of primitive spermatogonia in humans and mice

**Figure 6:**
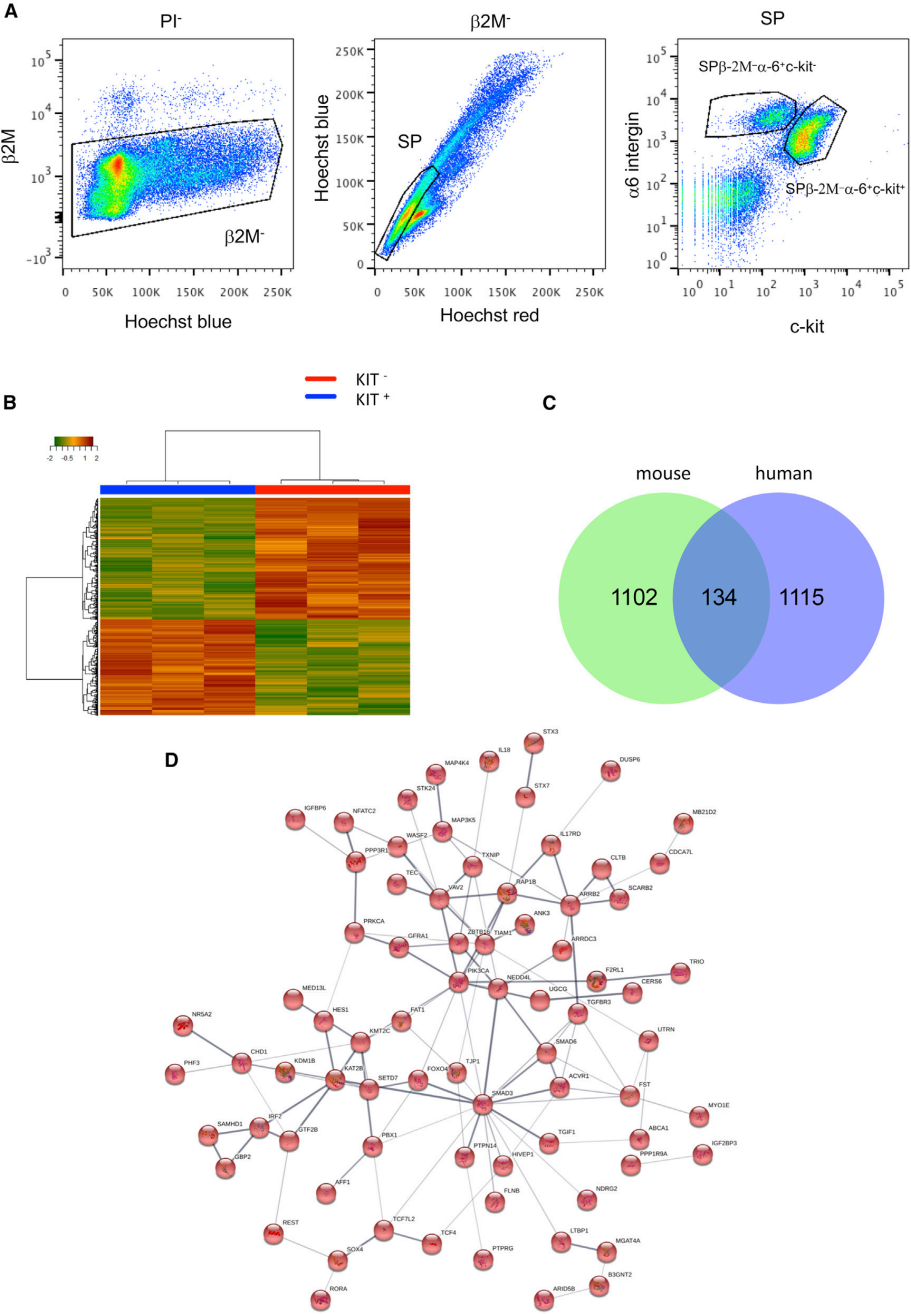
Comparative analysis of DEGs in human and mouse populations of adult primitive spermatogonia (A) Flow cytometry analysis of mouse α-6^+^ testicular cells selected by magnetic activated cell sorting (MACS) based on blue and red Hoechst fluorescence and the markers β2M, α-6 integrin, and c-kit. The SPβ2M^−^α-6^+^c-kit^−^ and SPβ2M^−^α-6^+^c-kit^+^ populations are indicated. Cells were also selected on FSC parameters to exclude elongated spermatids. (B) Heatmap of the gene expression in the mouse undifferentiated SPβ-2M^−^α-6^+^c-kit^−^ and differentiated SPβ-2M^−^α-6^+^c-kit^+^ spermatogonial population and associated hierarchical clustering. (C) Venn diagram showing the relationships between the transcriptomes of human T^+^ and mouse β-2M^−^SPα-6^+^c-kit^−^ spermatogonial populations. The number of genes in each group is indicated. (D) Network of 76 interacting genes using String analysis in the conserved set of genes between mouse and human models.

As shown previously (Barroca et al., 2009; Corbineau et al., 2017), spermatogonial progenitors were discriminated by flow cytometry in mice as follows: β-2M^−^SPα-6^+^c-kit^−^ cells correspond to undifferentiated spermatogonia, including SSCs, and β-2M^−^SPα-6^+^c-kit^+^ cells correspond to differentiating spermatogonia (Figure 6A). Transcriptomics analysis was performed on sorted populations of β-2M^−^SPα-6^+^c-kit^−^ and β-2M^−^SPα-6^+^c-kit^+^ spermatogonia (Figure 6B). 1,238 and 913 genes, respectively, were differentially expressed between these two populations (fc > 1.5, p < 0.02; Table S7). Genes reported previously to be relevant for undifferentiated spermatogonia were expressed positively in the transcriptomics profile of the β-2M^−^SPα-6^+^c-kit^−^ population, including *Ret*, *Gfra1*, *Etv5*, *Nanos2*, *Nanos3*, *Glis3*, *Sall1*, *Sall4*, *Cdh1*, *Bcl6b*, *Bnc2*, *CD24a*, *Ddit4*, *Ngn3*, *Tec*, *zbtb16*, *Eomes*, *T*, *Nefm*, *Lhx1*, *Smad6*, *TcL1*, *Id4*, *CD9*, *Tspan8*, *Itga6*, *PiwiL4*, *Utf1*, and *Stat3*. We compared the lists of genes enriched in the most primitive spermatogonial population in humans and mice and found that 134 genes were conserved between the human β-2M^−^SPα6^+^THY1^+^ and murine β-2M^−^SPα-6^+^c-kit^−^cell populations (fc > 1.5, p < 0.02 for both lists; Figure 6C; Table S7), including *Piwil4*, *Gfra1*, *Pik3ca*, *Sall1*, *Txnip*, *Tec*, and *Zbtb16*, implicated in SSCs and spermatogonial progenitors. Some genes, such as *Chd1*, *Foxo4*, *Pbx1*, *Tcf4*, and *Hes1*, were shown to play roles in the physiology of embryonic or adult stem cells. String analysis identified a network of 76 interacting genes in this set (Figure 6D).

### The transcription factor *HES1* plays a role in maintenance of murine SSCs *in vitro*

**Figure 7:**
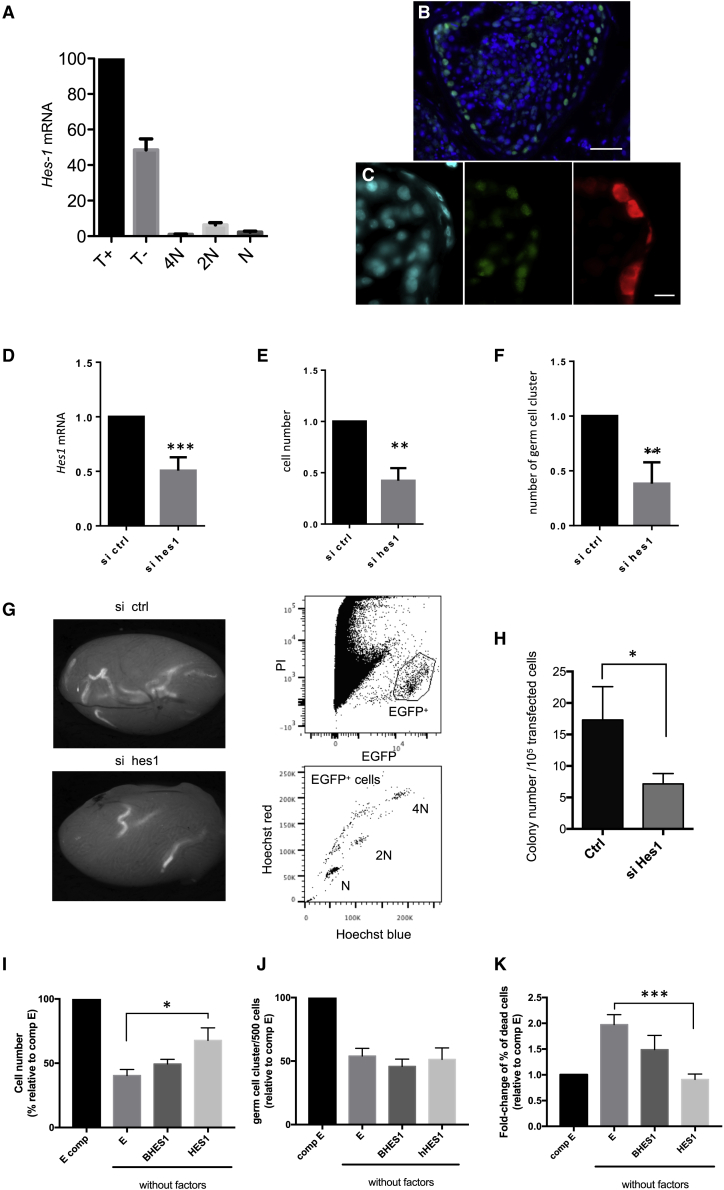
The transcription factor HES1 is involved in maintenance of murine SSCs (A) Analysis by qRT-PCR of the expression of *HES1* in human T^+^, T^−^, spermatocyte I (4N), spermatocyte II (2N), and spermatid (N) populations.(n = 3 experiments). (B and C) Immunofluorescent detection of HES1 in human testes. (B) HES1, green; DAPI, blue; scale bar, 40 μm. (C) HES1, green; MAGE4, red; DAPI, blue; scale bar, 10 μm. (D) *Hes1* silencing in transfected SSCs using siRNA (n = 7 transfection experiments). (E) Analysis of the total number of germ cells (n = 10 colony tests from 7 transfection experiments). (F) Total number of germ cell clusters formed 7 days after transfection (n = 7 transfection experiments). (G) Analysis of regenerative spermatogenesis after transplantation of SSCs 7 days after transfection. Shown is detection of EGFP-fluorescent seminiferous tubules on macroscopic observation in recipient testes 2 months after transplantation in control (top) and *Hes1* siRNA-treated (bottom), EGFP-expressing SSCs. Also shown is red/blue Hoechst 33342 fluorescence analysis of EGFP^+^ cells obtained from a recipient testis transplanted with *Hes1* siRNA-treated, EGFP-expressing SSCs. Meiotic spermatocyte I (2N), spermatocyte II (4N), and postmeiotic (N) cells are indicated. (H) Colonization of recipient testes was lower in testes transplanted with culture of *Hes1* siRNA-treated SSCs than in those transplanted with control siRNA-treated SSCs 7 days after transfection (n = 8 recipient testes from 6 transfection experiments). (I–K) The effects of enforced expression of human HES1 in cultures of SSCs exposed to growth factors and serum deprivation conditions (J) on total cell number (n = 4 experiments), frequency of *in vitro* cluster-initiating cells (n = 12 colony tests from 4 experiments), and (K) cell death (n = 4 experiments). HES1, ΔBHES1 (BHES1), and GFP control (E) cells were grown with growth factors under serum deprivation conditions (without factors) or complete medium (E comp).

To validate the transcriptomics profile of the human β-2M^−^SP^+^α-6^+^THY1^+^ population, we focused on the basic-helix-loop-helix (bHLH) transcriptional repressor HES1, which is involved in development of hematopoietic, neural, and intestinal stem cells (Liu et al., 2015). *Hes1* was also conserved in the transcriptome of murine SPβ-2M^−^α-6^+^c-kit^−^ undifferentiated spermatogonia. We confirmed the highest level of expression of *HES1* mRNA in the β-2M^−^SP^+^α-6^+^THY1^+^ population by qRT-PCR (Figure 7A). The HES1 protein was detected in human cells that contacted the basement membrane (Figure 7B) and particularly in MAGEA4^+^ spermatogonia (Figure 7C). We also observed HES1 in GATA-4^+^ Sertoli cells (Figure S6D). We used culture of murine SSCs, which is the best-characterized mammalian model, to study the role of HES1*. Hes1* was silenced in murine adult SSC culture after transfection with small interfering RNA (siRNA), and SSC functionality was assessed 7 days later, as described previously (Oatley et al., 2010), by *in vitro* colony formation ability tests and transplantation assays (Yeh et al., 2007). *Hes1* mRNA was significantly downregulated 48 h after siRNA transfection (Figure 7D). Seven days after transfection, the total numbers of germ cells (Figure 7E) and of cells able to form germinal cell clusters *in vitro* (Figure 7F) were lower, suggesting alteration of SSC maintenance when *Hes1* expression was reduced. To confirm this effect, EGFP-expressing cells 7 days after transfection were transplanted into the seminiferous tubules of γ-irradiated, germ-cell-depleted testes. Ten weeks after transplantation, *Hes1* siRNA-treated donor cells had colonized the recipient testes and were producing normal spermatogenesis (Figure 7G). However, their colony formation activity was lower than that observed in cells treated with a control siRNA, indicating that the SSC content of the cultures was reduced after *Hes1* silencing (Figure 7H). Because HES1 is involved in regulation of quiescence under serum starvation conditions in several cell types (Sang et al., 2008), we tested the effects of enforced expression of HES1 on SSCs poised for quiescence. Using growth factor and serum deprivation conditions (minimal conditions), we induced quiescence in cultured SSCs, as shown by the increase in Ki-67^−^ cells (Figure S6E). SSCs were transduced with lentiviral vectors expressing human HES1, ΔBHES1, or a control (GFP only) construct, with ΔBHES1 representing an HES1 mutant defective in its DNA-binding function (Yu et al., 2006), and GFP-positive cells were sorted by flow cytometry (Figure S6F). As expected, after 96 h under minimal conditions (i.e., without factors), we observed that there were fewer cells in GFP control cultures than in cultures grown in medium supplemented with serum and growth factors (i.e., complete medium) (Figure 7I). However, the number of cells was higher in HES1 expression-enforced cell cultures than in GFP control cell cultures grown under minimal culture conditions. No effects were observed in cultures of enforced mutant ΔBHES1-expressing cells. The frequencies of the numbers of cells with the ability to form germinal colonies *in vitro* remained unchanged in enforced HES1-expressing cell cultures after 96 h of growth under minimal conditions (Figure 7J). Cell death was observed in SSCs cultured under minimal conditions (Figure 7K), as reported previously in other cell types (Jeffers et al., 2003). Strikingly, we observed that the level of cell death was lower when HES1 expression was enforced and returned to the level observed in control cultures grown in complete medium. Inducing ectopic constitutive expression of human *HES1* in murine SSCs and progenitors seemed to protect the cells against cell death when grown under medium starvation conditions. These data indicate that HES1 plays a role in maintenance of SSCs cultured *in vitro*.

## Discussion

In humans, identification of SSCs is still limited because of a notable lack of markers. Human A_dark_ and A_pale_ spermatogonia show highly similar transcriptomics profiles (Jan et al., 2017), suggesting that morphological nuclear criteria are not necessarily related to progenitor and stem cell states. Different cell states have been delineated in the primitive spermatogonial and SSC population using scRNA-seq (Wang et al., 2018; Guo et al., 2018; Hermann et al., 2018; Sohni et al., 2019; Shami et al., 2020). However, these putative SSC subsets must be validated by functional assays. TSPAN33 and PLPPR3, identified in these subsets, have been confirmed recently as SSC markers using testicular transplantation assays (Tan et al., 2020; Shami et al., 2020). We designed a combination panel of four SSC markers, described previously in mice (Barroca et al., 2009; Corbineau et al., 2017), that allowed us to identify an SSC population highly enriched with the β-2M^−^SPα-6^+^THY1^+^ phenotype, which was validated using transplantation functional assays.

Some of the markers used in our study, such as ITGA6 and THY1, have been reported previously to be human SSC markers, but they were tested individually and not in combination with other markers (Valli et al., 2014). We show that ABCG2 is involved in the DNA dye efflux that results in the human SP phenotype, similar to what has been observed in mice (Lassalle et al., 2004). A relatively high degree of conservation was observed between rodent and primate SSC markers, although the SP phenotype is still under debate, probably because of the different mouse models and experimental procedures and/or different gating used to define it (Kubota et al., 2003). Compared with other mammalian models, a caveat regarding stem cell identification in humans is the issue of lineage tracing analysis, and analysis of the regenerative capacity after testicular transplantation is still the best functional assay to test the stem cell potential. A testicular transplantation assay showed that the β-2M^−^SPα-6^+^ THY1^+^ population is highly enriched in stem cell activity, and *in vitro* assays showed that these cells have the capacity to form short-term germinal clusters. The stem cell activity of this population (187 colonies/10^5^ transplanted donor cells) indicates that the combination of the 4 markers used here improved the definition of this stem cell subpopulation compared with previous strategies (Dovey et al., 2013; Valli et al., 2014). Comparison of the transcriptome of the β-2M^−^SP^+^α-6^+^THY1^+^ population with that of scRNA-seq studies of human spermatogonia (Guo et al., 2018; Sohni et al., 2019) and that of RNA-seq of PLPPR3^+^ spermatogonia (Tan et al., 2020) suggests that this population contains the primitive states of human spermatogonia.

We identified genes sets and a network of TRs that are preferentially expressed in this population of human undifferentiated spermatogonia highly enriched for SSCs. Some have been reported previously to be relevant for maintenance of SSC and spermatogonial progenitors in mice (e.g., PIWIL4, FGFR2, GFRA1, ADGRA3/GPR125, PIK3CA, SALL1, SALL3, TEC, ZBTB33, and ABCG2/BCRP1). Gene Ontology annotations highlighted cell migration and motility as top biological processes in human β-2M^−^SP^+^α-6^+^THY1^+^ cells. Migration of SSCs over the seminiferous tubules niche is observed frequently in mice and plays a role in determination of their fate toward self-renewal or differentiation (Nakagawa et al., 2010). TGF/BMP/SMAD, PEDF, EMT, and the embryonic stem cell pluripotency pathways were among the most highly regulated in human primitive spermatogonia. Interestingly, PEDF has been reported to be secreted by human testicular peritubular cells (Windschüttl et al., 2015). EMT is a highly conserved cellular process that involves transformation of epithelial cells into mesenchymal cells and has been implicated in embryogenesis, tissue repair, and tumorigenesis. EMT signaling via stringent attachment of SSCs and spermatogonial progenitors to the basement membrane could play a role in their physiologic status and cell fate determination. NDRG1 and G-CSF signaling, predicted to be potential top upstream regulators of primitive spermatogonia, belong to pathways involved in regulation of murine SSCs (Kanatsu-Shinohara et al., 2016; Kotzur et al., 2017). Inflammatory signaling, known to affect the hematopoietic stem cell fate (Pietras, 2017), has also been found to be relevant to human β-2M^−^SP^+^α-6^+^THY1^+^ SSCs.

SSC maintenance was affected in culture *in vitro* when *Hes1* expression was reduced. Constitutive downregulation of *Hes1* can affect stem cells and can lead to neuronal differentiation (Liu et al., 2015; Kageyama et al., 2008). Ectopic constitutive expression of human HES1 has a protective effect in murine SSCs and progenitors when they are exposed to medium starvation conditions and poised for quiescence, as seen in other cell types (Sang et al., 2008). Targeted overexpression of HES1 has been shown previously to have a survival effect in melanoblasts by protecting the cells from elimination by apoptosis (Moriyama et al., 2006), in line with our observations on cell death in SSC culture. Hence, the role of HES1 in the SSC physiology, at least *in vitro*, questions the potential role of the NOTCH pathway in SSCs, which remains controversial (Hasegawa et al., 2012; Huang et al., 2013; Garcia et al., 2017). Other factors, such as FGF2, can also induce HES1 expression (Sato et al., 2010). Although the mechanisms involved in the function of HES1 need further investigation, especially by *in vivo* analyses, our results suggest that HES1 plays a role in the physiology of murine SSCs.

Studies using high-throughput RNA profiling to comprehensively analyze gene expression in spermatogenesis will increase our understanding of the molecular pathways involved in germ cell differentiation and promote identification of the molecular defects responsible for infertility. Deciphering the molecular pathways that regulate self-renewal and proliferation in SSCs could also help with developing and improving culture conditions for *in vitro* amplification of human SSCs, which is critically important for applying SSCs in therapeutic treatments.

## Experimental procedures

For details, see the supplemental experimental procedures.

### Experimental model and human materials

Adult human testis biopsies were obtained from individuals with obstructive azoospermia with normal spermatogenesis who consented to inclusion in this study (institutional review board [IRB]-approved protocol IRB 00003835, 2012/40ICB).

### Flow cytometry analysis of testicular single-cell suspensions

Testicular single-cell suspensions were prepared from human biopsies. Vybrant staining (1 μg/mL) and immunolabeling of α-6 integrin, THY1, and β2M of the cell suspensions were performed as described previously (Barroca et al., 2009; Corbineau et al., 2017). This protocol was applied to 16 human donors in this study, providing representative flow cytometry profiles as illustrated in Figure 1A.

### Transcriptome

RNA samples were analyzed using an Affymetrix Human Gene 2.1 ST Array and an Affymetrix GeneChip Mouse Gene 2.0 ST Array (Thermo Fisher Scientific).

### Statistics

All values are shown as mean ± SEM. Statistical analyses were performed by Student’s t test (GraphPad Prism software): ns, not significant, p > 0.05; ^∗^p < 0.05; ^∗∗^p < 0.01; ^∗∗∗^p < 0.001.

### Data and code availability

The accession number for the microarray data reported in this paper is GEO: GSE155509.

## Author contributions

M.G., V.F., B.L., V.B.-L., and P.F. provided project design. M.G., V.F., B.L., and P.F. performed experiments and analyzed and interpreted data. A.S.G., C.L., I.H., A.J., F.H., I.A., and L.R. participated in experimentation and provided comments. F.D., S.J., and F.L. participated in microarray and bioinformatics analyses. F.P. provided NSG mice. N.T., C.P., J.P.W., and V.B.-L. supervised sample acquisition. M.G., V.F., and P.F. prepared the manuscript.

## Conflicts of interest

The authors declare no competing interests.

## Supplemental information

Document S1. Supplemental experimental procedures, Figures S1–S6, and Tables S1–S8

Table S1. List of DEGs enriched in the β-2M^−^α-6^med^THY1^−^-“S-phase”, spermatocyte I (spI) and spermatocyte II (spII) populations at the spermatocyte I/β-2Mα-6^med^THY1^−^-“S-phase” and spermatocyte II/spermatocyte I cell state transitions according to their chromosomal positions

Table S2. List of DEGs at the different transitions of germinal cell states (A) List of DEGs in the β-2M^−^SPα-6^+^THY1^+^ (T^+^), β-2M^−^SPα-6^med^THY1^−^ (T^−^), spermatocyte I (4N), spermatocyte II (2N), and spermatid (N) populations at the different transitions of cell states (fc>2, p<0.02). (B) List of DEGs in the β-2M^−^SPα-6^+^THY1^+^ population (fc>2, p<0.02). (C) List of transcriptional regulators (TR) that varied across spermatogenesis and preferentially enriched in the β-2M^−^SPα-6^+^THY1^+^ population (fc>2, p<0.02).

Table S7. Comparison of DEGs in murine and human spermatogonia (A) List of DEGs from comparison between the undifferentiating spermatogonia (KIT-) and the differentiating spermatogonia (KIT+) populations in mice (fc>1.5, p<0.02). (B) List of genes conserved between the human β-2M^−^SPα-6^+^THY1^+^ and murine β-2M^−^SPα-6^+^c-kit- cell populations (fc>1.5, p<0.02 for both lists).

Document S2. Article plus supplemental information
