# Supplementary material for: Transcriptional profiling of β-2M−SPα-6+THY1+ spermatogonial stem cells in human spermatogenesis
Source: Stem Cell Reports. 2022 Mar 24;17(4):936–52. doi: 10.1016/j.stemcr.2022.02.017 (PMC9023810; doi:10.1016/j.stemcr.2022.02.017)
Supplement: Document S1. Supplemental experimental procedures, Figures S1–S6, and Tables S1–S8 [file mmc1.pdf]

Stem Cell Reports, Volume *17*

Supplemental Information

Transcriptional profiling of β-2M^{-}SPα-6^{+}THY1^{+} spermatogonial stem cells in human spermatogenesis

Maelle Givelet, Virginie Firlej, Bruno Lassalle, Anne Sophie Gille, Clementine Lapoujade, Isabelle Holtzman, Amandine Jarysta, Farahd Haghighirad, Florent Dumont, Sébastien Jacques, Franck Letourneur, Françoise Pflumio, Isabelle Allemand, Catherine Patrat, Nicolas Thiounn, Jean Philippe Wolf, Lydia Riou, Virginie Barraud-Lange, and Pierre Fouchet

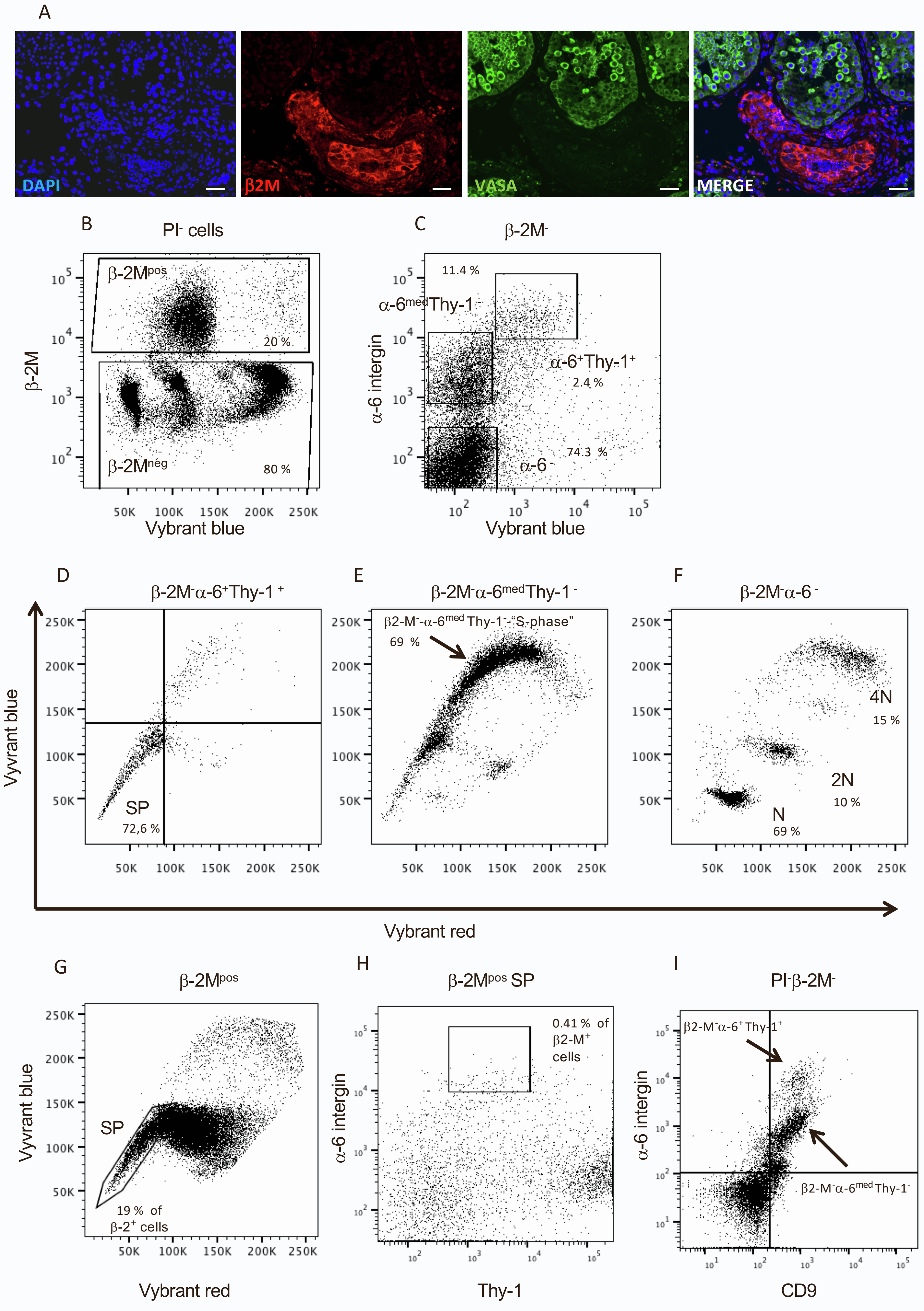

A
DAPI
β2M
VASA
MERGE
B
PI- cells
β-2Mpos
20 %
β-2Mnég
80 %
β-2M
Vybrant blue
C
β-2M-
11.4 %
α-6medThy-1-
α-6+Thy-1+
2.4 %
74.3 %
α-6-
α-6 intergin
Vybrant blue
D
β-2M-α-6+Thy-1+
SP
72,6 %
E
β-2M-α-6medThy-1-
β2-M--α-6med Thy-1- "S-phase"
69 %
F
β-2M-α-6-
4N
15 %
2N
10 %
N
69 %
Vyvrant blue
Vybrant red
G
β-2Mpos
SP
19 % of
β-2+ cells
Vyvrant blue
Vybrant red
H
β-2Mpos SP
0.41 % of
β2-M+
cells
α-6 intergin
Thy-1
I
PI-β-2M-
β2-M-α-6+Thy-1+
β2-M-α-6med Thy-1-
α-6 intergin
CD9

Figure S1

Supplemental Figure S1: β2M^{-} α-6^{+}THY1^{+} cells are mainly found in the SP population. (A) Immunofluorescent analysis of β2M expression in human testicular tissue. β-2M fluorescent signal was observed in the interstitial tissue, but not in DDX4-positive germ cells, scale bar: 20 microns. (B) β2M expression and Vybrant blue fluorescence in viable human testicular cells (PI-negative). (C) THY1 and α-6 integrin expression in β2M-negative cells. β2M$^{-}\alpha$-6^{+}THY1^{+}, β2M^{-}SPα-6^{med}THY1^{-}, and α-6^{neg} cells are indicated. (D) (E) (F) Vybrant blue and red fluorescence in β2M$^{-}\alpha$-6^{+}THY1^{+} (D), β2M^{-} α-6^{med}THY1^{-} (E), and α-6^{neg} populations (F) as defined in Fig. S1C. Side population (SP); meiotic and postmeiotic subpopulations of spermatocyte I (4N DNA content), spermatocyte II (2N DNA content), and spermatid (N DNA content) cells, and "β2M^{-}-α-6^{med}THY1^{-}-"S-phase" cells are indicated. (G) Vybrant blue and red fluorescence in β2M-positive testicular cells as defined in Fig. S1B. (H) THY1 and α-6 integrin expression in β2M^{+}SP cells. Low frequency of somatic SPα-6^{+}THY1^{+} cells were also detected in the β-2M+ population (I) CD9 and α-6 integrin expression in viable PI$^{-}\beta$2M^{-} cells. Cells corresponding to β2M$^{-}\alpha$-6^{+}THY1^{+} and β2M$^{-}\alpha$-6^{med}THY1^{-} populations are indicated.

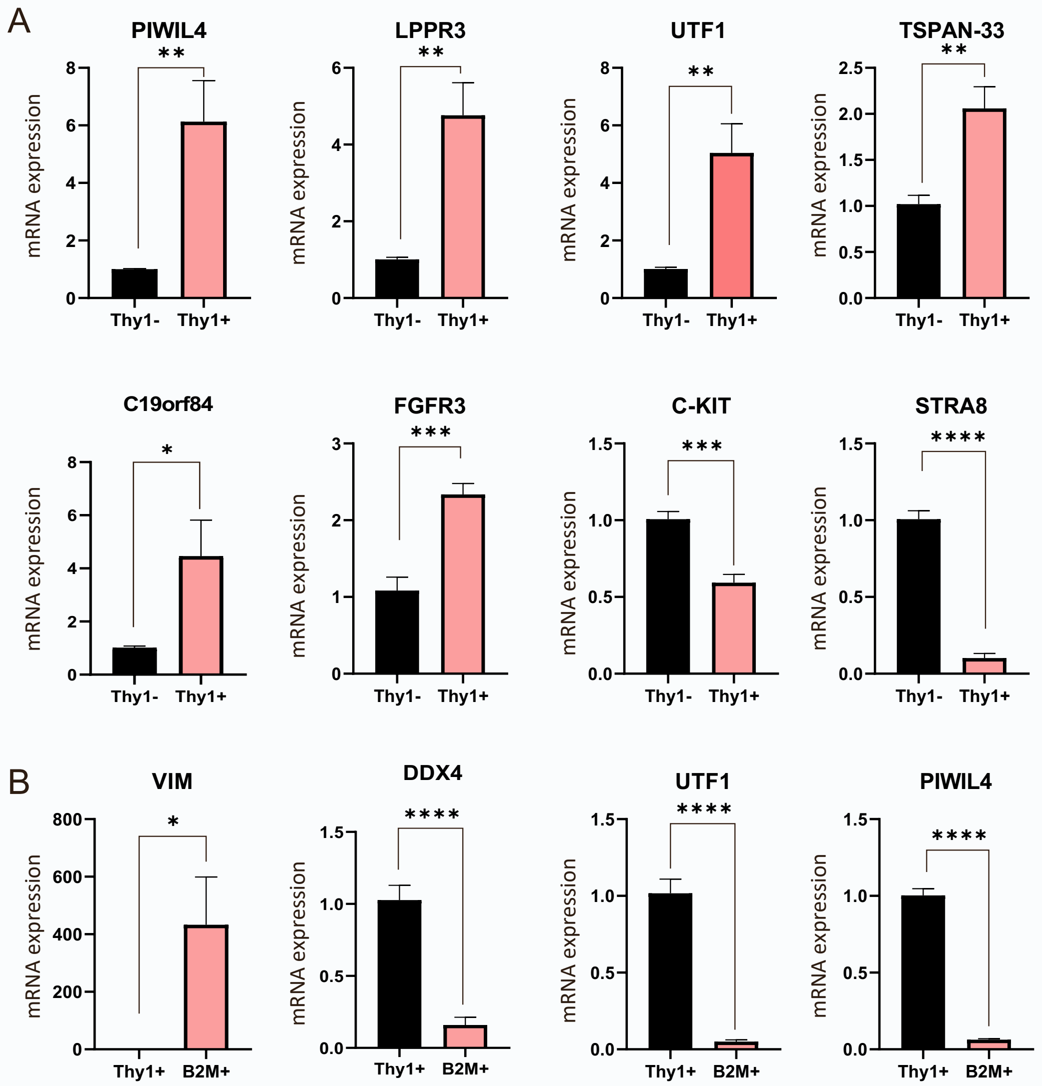
A
PIWIL4
LPPR3
UTF1
TSPAN-33
C19orf84
FGFR3
C-KIT
STRA8
mRNA expression
Thy1-
Thy1+
B
VIM
DDX4
UTF1
PIWIL4
Thy1+
B2M+

Figure S2

Supplemental Figure S2: (A) Analysis by RT-qPCR of the expression of *PIWIL4* (n=4), *C19orf84* (n=5), *TSPAN33* (n=6), *PLPPR3* (n=5), *FGFR3* (n=5), *UTF1* (n=5), *KIT*(n=5) and *STRA8* (n=5) gene spermatogonial markers in the β-2M$^-$SPα-6$^+$THY1$^+$ (T$^+$) and β-2M$^-$SPα-6^{med}THY1$^-$ (T$^-$) populations (pool of RNA from 3 different donors), (B) Analysis by RT-qPCR of the expression of the somatic *VIM* gene marker (n=5), and of *DDX4/VASA* (n=6), *PIWIL4* (n=4) and *UTF1* (n=5) gene spermatogonial markers in the β-2M$^-$SPα-6$^+$THY1$^+$ (Thy1$^+$) and β-2M$^+$ (B2M$^+$) populations. (pool of RNA from 2 different donors)

A

Symbol	FC
CABS1	51.10
LINC01760	48.08
LOC105376980	39.29
LOC101928317	28.75
LYZL6	25.25
FBXW10	24.39
LOC105374836	21.98
LINC01766	21.11
ACTRT2	21.03
LINC01921	19.86
MCHR2-AS1	19.60
PLAAT5	19.06
LOC107983959	17.83
ACTL7A	17.75
GTSF1L	17.05
FAM209A	15.72
IQCF4	15.58
LINC00919	15.04
CAPZA3	14.84
TMCO2	14.68
CCDC54	14.57
LOC107986582	14.48
HMGB4	14.44
ZC2HC1B	14.21
P3R3URF	14.08

C

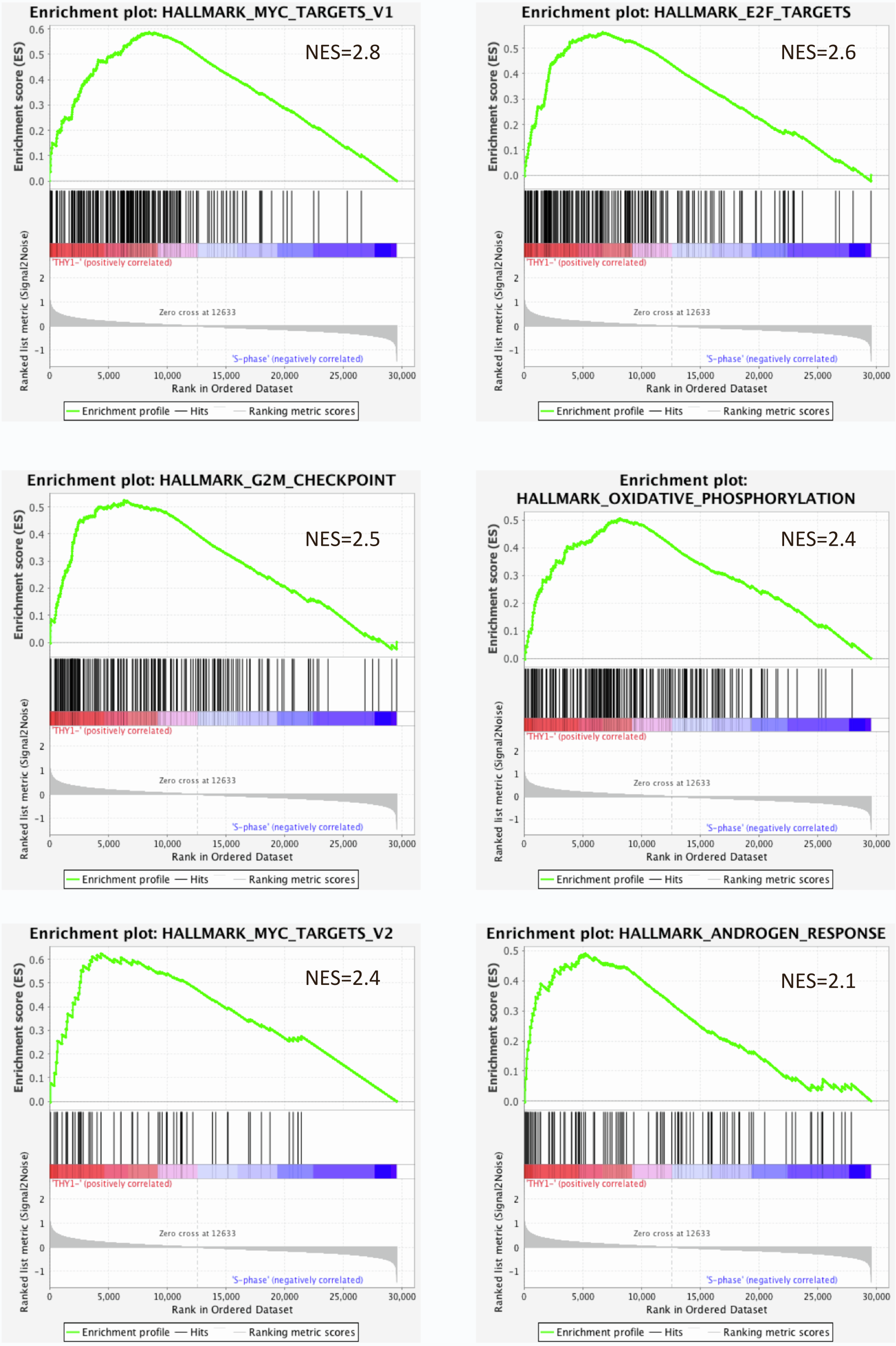

B

Symbol	FC
SNORD13P1	17.63
LINC02116	12.04
FAM197Y9	10.83
MIR4324	10.61
H3-4	9.10
FBXO47	8.33
LINC02460	7.53
HESX1	7.34
UBR5-AS1	7.17
HOTAIR	5.46
GEMIN8P4	5.31
HOXC5	5.07
RAD51AP2	5.06
HLA-B	5.02
RAB41	4.83
OTUD6A	4.78
TNFAIP8L3	4.66
LOC105375605	4.58
LOC100499489	4.57
OR3A2	4.56
SPO11	4.52
USP6	4.43
PRSS41	4.32
PRSS38	4.25
LOC105372210	4.20

D

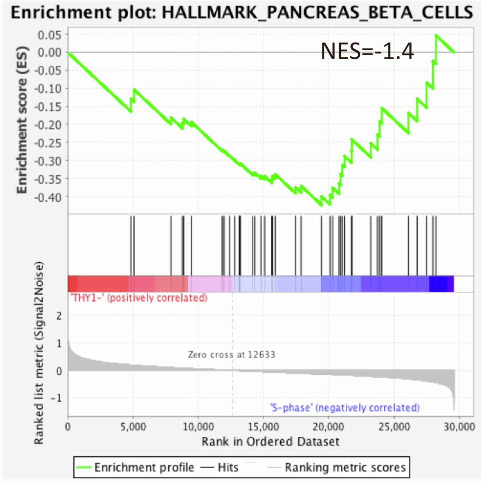

Figure S3

Supplemental Figure S3: Expression signature from comparison of β-2M$^-$ SPα-6^{med} THY1$^-$ with β-2M$^-$$\alpha$-6^{-med}THY1$^-$-"S-phase" spermatogonial cells

(A)Top-25 list of differentially expressed genes in β-2M$^-$ SPα-6$^+$THY1$^-$, (B) Top-25 list of differentially expressed genes in β-2M$^-$$\alpha$-6^{-med}THY1$^-$-"S-phase". (C and D) GSEA enrichment plots from GSEA hallmark analysis of pathways (C) in β-2M$^-$ SPα-6^{med}THY1$^-$ (FDR=0) and (D) in β-2M$^-$$\alpha$-6^{-med}THY1$^-$-"S-phase" (FDR $\leq$ 0.05).

A

Symbol	FC
FBXW10	88,29
LOC107986582	74,37
MCHR2-AS1	58,63
HHLA3-AS1	49,91
TMIGD3	47,68
PLAAT5	46,85
LINC02619	41,48
LINC00882	38,88
ROPN1L-AS1	38,36
AQP5	36,96
HSD52	36,45
LOC101928721	34,38
LINC01766	32,94
C9orf57	32,25
CABS1	32,12
LOC105375843	31,73
LINC00911	31,37
LOC100507071	31,36
LOC100240728	31,33
LOC105378044	30,23
ZNF385D	30,06
PPFIA2-AS1	29,30
ACR	29,17
TMEM225	28,70
ZC2HC1B	28,40

B

Symbol	FC
SSX3	56,14
H2AC20	50,20
ZNF280C	28,83
TEX19	25,77
STEAP1	25,36
FAM197Y9	25,22
H2AC8	24,89
HPRT1	24,26
H3C11	22,87
CT45A10	22,24
PAGE1	22,14
PRR20G	19,81
WDR44	19,21
CCNB3	19,17
ATP2B1	18,67
H13	18,63
CT55	17,53
PRDM7	17,36
CDC6	17,07
GBA	16,95
PRSS38	16,88
FBXO5	16,22
MAGEC1	16,05
ATP11C	15,88
BEND2	15,26

C

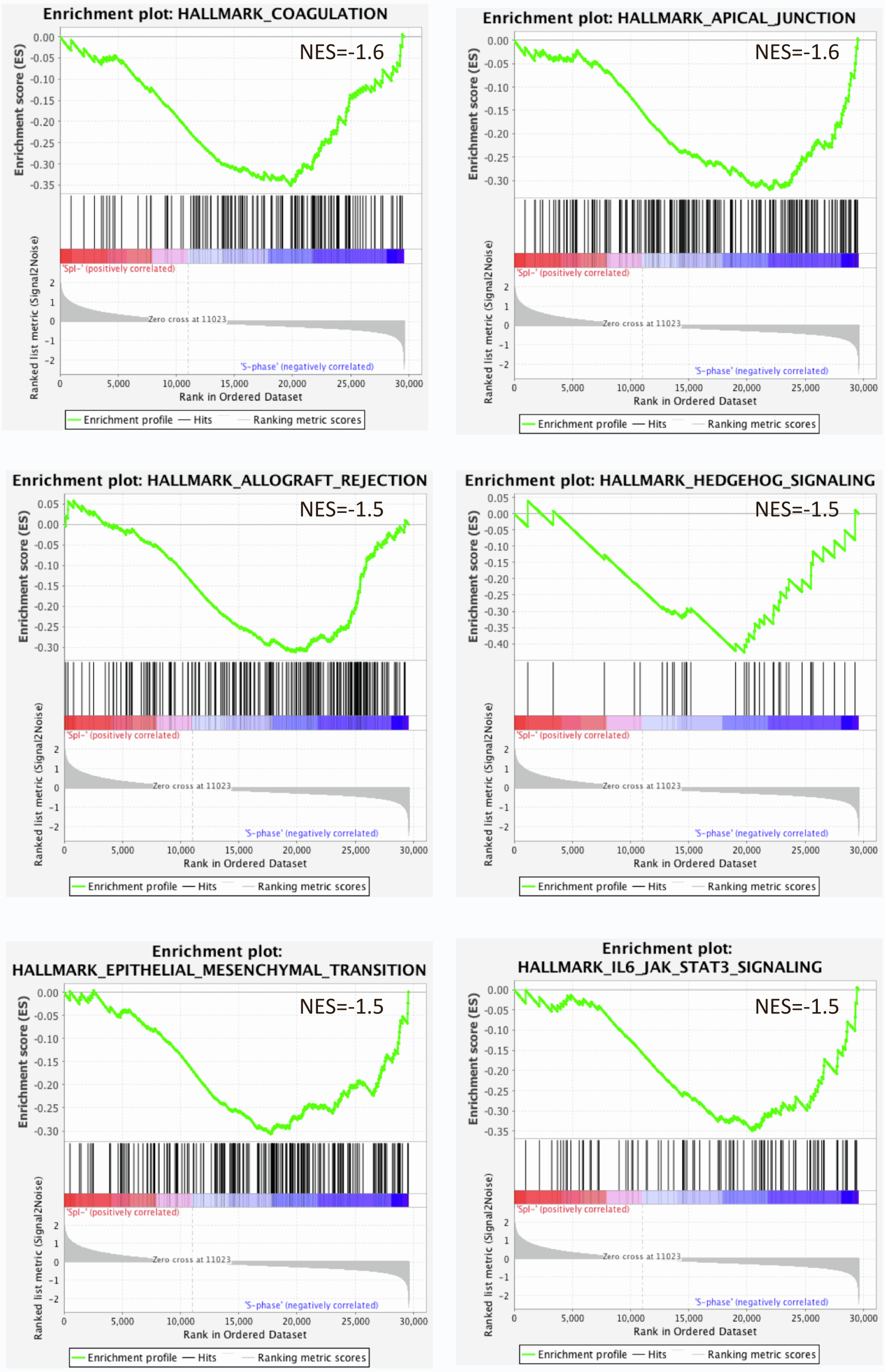

D

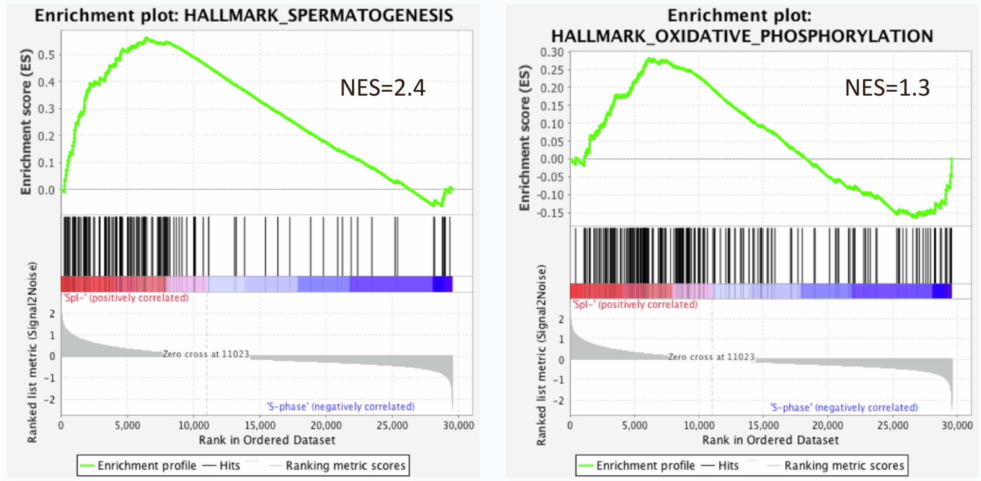

Figure S4

Supplemental Figure S4: Expression signature from comparison of β-2M$^{-}\alpha$-6^{-med}THY1^{-}-“S-phase” with spermatocyte I cells.

(A)Top-25 list of differentially expressed genes in β-2M$^{-}\alpha$-6^{-med}THY1^{-}-“S-phase”, (B) Top-25 list of differentially expressed genes in spermatocyte I. (C and D) GSEA enrichment plots from GSEA hallmark analysis of pathways (C) in β-2M$^{-}\alpha$-6^{-med}THY1^{-}-“S-phase” (FDR<0.1) and (D) in spermatocyte I (FDR <0.3).

A

Symbol	FC
H16	57,24
C9orf57	55,85
H34	53,14
H2AC1	40,28
GOLGA2P11	32,67
SELENOT	28,85
HLTF	23,08
MAD2L1	21,23
LOC100507384	20,86
H2BC1	20,15
C4orf46	19,95
TDRG1	19,85
LOC105370612	18,75
MARK2P9	18,17
NBPF1	18,00
LINC00865	17,61
LINC02721	17,25
LINC02475	17,22
LOC105369649	16,92
LOC105370613	16,10
IQCB1	15,89
H11	15,67
C18orf63	15,35
NAE1	14,54
TSNAX	14,16

B

Symbol	FC
LINC00524	24,56
LOC101927269	22,39
LINC00411	21,87
LOC105370739	21,45
LOC101928093	21,01
LINC01919	20,95
LOC107984561	19,96
LOC107985743	18,41
LOC105370248	17,99
CACNA1C-IT3	17,79
LOC105372038	17,18
LOC102724080	16,26
LOC105369714	15,89
LOC105371010	15,70
DIAPH1-AS1	14,47
LOC105374258	14,40
LOC105378885	14,39
LINC01717	14,11
LOC105375559	14,07
OXCT2	13,90
LOC105371371	13,80
HSFX4	13,74
LOC105375377	13,73
LOC105371342	13,71
LOC105372750	13,70

C

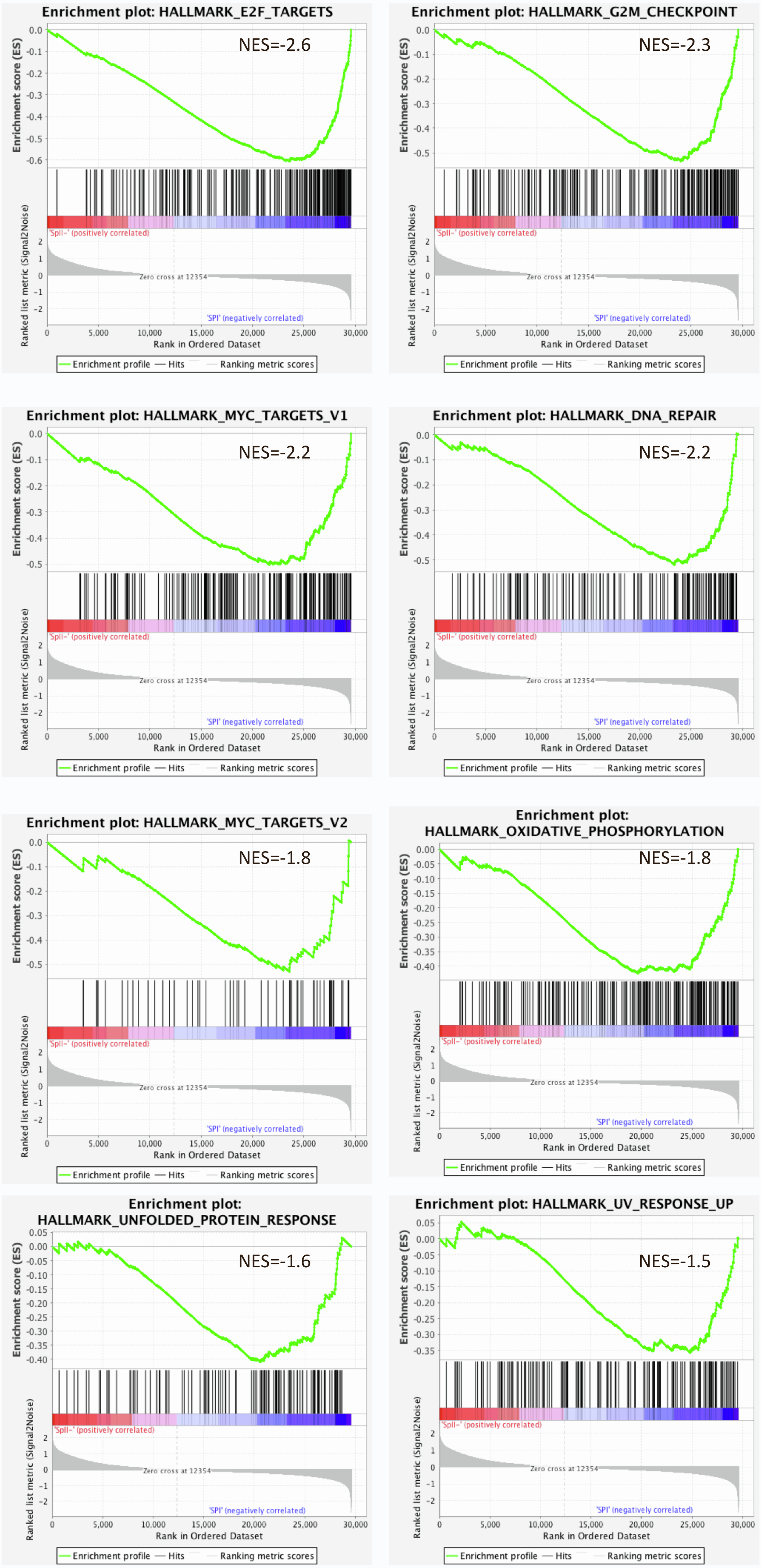

Figure S5

Supplemental Figure S5: Expression signature from comparison of spermatocyte I with spermatocyte II cells.

(A)Top-25 list of differentially expressed genes in spermatocyte I, (B) Top-25 list of differentially expressed genes in spermatocyte II (C) GSEA enrichment plots from GSEA hallmark analysis of pathways in spermatocyte I (FDR ≤ 0.005). No enrichment of pathways was found in spermatocyte II with FDR<1 and NES>1.3.

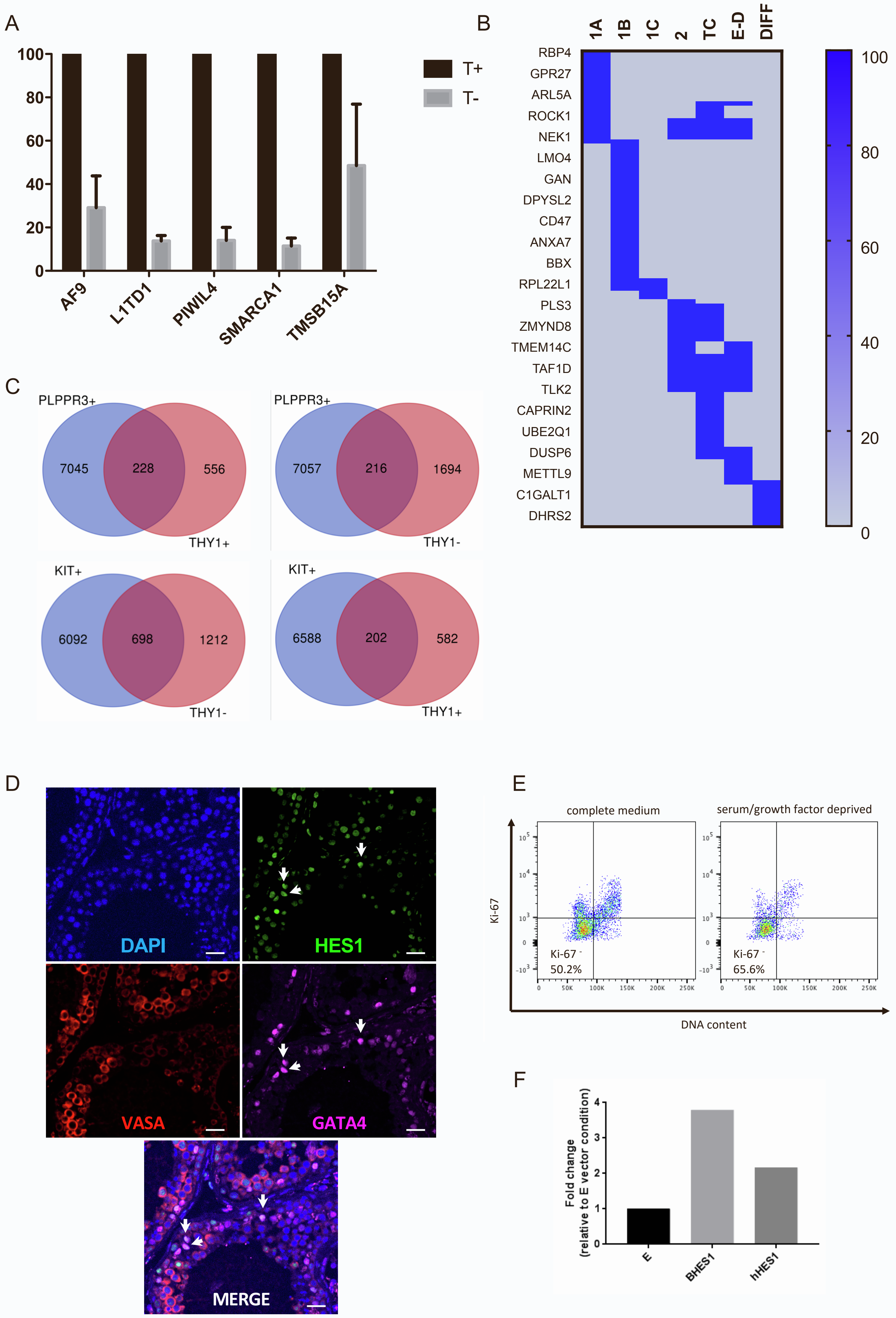

A
100
80
60
40
20
0
T+
T-
AF9
L1TD1
PIWIL4
SMARCA1
TMSB15A
B
1A
1B
1C
2
TC
E-D
DIFF
RBP4
GPR27
ARL5A
ROCK1
NEK1
LMO4
GAN
DPYSL2
CD47
ANXA7
BBX
RPL22L1
PLS3
ZMYND8
TMEM14C
TAF1D
TLK2
CAPRIN2
UBE2Q1
DUSP6
METTL9
C1GALT1
DHRS2
100
80
60
40
20
0
C
PLPPR3+
7045
228
556
THY1+
PLPPR3+
7057
216
1694
THY1-
KIT+
6092
698
1212
THY1-
KIT+
6588
202
582
THY1+
D
DAPI
HES1
VASA
GATA4
MERGE
E
complete medium
serum/growth factor deprived
Ki-67
Ki-67 -
50.2%
Ki-67 -
65.6%
DNA content
F
Fold change
(relative to E vector condition)
4
3
2
1
0
E
BHES1
hHES1

Figure S6

Supplemental Figure S6: Analysis of genes enriched in the β2M$^-$SPα-6$^+$THY1$^+$ transcriptome

(A) Validation by qRT-PCR of genes found to be overexpressed in β2M$^-$SPα-6$^+$THY1$^+$ (T$^+$) compared to β2M$^-$SPα-6^{med}THY1$^-$ (T$^-$) on Affymetrix microarray. (B) Heatmap showing the distribution of the expression of genes in β2M$^-$SPα-6$^+$THY1$^+$ population according to the gene clusters defining the different states in the human adult SSC development as defined by Sohni et al., {Sohni et al., 2019, #56519}. Clusters SSC 1A, 1B, 1C and 2 describing stem cell population, transition cells (TC), Early differentiating spermatogonia (ED) and Differentiating spermatogonia (DIFF) (Sohni et al., 2019). (C) Venn diagram showing the relationships between the transcriptomes of human β-2M$^-$SPα-6$^+$THY1$^+$, β-2M$^-$SPα-6^{med}THY1$^-$, PLPPR3$^+$ (Tan et al., 2020) and KIT$^+$ (Tan et al., 2020) spermatogonial populations. The number of genes in each group is indicated. (D) Immunofluorescent detection of HES1 and GATA-4 in human testes from obstructive azoospermia patient: DAPI (Blue), HES1 (green), VASA (red), GATA-4 (magenta), arrows (HES1-positive Sertoli cells), scale bar: 20 microns. (E) Ki-67/DNA content analysis of SSCs cultured in complete serum or under serum/growth factor-deprived conditions. (F) mRNA expression of *HES1* and *ΔBHES1* in SSCs transduced with *HES1* (HES1), *ΔBHES1* (BHES1), and *GFP* control (E) vectors.

Supplemental Table S1: List of DEGs enriched in the β–2M$^{-}\alpha$-6^{med}THY1^{-}-“S-phase”, spermatocyte I (spI) and spermatocyte II (spII) populations at the spermatocyte I/β–2M^{-} α-6^{med}THY1^{-}-“S-phase” and spermatocyte II/spermatocyte I cell state transitions according to their chromosomal positions.

Supplemental Table S2: (A) List of DEGs in the β–2M^{-}SPα-6^{+}THY1^{+} (T^{+}), β-2M^{-}SPα-6^{med}THY1^{-} (T^{-}), spermatocyte I (4N), spermatocyte II (2N), and spermatid (N) populations at the different transitions of cell states (fc>2, p<0.02). (B) List of DEGs in the β–2M^{-}SPα-6^{+}THY1^{+} population (fc>2, p<0.02). (C) List of transcriptional regulators (TR) that varied across spermatogenesis and preferentially enriched in the β–2M^{-}SPα-6^{+}THY1^{+} population (fc>2, p<0.02).

GO cellular component complete	fold Enrichment	raw P-value	FDR
filopodium (GO:0030175)	4.45	4.86E-06	4.24E-04
lamellipodium (GO:0030027)	3.32	4.96E-06	4.14E-04
growth cone (GO:0030426)	2.84	2.20E-04	1.34E-02
site of polarized growth (GO:0030427)	2.73	3.45E-04	1.82E-02
RNA polymerase II transcription factor complex (GO:0090575)	2.73	7.56E-04	3.52E-02
nuclear transcription factor complex (GO:0044798)	2.51	8.26E-04	3.76E-02
actin-based cell projection (GO:0098858)	2.50	8.67E-04	3.86E-02
cell leading edge (GO:0031252)	2.45	6.41E-06	5.14E-04
secretory granule lumen (GO:0034774)	2.36	2.06E-04	1.29E-02
cytoplasmic vesicle lumen (GO:0060205)	2.33	2.39E-04	1.41E-02

Supplemental Table S3: Cellular component annotations of the β–2M$^-$SPα-6$^+$THY1$^+$ gene list obtained according to the GO PANTHER classification

GO molecular function	fold Enrichment	raw P-value	FDR
protein binding involved in heterotypic cell-cell adhesion (GO:0086080)	14.51	1.62E-05	2.47E-03
Rac guanyl-nucleotide exchange factor activity (GO:0030676)	9.24	4.67E-04	4.91E-02
Rho guanyl-nucleotide exchange factor activity (GO:0005089)	5.59	2.04E-04	2.41E-02
transcription cofactor binding (GO:0001221)	5.13	3.41E-04	3.84E-02
cell-cell adhesion mediator activity (GO:0098632)	4.93	4.34E-04	4.66E-02
transmembrane receptor protein kinase activity (GO:0019199)	4.12	1.64E-04	2.05E-02
DNA-binding transcription repressor activity, RNA polymerase II-specific (GO:0001227)	3.12	1.79E-06	3.84E-04
DNA-binding transcription repressor activity (GO:0001217)	3.12	1.79E-06	3.67E-04
protein tyrosine kinase activity (GO:0004713)	3.03	4.22E-04	4.64E-02
phosphatase binding (GO:0019902)	2.84	2.20E-04	2.54E-02

Supplemental Table S4: Molecular function annotations of the β–2M^{-}SPα-6^{+}THY1^{+} gene list obtained according to the GO PANTHER classification

NAME	NES	NOM p-val	FDR q-val
HALLMARK_UV_RESPONSE_DN	1.854	0.000	0.003
HALLMARK_INFLAMMATORY_RESPONSE	1.803	0.000	0.005
HALLMARK_COAGULATION	1.784	0.000	0.004
HALLMARK_ALLOGRAFT_REJECTION	1.693	0.000	0.007
HALLMARK_KRAS_SIGNALING_UP	1.689	0.000	0.006
HALLMARK_ANGIOGENESIS	1.647	0.007	0.008
HALLMARK_EPITHELIAL_MESENCHYMAL_TRANSITION	1.619	0.000	0.009
HALLMARK_TGF_BETA_SIGNALING	1.614	0.012	0.008
HALLMARK_HEDGEHOG_SIGNALING	1.543	0.019	0.015
HALLMARK_COMPLEMENT	1.485	0.005	0.024
HALLMARK_IL6_JAK_STAT3_SIGNALING	1.482	0.015	0.023
HALLMARK_APICAL_SURFACE	1.406	0.053	0.046
HALLMARK_INTERFERON_GAMMA_RESPONSE	1.402	0.008	0.044
HALLMARK_NOTCH_SIGNALING	1.388	0.077	0.046
HALLMARK_KRAS_SIGNALING_DN	1.374	0.018	0.049
HALLMARK_HYPOXIA	1.321	0.023	0.074

<u>Supplemental Table S5:</u> Pathways enriched in the β–2M$^-$SPα-6$^+$THY1$^+$ population (GSEA analysis).

NAME	NES	NOM p-val	FDR q-val
HALLMARK_SPERMATOGENESIS	-2.862	0.000	0.000
HALLMARK_MYC_TARGETS_V1	-2.608	0.000	0.000
HALLMARK_E2F_TARGETS	-2.561	0.000	0.000
HALLMARK_OXIDATIVE_PHOSPHORYLATION	-2.251	0.000	0.000
HALLMARK_G2M_CHECKPOINT	-2.104	0.000	0.000
HALLMARK_DNA_REPAIR	-2.061	0.000	0.000
HALLMARK_MYC_TARGETS_V2	-1.999	0.000	0.000
HALLMARK_UV_RESPONSE_UP	-1.828	0.000	0.001
HALLMARK_UNFOLDED_PROTEIN_RESPONSE	-1.752	0.000	0.002

Supplemental Table S6: Pathways enriched in the β-2M^{-} SPα-6^{med} THY1^{-} population (GSEA analysis)

Supplemental Table S7: (A) List of DEGs from comparison between the undifferentiating spermatogonia (KIT-) and the differentiating spermatogonia (KIT+) populations in mice (fc>1.5, p<0.02). (B) List of genes conserved between the human β-2M^{-}SPα6^{+}THY1^{+} and murine β-2M^{-}SPα-6^{+}c-kit- cell populations (fc>1.5, p<0.02 for both lists).

gene	primer	5'-3'
AF9	F	CAACGTTACCGCCATTTG
	R	GTCTGGGATGGTGTGAAG
CXCL9	F	GCATCATCTTGCTGGTTCTGATTGG
	R	GCGACCCTTTCTCACTACTGGGGT
L1TD1	F	TCCCACAAAAGGAAGAAATAAATC
	R	GCTCTATGCTTTGAGTCTATTAGGG
PIWIL4	F	AATGCTCGCTTTGAACTAGAGAC
	R	ATTTTGGGGTAGTCCACATTAAATC
SMARCA1	F	ACGGCCTCCAAAACAGCCAAATG
	R	TGAGCCAGAGCTGGATTTGGGATA
TMSB15A	F	CCGCGAACAGCCTTTCAC
	R	CGACAAGTCTGGCTTATCACTCA
GAPDH	F	GTCGGAGTCAACGGATTTGG
	R	AGCAGCCCTGGTGACCAG
NANOS2	F	GGCTGGAGATGTTGAGAGCAA
	R	AAAGGAAATCCAGTGGCGC
GFRA1	F	GGGAGAAGCCCAACTGTTTG
	R	GACAGCTGCTGACAGACCTTGA
ABCG2	F	CAGGAGGCCTTGGGATACTT
	R	GCTATAGAGGCCTGGGGATT
ID4	F	GAGCCGCGCTGTCCAGGTGTG
	R	CTGCTCTTCCCCCTCCCTCTCTAGT
PLZF	F	AGCGGTTCCTGGATAGTTTGC
	R	TTCGAAAACTGTGCACCACACT
NANOS2	F	GTCTTCGCAGGCTCACCT
	R	GGCATTGAAAGGTGTCAGC
VASA	F	GAAGCTGATCGCATGTTGGATA
	R	TGCAGCCAACCTTTGAATTTC
RFX4	F	CCCGGTCCAAACTCGGAAC
	R	TGGCTCTTATTACAGTGTCCAGT
RFX2	F	CTATGGGATTCGTCTGAAGCC
	R	GGAGACATCTATGTACTGCTGGT
TNP2	F	CAGAGTTGCAGACAGAGCCAT
	R	TCATAGTCTTTTTGTGGCGCTT
CREM	F	ACACCACCTAGTATTGCTACCA
	R	GGATTGTTCCACCTTGGGCTAT
GUS	F	CCGAGTGAAGATCCCCTTTTTA
	R	CTCATTTGGAATTTTGCCGATT
HES1	F	CCAGCCAGTGTCAACACGA
	R	AATGCCGGGAGCTATCTTTCT

TSPAN33	F	CCGCTGGTGAAATACCTGCTC
	R	AGGGCTGCTTCTGCATGCTT
LPPR3[1]	F	CTTCTGCCCTGCTTCTACTTCG
	R	CATAGCACTGGAAGCCCACC
UTF1[1]	F	CGGCTCCCAGCGAACCAG
	R	GACGGGCTGAAGCGGAGC
FGFR3[1]	F	CCGAGCGGATGGACAAGAAG
	R	GACCAGGCTCCACTGCTGAT
VIM[1]	F	GGACCAGCTAACCAACGACAAAG
	R	CTCTCTCTGAAGCATCTCCTCCT
PIWIL4[1]	F	CATCAAGTTCTCCCGTGTGC
	R	GACACAGAAATGGCAAACCC
C19orf84[1]	F	AGATGGAACAACCAAAGGACG
	R	GTTCAGGAGCAAGGGTGGAG
KIT	F	GGAAGCCTCTTCCCAAGGAC
	R	GCTGGCCTCACTTTCAGGAT
DDX4/VASA[3]	F	AAGAGAGGCGGCTATCGAGATGGA
	R	CGTTCACTTCCACTGCCACTTCTG
NANOS3[2]	F	ACGCTTCTGCCCACTTAC
	R	TTCTTGCCTGCCGAGTTT
STRA8[4]	F	AATCCCATGACAGAGCAAC
	R	TTATCCAGGGTTTGCTCCAG

[1]From Sohni et al., 2019; [2]From Hermann et a, 2018, [3] From Anderson et al., Conserved and divergent patterns of expression of DAZL, VASA and OCT4 in the germ cells of the human fetal ovary and testis. *BMC Dev Biol.* **7**, 136 (2007),[4] From Medrano et al., Human somatic cells subjected to genetic induction with six germ line-related factors display meiotic germ cell-like features Scientific report, 6, 24956 (2016).

Supplemental Table S8: Primer list

Supplemental Experimental procedures

Experimental model and human materials

Adult human testis biopsies from obstructive azoospermia patients with normal spermatogenesis were obtained from the CECOS Hospital Cochin. All patients consented to inclusion in this research study (IRB-approved protocol: IRB 00003835; 2012/40ICB; French Institutional Review Board-Comité de Protection des Personnes, Ile de France IV). The C57Bl6/J, FVB/N, and immunodeficient NOD/Shi-scid/IL-2Rγ^{null} (NSG) mice were housed in our animal facility. All experiments were performed in compliance with European legislation and the guidelines of the Ethics Committee of the French Ministry of Agriculture (Agreement B9203202). All animal-related procedures were performed in compliance with the European Communities Council Directive of 22th September 2010 (EC/2010/63) and were approved by Comité d'Ethique en Expérimentation Animale, Direction de la Recherche Fondamentale, CEA (authorization 14-081; CEtEA-CEA DRF IdF).

Testicular single-cell suspensions, immunomagnetic and flow cell sorting, and flow cytometry analysis

Testicular single-cell suspensions were prepared from human biopsies. The tissue was incubated in trypsin 0.25% containing collagenase I (final concentration, 0.5 mg/ml) for 20 minutes at 34°C. The cell suspension was then filtered (20 μm). The vital DNA dye Vybrant, which was previously shown to identify cells with the SP phenotype during hematopoiesis, was used instead of Hoechst 33342, owing to the wavelength

excitation maxima (369 nm) of the Vybrant fluorophore (Telford et al., 2007). Vybrant staining (1 µg/ml) of the cell suspensions was performed as previously described (Barroca et al., 2009; Corbineau et al., 2017), and cells were labeled with anti-α6 integrin-PE (GoH3) (BD Pharmingen), β2M-FITC (BD Pharmingen) and anti-THY1-APC (BD Pharmingen) antibodies. Propidium iodide (Sigma) was added before cell sorting to exclude dead cells. Vybrant efflux inhibition was performed by pre-incubating human testicular cells (10^6 cells/ml) for 30 minutes at 32°C in incubation medium supplemented with the specific BCRP1 inhibitor Ko143 (200 nM). The effect of the Ko143 on the SP phenotype was estimated using the formula (% SP^{Ctrl} - % SP^{Ko143} /%SP^{Ctrl}) X100.

Murine testicular single-cell suspensions were prepared from 2- to 3-month-old FVB/N mice as previously described (Barroca et al., 2009; Corbineau et al., 2017). The immunomagnetic selection of $\alpha6^{+}$ cells was performed using anti-α6 integrin-PE (GoH3) (BD Pharmingen) and anti-PE (Miltenyi Biotec) microbeads according to the manufacturers' protocols. Hoechst staining (5 µg/ml) of the cell suspensions was performed. The cells were then labeled with β2m-FITC (Santa Cruz) and anti-CD117-APC (2B8) antibodies (BD Pharmingen). Propidium iodide (Sigma) was added before cell sorting to exclude dead cells (Barroca et al., 2009; Corbineau et al., 2017). Single-cell suspensions were analyzed using a FACSCalibur™ or LSRII flow cytometer system (BD Biosciences). BD Trucount™ Tubes (BD 340334) were used to assess the number of germinal cells and dead cells using propidium iodide and flow cytometry in SSC cultures. The data were analyzed with DIVA or FlowJo software. Cell sorting was performed using a FACSAria cytometer (BD Biosciences).

RNA extraction and quantitative RT-PCR

mRNA was prepared using RNeasy® Micro and Mini kits (Qiagen). The mRNA was then reverse-transcribed with a Quantitect kit (Qiagen). Quantitative RT-PCR was performed using an AB7900 device (Applied Biosystems) with Fast SYBR® Green Master Mix (Applied Biosystems). The primers are listed in Table S8.

SSC and MEF cultures

Murine adult SSC lines were obtained from C57Bl6/J and EGFP mice (Okabe et al., 1997) and maintained on mitomycin C-treated mouse embryonic fibroblasts (MEFs) as previously described (Barroca et al., 2009; Corbineau et al., 2017). The SSC culture medium was composed of Stem Span (Stemcell Technologies) and B27 supplement (Life Technologies) and supplemented with recombinant human GDNF (40 ng·ml^{-1}, R&D Systems), recombinant rat GFRA1 (300 ng·ml^{-1}, R&D Systems), FGF2 (1 ng·ml^{-1}, Life Technologies), and ES-CultTM Fetal Bovine Serum (1%, Stemcell Technologies). Every 3-4 days, the SSC clusters were split *via* enzymatic digestion with 0.05% trypsin-EDTA (Life Technologies). MEF cultures were established *via* trypsin digestion of 13.5 days post-coital (dpc) embryos, and the resulting cells were cultured in DMEM supplemented with 10% FBS, L-glutamine and penicillin/streptomycin. Human β-2M$^-$SP$^+\alpha$-6$^+$Thy-1$^+$ SSCs were maintained on gelatin-coated plates in SSC culture medium.

Hes1 expression was knocked down using siRNAs (5′-CGACACCGGACAAACCAAA-3′). SSCs culture were dissociated and 1 × 10^5 cells were transfected with *Hes1* siRNAs or negative control siRNAs by electroporation at 1400 V for 20 ms (2 pulses) using a Neon Transfection System and the Neon® Transfection System 10 µL Kit

(Thermo Fisher Scientific) according to the manufacturer's instructions. Transfected cells were then plated on MEFS in 96-well plates.

Lentiviral vector production and transduction of cells in SSC cultures

Viral particles were produced *via* transient transfection of 293T cells with the following lentiviral vectors: wt HES1, ∆BHES1 (gift from L. Chen, obtained from Addgene) (Yu et al., 2006), and pTRIP-GFP. The packaging plasmids were pCMVDR-8.92 and pMD2G. Viral supernatants were collected 48–72 h later. SSCs were infected by exposing the SSCs to viral supernatants overnight in SSC medium containing 5 μg ml^{-1} polybrene. The SSCs were then plated on mitomycin C-treated MEFs and expanded after selection *via* cell sorting based on GFP fluorescence.

Human and Mouse Testis Cell Transplantation

NSG and C57BL6J mice were used as the recipients for human and mouse cell transplantation, respectively. To deplete endogenous spermatogenesis, the recipient mice were treated at 6-8 weeks of age with busulfan (40 mg/kg at least 4 weeks before donor cell transplantation). Human or murine donor cells were resuspended in DMEM supplemented with 10% heat-inactivated fetal calf serum, 100 mg/ml DNase I (DN25, Sigma-Aldrich) and 4% trypan blue solution (T8154, Sigma-Aldrich) for transplantation as previously described (Barroca et al., 2009). A 10-microliter solution of donor cells was introduced into the seminiferous tubules of the testis of the recipient mouse *via* an injection through the efferent ductules as previously described. Ten weeks after transplantation, the recipient testis were collected and analyzed by either macroscopic observation of fluorescence in mice or immunohistochemistry in human tissues as described below. The capacity of donor-cells to home, proliferate and give rise to high

number of spermatogonial progeny in the recipient testis is linked to the stem cell potential. The colonies containing the higher number of spermatogonial progeny must originate from the most primitive spermatogonia, with the highest stem cell potential. For human, two categories of cell clusters were arbitrarily defined, one likely derived from donor-cells with a lower regenerative potential (4-8 cell clusters) and one derived from donor-cells with higher regenerative potential (>8 cells). Macroscopic observations of recipient testis tissues were performed using an Olympus epifluorescence microscope to detect the presence of EGFP-fluorescent seminiferous tubules.

Histology and immunofluorescence

In human cell analyses, the recipient testis were fixed in 4% paraformaldehyde, frozen in Tissue-Freezing medium (OCT, Sakura, Netherlands), and sectioned at a thickness of 10 microns. The sections were stained overnight at 4°C with primary antibodies (see below) against human nuclei (MAB 1281, Millipore), MAGEA4 (HPA021942, Sigma-Aldrich), PLZF (sc22839, Santa-Cruz Biotech), VASA (ab13840, Abcam), or HES1 (ab119776, Abcam). Then, the sections were incubated for 2 h at room temperature with the corresponding secondary antibodies (Life-Technology, OR, USA), donkey anti-mouse Alexa-fluor 488 and donkey anti-rabbit Alexa-fluor 594, respectively. Cell nuclei were counterstained with 4',6-diamidino-2-phenylindole (DAPI). VASA/β2M and HES1/GATA4/VASA expression analysis were performed on formalin-fixed paraffin-embedded sections of human testis with primary antibodies against β2M (ab175031, Abcam), VASA (ab13840, Abcam), HES1 (ab196328, Abcam) and GATA4 (sc1237, Santa Cruz Biotech), and then with the corresponding secondary antibodies. Imaging

was performed using an Olympus AX70 epifluorescence microscope equipped with a CoolSNAP Myo camera (Photometrics) in Micro-Manager software (version 1.4.16, open-source microscopy) or a Nikon A1 laser fluorescence confocal microscope with NIS Elements software (version 4.51, Nikon).

Transcriptome

From several donors, three cell samples were sorted for each differentiation stage, in order to prepare RNA to be subsequently hybridized to Affymetrix high-density oligonucleotide chips. Germinal populations were directly sorted *via* flow cytometry in lysis buffer obtained from an RNeasy Micro kit (Qiagen). The quantity and quality of the RNA were analyzed using a 21000 Bioanalyzer (Agilent Technologies). Only samples with an RNA integrity number (RIN) equal to or above 7 were used for the transcriptomic analyses. RNA samples were then analyzed using an Affymetrix® Human Gene 2.1 ST Array and an Affymetrix GeneChip™ Mouse gene 2.0 ST Array (Thermo Fisher Scientific). Data were robust multi-array average normalized with R/Bioconductor, and ANOVA test was applied to extract DEGs. Data were analyzed using Panther (http://www.pantherdb.org) (Mi et al., 2019), Ingenuity Pathway Analysis, and String (http://string-db.org) (Szklarczyk et al., 2019) were used. Gene set enrichment analysis (GSEA) was also performed as described previously (Subramanian et al., 2005). GEO accession number for microarray data: GSE155509

Additional References

Mi, H., Muruganujan, A., Ebert, D., Huang, X., and Thomas, P.D. (2019) PANTHER version 14: more genomes, a new PANTHER GO-slim and improvements in enrichment analysis tools. Nucleic Acids Res, *47*, D419–D426.

Okabe, M., Ikawa, M., Kominami, K., Nakanishi, T., and Nishimune, Y. (1997) Green mice' as a source of ubiquitous green cells. FEBS Lett, *407*, 313–319.

Subramanian, A., Tamayo, P., Mootha, V.K., Mukherjee, S., Ebert, B.L., Gillette, M.A., Paulovich, A., Pomeroy, S.L., Golub, T.R., Lander, E.S. et al. (2005) Gene set enrichment analysis: a knowledge-based approach for interpreting genome-wide expression profiles. Proc Natl Acad Sci U S A, *102*, 15545–15550.

Szklarczyk, D., Gable, A.L., Lyon, D., Junge, A., Wyder, S., Huerta-Cepas, J., Simonovic, M., Doncheva, N.T., Morris, J.H., Bork, P. et al. (2019) STRING v11: protein-protein association networks with increased coverage, supporting functional discovery in genome-wide experimental datasets. Nucleic Acids Res, *47*, D607–D613.

Telford, W.G., Bradford, J., Godfrey, W., Robey, R.W., and Bates, S.E. (2007) Side population analysis using a violet-excited cell-permeable DNA binding dye. Stem Cells, *25*, 1029–1036.
