# Supplementary material for: Transcriptional profiling of β-2M−SPα-6+THY1+ spermatogonial stem cells in human spermatogenesis
Source: Stem Cell Reports. 2022 Mar 24;17(4):936–52. doi: 10.1016/j.stemcr.2022.02.017 (PMC9023810; doi:10.1016/j.stemcr.2022.02.017)
Supplement: Document S2. Article plus supplemental information [file mmc5.pdf]

Stem Cell Reports
Resource
OPEN ACCESS

Transcriptional profiling of β-2M^{-}SPα-6^{+}THY1^{+} spermatogonial stem cells in human spermatogenesis

Maelle Givelet,[1,2,9] Virginie Firlej,[1,2,9] Bruno Lassalle,[1,9] Anne Sophie Gille,[1,2] Clementine Lapoujade,[1] Isabelle Holtzman,[2] Amandine Jarysta,[1] Farahd Haghighirad,[3] Florent Dumont,[5] Sébastien Jacques,[6] Franck Letourneur,[6] Françoise Pflumio,[7] Isabelle Allemand,[1] Catherine Patrat,[3,4] Nicolas Thiounn,[8] Jean Philippe Wolf,[3,4] Lydia Riou,[1] Virginie Barraud-Lange,[3,4,9] and Pierre Fouchet[1,9,*]
[1]Université de Paris and Université Paris-Saclay, CEA, UMR Stabilité Génétique Cellules Souches et Radiations, iRCM/IBFJ, Laboratoire des Cellules Souches Germinales, 92265 Fontenay-aux-Roses, France
[2]Institut Cochin, INSERM U1016, Département de Génétique, Développement et Cancer, Équipe Génomique Epigénétique et Physiopathologie de la Reproduction, 75014 Paris, France
[3]UFR Médecine Paris Centre-Université de Paris, 15 rue de l'école de Médecine, 75006 Paris, France
[4]Assistance Publique-Hôpitaux de Paris, Hôpitaux Universitaires Paris Centre, CHU Cochin, Histologie-Embryologie-Biologie de la Reproduction, 75014 Paris, France
[5]Université Paris Saclay, UMS IPSIT, 92296 Châtenay-Malabry, France
[6]Université de Paris, Institut Cochin, INSERM, U1016, CNRS UMR8104, Plateforme Séquençage et Génomique, 75014 Paris, France
[7]Université de Paris and Université Paris-Saclay, INSERM, CEA, UMR Stabilité Génétique Cellules Souches et Radiations, iRCM/IBFJ, LSHL, 92265 Fontenay-aux-Roses, France
[8]Department of urology and transplant surgery, Hôpital européen Georges-Pompidou, AP-HP, Université de Paris, 20 rue Leblanc, 75015 Paris, France
[9]These authors contributed equally
*Correspondence: pierre.fouchet@cea.fr
https://doi.org/10.1016/j.stemcr.2022.02.017

SUMMARY

Male infertility is responsible for approximately half of all cases of reproductive issues. Spermatogenesis originates in a small pool of spermatogonial stem cells (SSCs), which are of interest for therapy of infertility but remain not well defined in humans. Using multiparametric analysis of the side population (SP) phenotype and the α-6 integrin, THY1, and β-2 microglobulin cell markers, we identified a population of human primitive undifferentiated spermatogonia with the phenotype β-2 microglobulin (β-2M)$^{-}$SPα-6^{+}THY1^{+}, which is highly enriched in stem cells. By analyzing the expression signatures of this SSC-enriched population along with other germinal progenitors, we established an exhaustive transcriptome of human spermatogenesis. Transcriptome profiling of the human β-2M^{-}SPα-6^{+}THY1^{+} population and comparison with the profile of mouse undifferentiated spermatogonia provide insights into the molecular networks and key transcriptional regulators regulating human SSCs, including the basic-helix-loop-helix (bHLH) transcriptional repressor HES1, which we show to be implicated in maintenance of SSCs *in vitro*.

INTRODUCTION

Throughout a male's reproductive life, the pool of spermatogonial stem cells (SSCs) can self-renew or differentiate before undergoing meiosis and then spermiogenesis to produce sperm. SSCs constitute a potential source of cells that can be used to develop regenerative medicines for infertility, especially after cancer treatments during childhood. Testicular transplantation of SSCs has been found to lead to efficient production of functional sperm and restoration of fertility in several animal models (Brinster, 2007), including non-human primates (Hermann et al., 2012).

In mice, the best-characterized mammal model, SSCs are a subpopulation of A_{single} (A_s) spermatogonia in the adult testis (de Rooij, 2017). These stem cells can self-renew or differentiate into committed paired (A_p) and aligned (A_{al}) spermatogonia, which are collectively called undifferentiated spermatogonia. However, this hierarchical model has been questioned because of recent evidence showing some equipotency in terms of regenerative potential among GFRA1^{+} undifferentiated spermatogonia, with stemness apparently not restricted to only A_s spermatogonia (Hara et al., 2014). Compared with mice, identification of the SSC pool and the molecular mechanisms governing their self-renewal and differentiation remains largely elusive in humans. The prevailing model holds that human spermatogenesis arises from A_{dark} and A_{pale} spermatogonia, which are thought to represent reserve and active stem cells, respectively (Ehmcke and Schlatt, 2006). Recent single-cell RNA sequencing (scRNA-seq) studies greatly contributed to highlight the heterogeneity of the premeiotic spermatogonial population and helped to define different cell states in the population of primitive spermatogonia (Wang et al., 2018; Guo et al., 2018; Hermann et al., 2018; Sohni et al., 2019). As illustrated by the previous studies describing several cell surface proteins enabling selection of populations of human spermatogonia with repopulation potential after testicular transplantation (Zohni et al., 2012; Dovey et al., 2013; Nickkholgh et al., 2014; Valli et al., 2014; Tan et al., 2020;

© 2022 The Authors.
This is an open access article under the CC BY license (http://creativecommons.org/licenses/by/4.0/).

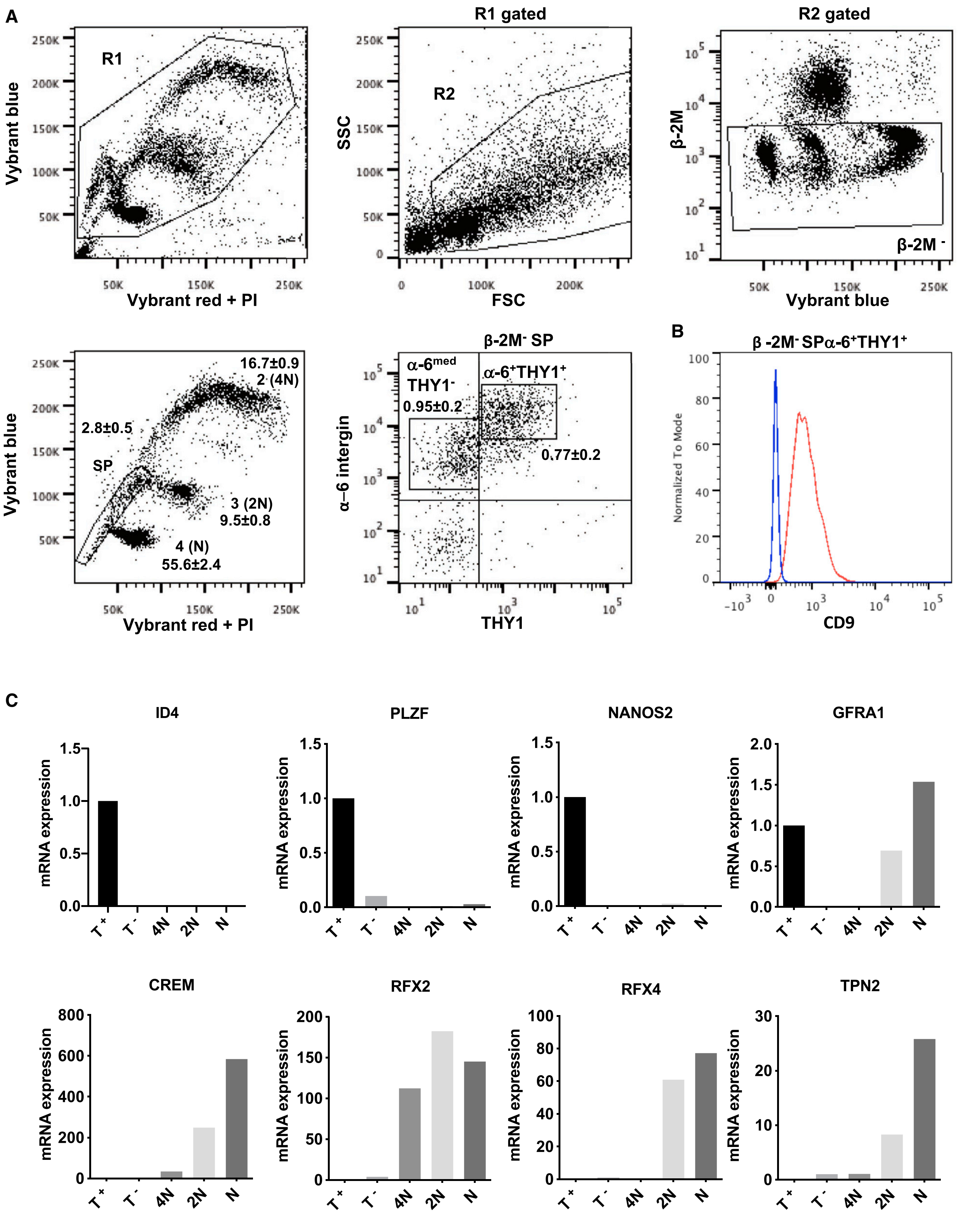

(legend on next page)

Shami et al., 2020), reliable phenotypic markers that allow purification and study of populations of stem cells and progenitors are of significant importance to understand the self-renewal of human SSCs.

Here we developed a fluorescence-activated cell sorting (FACS)-based method that enabled us to purify a population of primitive undifferentiated spermatogonia, highly enriched in SSCs, and five premeiotic, meiotic, and postmeiotic populations of human germ cells. We established an exhaustive transcriptome of human spermatogenesis and examined the dynamics of gene expression throughout the developmental process. This study provides insights into the molecular networks potentially implicated in regulation of primitive spermatogonia and SSCs in humans.

RESULTS

Characterization of adult human spermatogenesis using β-2 microglobulin, side population, α-6 integrin, THY1, and DNA content markers

We and others have shown previously that, in mice, SSCs and spermatogonial progenitors express α-6 integrin and Thy-1 and harbor the side population (SP) phenotype according to an analysis of vital DNA dye efflux by the (ATP Binding Cassette) ABC transporter *Bcrp1/Abcg2* (Lassalle et al., 2004; Falciatori et al., 2004; Barroca et al., 2009). Assuming that mouse and human models of SSCs and spermatogonial progenitors share some characteristics, we applied this multi-parameter flow cytometry strategy (Figure 1A) to characterize human spermatogenesis using this set of markers and the pan-somatic marker β-2 microglobulin (Figure S1A). Viable cells (gate R1) were analyzed using forward and side scatter to remove elongated spermatids and sperm from further analyses (gate R2). After gating for β-2 microglobulin (β-2M)$^-$ cells to remove somatic cells, we were able to resolve the complex Vybrant red and blue fluorescently labeled cells into five major cell populations, including an SP containing cells that actively excluded the DNA dye. Subpopulations 2 (4N DNA content), 3 (2N DNA content), and 4 (N DNA content) were assumed to correspond to spermatocyte I, spermatocyte II, and haploid (N) spermatids cells, respectively. To further this characterization, the SP population was divided based on α-6 integrin and THY1 expression. β-2M$^-$α-6$^+$THY-1$^+$ cells and β-2M$^-$α-6^{med}THY1$^-$ cells represented, respectively, 0.77% ± 0.2% and 0.95% ± 0.2% of the β-2M$^-$ cell population (n = 5). Using the opposite gating scheme, we confirmed that β-2M$^-$α-6$^+$THY1$^+$ cells were found mainly in the SP population and represented 69.7% ± 2.7% (n = 11) of the SP cells (Figures S1B–S1H). β-2M$^-$SPα-6$^+$THY1$^+$ and β-2M$^-$ SP α-6^{med} THY1$^-$ cells also expressed the human spermatogonial marker CD9 (Figures 1B and S1I). In addition to expression of spermatogonial markers *ID4*, *NANOS2*, *PLZF*, and *GFRA1* (Figure 1C), we found that *PIWIL4*, *C19orf84*, *TSPAN33*, *PLPPR3*, *FGFR3*, and *UTF1* primitive spermatogonial markers were expressed in the β-2M-SPα-6$^+$THY1$^+$ population, whereas *KIT* and *STRA8* differentiating markers were expressed in the β-2M-SPα-6^{med}THY1$^-$ population (Figure S2A). The vimentin somatic marker was not detected in β-2M$^-$SPα-6$^+$THY1$^+$ cells, showing that the β-2M$^-$SPα-6$^+$THY1$^+$ population is not composed of somatic cells (Figure S2B). On the other hand, vimentin was highly expressed in the β-2M$^+$ cell population (Figures S1B and S2B), and the pan-germinal *VASA* (*DDX4*) marker and *FGFR3* and *UTF1* undifferentiated spermatogonial markers were detected at very low levels, the β-2M$^+$ population being composed of somatic cells. The lower expression level of α-6 integrin observed in the β-2M$^-$SP α-6^{med}THY1$^-$ fraction, in addition to the lack of expression of markers of primitive spermatogonia and meiotic cells (Figures 1C and S2A), indicated that these cells were in a more advanced differentiation stage and represented a differentiating spermatogonial population. The expression levels of meiotic markers (*CREM*, *RFX2*, *RFX4*, and *TNP2*) suggested that subpopulations 2 (4N), 3 (2N), and 4 (N) corresponded to the spermatocyte I, spermatocyte II, and spermatid populations, respectively. These data show that the β-2M$^-$SPα-6+THY1$^+$ population contains primitive spermatogonia and lacks somatic cells.

The human spermatogonial SP phenotype depends on ABC transporter activity of the *BCRP1/ABCG2* gene

The SP phenotype is caused by active efflux of DNA dye via members of the ABC transporter superfamily. Specifically,

Figure 1. Characterization of the different steps of human spermatogenesis

(A) Characterization of the different steps of human spermatogenesis by flow cytometry according to the forward scatter (FSC), side scatter (SSC), blue and red Vybrant fluorescence, β-2M, α-6 integrin, and THY1 parameters as well as side population (SP) and meiotic and postmeiotic subpopulations 2 (4N), 3 (2N), and 4 (N). The frequency (percentage) of the subpopulations in the whole β-2M$^-$ population is indicated (mean ± SEM, n = 5).

(B) CD9 expression in the β-2M$^-$ SPα-6$^+$ THY1$^+$ population (red line). The control (blue line) corresponds to the signal in CD9$^-$/low-expressing postmeiotic round spermatids.

(C) Analysis by qRT-PCR of the expression of different spermatogonial (*ID4*, *NANOS2*, *PLZF*, and *GFRA1*) and meiotic (*CREM*, *RFX2*, *RFX4*, and *TNP2*) markers in meiotic and postmeiotic subpopulations 2 (4N), 3 (2N), and 4 (N) and the spermatogonial β-2M$^-$SPα-6$^+$THY1$^+$ (T$^+$) and β-2M$^-$SPα-6^{med}THY1$^-$ (T$^-$) populations

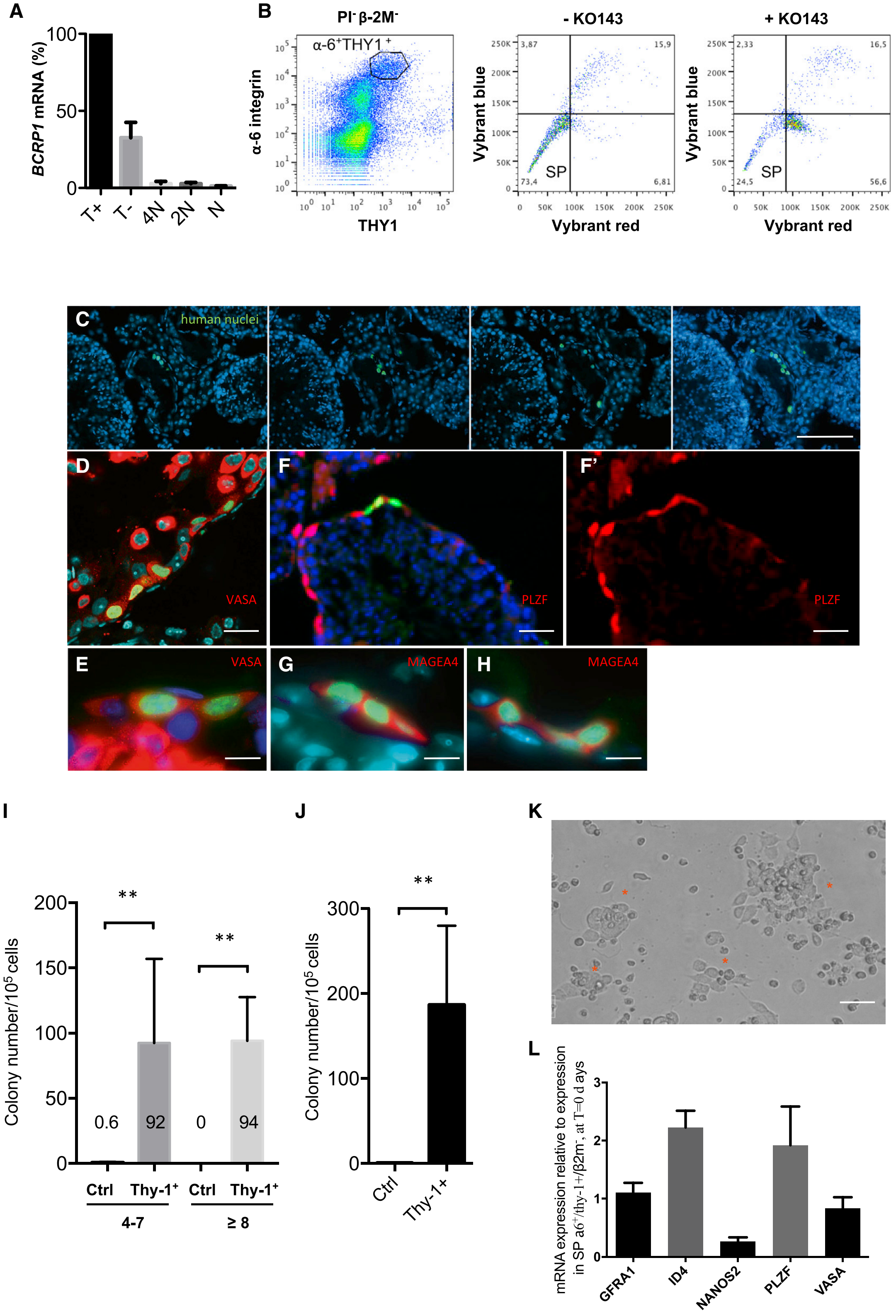

(legend on next page)

BCRP1/ABCG2, an ABC transporter, has been shown to play a major role in development of the SP phenotype in stem and progenitor cells during murine hematopoiesis and spermatogenesis and in human cancer stem-like cells (Zhou et al., 2001; Lassalle et al., 2004; Bleau et al., 2009). We found that the *BCRP1/ABCG2* gene was expressed in the β-2M$^-$SPα-6$^+$THY1$^+$ population of primitive spermatogonia (Figure 2A). In the presence of the specific BCRP1 inhibitor Ko143 (Allen et al., 2002), Vybrant efflux was markedly reduced in the β-2M$^-$SPα-6$^+$ THY1$^+$ population (Figure 2B) and resulted in a reduction of the SP population by 68.5% ± 2%. These results suggest that BCRP1 transporter activity is involved in the human germinal SP phenotype.

In adults, the β-2M$^-$SPα-6$^+$THY1$^+$ population of primitive spermatogonia is highly enriched in human SSCs

Transplantation assays are a critical tool to test the capacity of putative SSCs to regenerate spermatogenesis after transplantation into germ cell-depleted testes. When transplanted, human SSCs show only a limited capacity to proliferate and differentiate in the testes of immunodeficient mice. However, these experiments allow evaluation of the potential of transplanted putative SSCs to migrate, survive, and colonize and are currently the gold-standard test for human SSCs (Dovey et al., 2013; Valli et al., 2014). β-2M$^-$SPα-6$^+$THY1$^+$ cells obtained from human testicular biopsies were sorted and transplanted (3,700–9,600 cells per testis) into busulfan-depleted testes of *NOD$^-$Scid/IL2Rγc$^{-/-}$* (NSG) humanized mice. As a control, a population including all germinal cells excluding SPα-6$^+$ THY1^{+-} cells (i.e., all β-2M$^-$ cells excluding SPα-6$^+$ THY1$^+$ cells) was sorted and transplanted (39,600–83,000 cells per testis). The rate of colonization by human SSCs was estimated via immunofluorescence analysis using an antibody against human DNA nuclear protein in serial sections of recipient testes (Bissig-Choisat et al., 2015). Clusters of human cells residing close to the basal membrane were observed 2 months after transplantation (Figure 2C). The germinal markers VASA (Figures 2D and 2E) and MAGEA4 (Figures 2G and 2H) were expressed in all cells from the human cluster, and 78% ± 10% of the cells in these clusters (n = 8 clusters) were positive for PLZF (Figures 2F and 2F′), indicating that the cells derived from human β-2M$^-$SPα-6$^+$THY1$^+$ donor cells were mainly spermatogonia (Eildermann et al., 2012). The clusters were counted and separated into two classes according to the number of cells per cluster (4–7 cells and ≥8 cells). These data show that human SSCs, having the capacity to colonize murine recipient testes, are found in the β-2M$^-$SPα-6$^+$ THY1$^+$ population and that germinal populations that do not have this phenotype (all β-2M$^-$ cells except SPα-6$^+$ THY1$^+$ cells) are devoid of SSCs (Figure 2I). Hence, the β-2M$^-$SPα-6$^+$THY1$^+$ population is highly enriched in human SSCs. The stem cell activity of this population (187 colonies/10^5 donor cells; Figure 2J) appeared to fall within the same range but was roughly 4-fold higher than what has been reported in previously published studies, approximately 50 colonies/10^5 donor cells when using EPCAM or α6-integrin as markers (Dovey et al., 2013; Valli et al., 2014). Human SSCs can be cultured *in vitro* for 2–3 weeks (Medrano et al., 2016). Germinal cluster formation was observed clearly only in wells seeded with β-2M$^-$SPα-6$^+$ THY1$^+$ cells in the presence of the growth factors glial cell line-derived neurotrophic factor (GDNF) and fibroblast growth factor 2 (FGF2) up to 2 weeks of culture (Figure 2K). Cell clusters continued to express markers of immature spermatogonia after 15 days of culture (Figure 2L). These data show that

Figure 2. The SP phenotype is BCRP1/ABCG2 dependent, and the β-2M$^-$SPα-6$^+$THY1$^+$ (T$^+$) population possesses SSC potential
(A) The BCRP1/ABCG2 gene is expressed in the T$^+$ subpopulation. Analysis by qRT-PCR of the expression of *BCRP1* in T$^+$, T$^-$, spermatocyte I (4N), spermatocyte II (2N), and spermatid (N) populations (n = 3 experiments).
(B) Ko-143 inhibits the SP phenotype in the T$^+$ subpopulation. The frequencies of the populations are indicated.
(C) Serial sections of testes obtained from NSG mice 2 months after transplantation with T$^+$ cells. Human cells (green, antibody specific to human nuclei) observed at the murine basement membrane on successive sections indicate human colonizing cell clusters.
(D and E) VASA (red) expression in human cells (green nuclei) from colonizing cell clusters.
(F and F′) PLZF expression in human cells (green, human nuclei; red, PLZF) from colonizing cell clusters.
(G and H) MAGE-4 expression in human cells (green, human nuclei; red, MAGEA-4 Blue indicates 4′,6-diamidino-2-phenylindole (DAPI) from colonizing cell clusters.
(I) Comparison of the colonization efficiency of recipient testes transplanted with T$^+$ cells or control β-2M$^-$not(SPα-6$^+$ THY1$^+$) cells. Human colonizing cell clusters were separated in two groups according to their size: one group composed of cluster of 4–7 cells and the other of cluster of 8 or more cells (control, n = 4 recipient testes; THY1$^+$, n = 5 recipient testes; transplantations from five human donors).
(J) The total number of cell clusters generated per 10^5 cells injected (control, n = 4 recipient testes; THY1$^+$, n = 5 recipient testes; transplantations from five human donors).
(K) Cell clusters observed *in vitro* after 14 days of culture starting with the T$^+$ cell population.
(L) Expression of markers of immature spermatogonia in cell clusters after 15 days of culture (n = 6 replicates from 2 independent cultures). Scale bars: 50 μm (C), 20 μm (D), 40 μm (F, F′, and K), and 10 μm (E, G, and H).

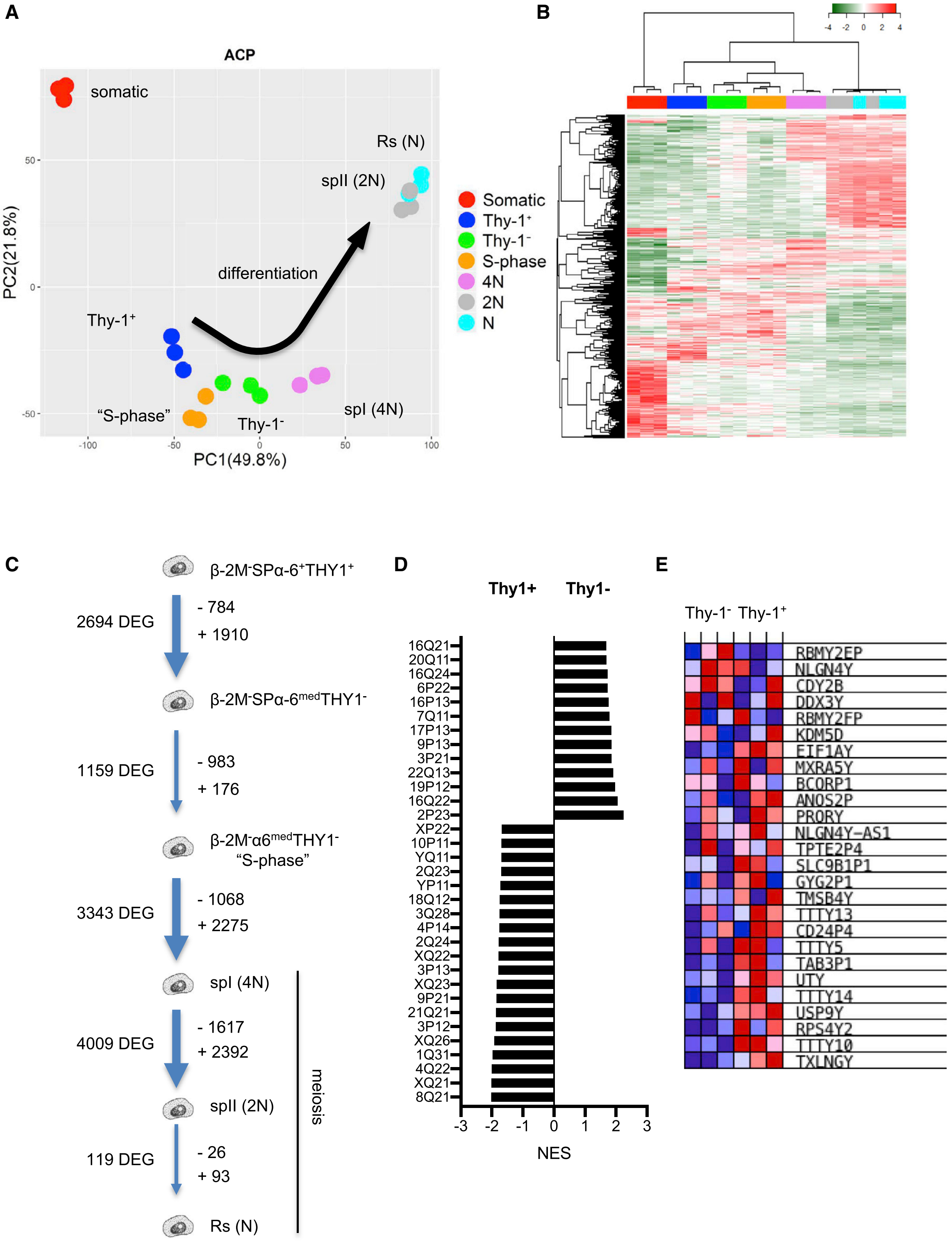

Figure 3. Differential expression of genes during human spermatogenesis

(A) PCA of differentially expressed genes in human germinal populations throughout spermatogenesis and control somatic tissue (PC1–PC2 contributed to intersample variation, as shown in parentheses).

(B) Heatmap of the gene expression of the differentiation stages during spermatogenesis and associated hierarchical clustering.

(legend continued on next page)

the β-2M$^-$SPα-6$^+$ THY1$^+$ population is highly enriched in SSCs.

The transcriptome of human spermatogenesis reveals a specific profile of expression for each differentiation stage

We took advantage of the FACS-based method of purification of human germinal cells to examine the transcriptomic activity of the differentiation stages of human spermatogenesis. The β-2M$^-$SPα-6$^+$THY1$^+$ and β-2M$^-$SPα-6^{med}THY1$^-$ spermatogonial populations were sorted along with an additional subpopulation referred to here as β-2M$^-$α-6^{med}THY1$^-$-"S-phase," with a Vybrant profile exhibiting a continuum between 2N and 4N in DNA content (Figure S1E). We assumed that this population would contain actively replicating S-phase cells destined to differentiate into spermatocyte I cells and should therefore correspond to late-differentiating spermatogonia. The meiotic spermatocyte I and II populations and round spermatids were also purified. A somatic RNA reference sample was prepared from a mixture of CD45$^+$ blood cell RNA, prepuce tissue RNA, and esophageal tissue RNA.

Of the 29,597 gene probes tested, paired comparisons of these populations identified 10,161 genes differentially expressed during the differentiation process ($p < 5 \times 10^{-4}$). A principal-component analysis (PCA) of these genes revealed a clear trend for the transcriptomes to diverge along the germinal differentiation process (Figure 3A). Premeiotic spermatogonial populations clustered together, whereas meiotic and postmeiotic populations diverged, and the somatic transcriptome was clearly distinct from the germinal expression. A heatmap analysis and associated hierarchical clustering of the sorted populations confirmed the sequence of steps during differentiation from the most primitive spermatogonia to haploid spermatids (Figure 3B). Strikingly, the spermatocyte II and round spermatid clusters overlapped, indicating that they have similar gene expression profiles and that developmental determination of haploid cells is initiated at the spermatocyte II stage (Figures 3A and 3B). The dynamics of the germinal transcriptome reveal that transitions between adjacent steps of differentiation were associated with dramatic changes in gene expression during adult human spermatogenesis (Figure 3C).

The transcription status of the sexual chromosomes plays an important role during male germ cell development, especially at meiosis, because of the epigenetic inactivation of meiotic sex chromosomes (MSCI) (Turner, 2007). We observed a dramatic decline of expression of X- and Y-linked genes, as expected, at the cell transition to the spermatocyte I stage (spermatocyte I/β-2M$^-$α-6^{med}THY1$^-$-"S-phase" comparison). The contribution of sexual chromosomes to the transcriptional program decreased, respectively, from 15.4% (165 X-linked genes/1,068 genes) to 0.92% of the transcriptome (21 X-linked genes/2,275 genes) and 0.65% (7 Y-linked genes/1,068 genes) to 0% of the transcriptome (0 Y-linked genes/2,276 genes) for X and Y chromosomes (Table S1; $p < 0.02$, fold change-fc > 2). During transition from spermatocyte I to spermatocyte II, some X- and Y-linked genes continue to be downregulated (64 X-linked genes, 3 X/Y-linked genes, and 1 Y-linked gene), but transcription of other X and Y chromosome genes appeared to be reactivated as soon as the cells reached the spermatocyte II stage (92 X-linked, 3 XY-linked, and 5 Y-linked genes over 2,392 genes upregulated in spermatocyte II), suggesting that X and Y chromosomes were transcriptionally active in postmeiotic spermatids, as shown recently in mice (Margolin et al., 2014).

X-linked genes have also been suggested to be expressed preferentially in premeiotic spermatogonia in spermatogenesis (Wang et al., 2001). Higher numbers of X- and Y-linked genes were expressed in the β-2M$^-$SPα-6$^+$THY1$^+$ transcriptome compared with β-2M$^-$SPα-6^{med}THY1$^-$ at the transition of immature spermatogonia to differentiated spermatogonia: genes in Xq21, Xq22, Xp22, Xq23, Xq26, Yp11, and Yq11 chromosomal regions (Figures 3D and 3E). Interestingly, the azoospermia factor (AZF) locus is located in the Yq11 region, which contains three regions, AZFa (*UTY* and *USP9Y* genes), AZFb (*RPS4Y2* and *TTTY14*, *TTTY5*, *TTTY13*, and *TTTY10* genes), and AZFc, in which microdeletions are associated with fertility issues (Vogt et al., 2017).

Expression profiles of primitive spermatogonial populations enriched in SSCs

Gene lists were extracted from paired comparisons of cells in adjacent stages of differentiation to display the transcriptomic signatures of the different steps of spermatogenesis. We focused our analysis on the β-2M$^-$SPα-6$^+$THY1$^+$ population of immature spermatogonia that contains SSCs. The transcriptomics profiles of populations of differentiating spermatogonia, meiotic and postmeiotic populations, are provided in Figures S3–S5 and Table S2. We identified 784 genes (fc > 2, $p < 0.02$) with higher expression in the β-2M$^-$SPα-6$^+$THY1$^+$ population than in the downstream

(C) Schematic recapitulating the number of differentially expressed genes (DEGs) at transitions between the different differentiation stages.
(D) GSEA of the expression profile signature at the transition of β-2M$^-$SPα-6$^+$THY1$^+$immature spermatogonia to more differentiated spermatogonia according to the chromosomal position of the genes (FDR < 0.03).
(E) Heatmap of DEGs found in the Yq11 region.

A

Top 25 upregulated genes

Symbol	FC	p_value
CXCL9	57.91	8.06E-09
LOC105378011	48.78	3.40E-06
SUMO1	48.47	9.86E-06
TMEM171	33.85	6.66E-10
LOC100506563	31.40	8.05E-07
LOC105375180	26.67	4.44E-07
L1TD1	22.75	4.54E-10
LOC105370287	21.54	6.57E-08
CAMTA1-DT	21.52	2.46E-06
LINC01030	21.29	1.65E-07
MLLT3	19.30	2.19E-08
SMARCA1	16.78	1.05E-06
TMSB15A	15.13	4.07E-05
NUDT11	15.06	1.16E-06
LOC105370115	14.51	2.03E-06
IFI16	14.00	2.65E-09
LOC100287072	13.74	3.81E-05
ARRDC4	13.23	1.73E-06
PIWIL4	13.14	4.10E-05
LOC101928046	13.12	1.67E-04
AK5	12.83	1.96E-07
SERPINI1	12.40	5.51E-09
CCDC160	11.85	3.45E-06
NUTM2B-AS1	11.83	1.40E-04
CERS6	10.97	1.20E-06

B

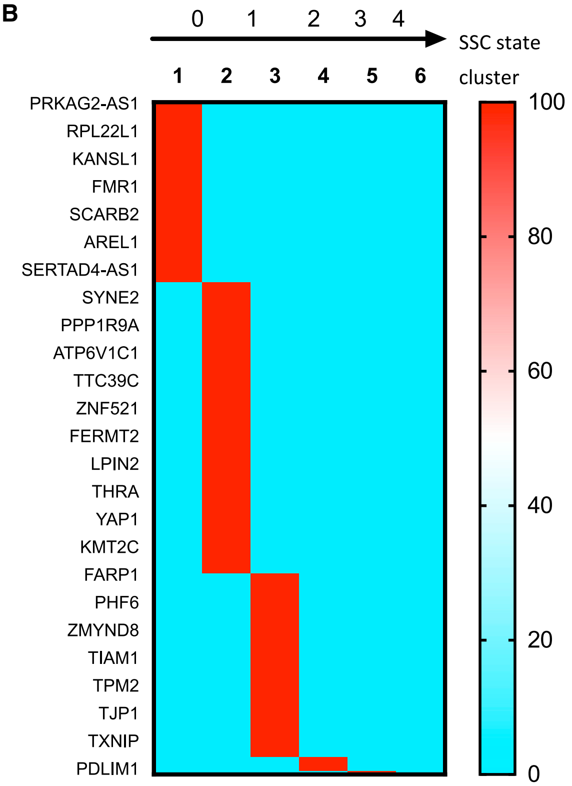

C

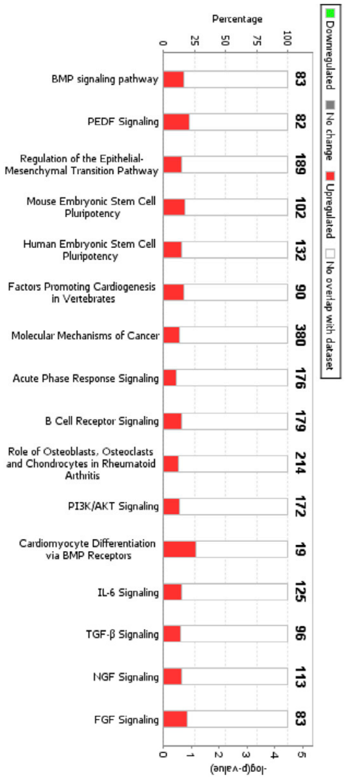

D

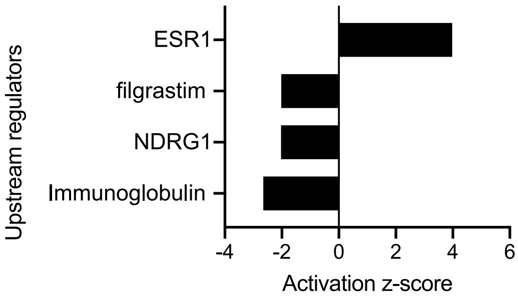

(legend on next page)

progeny, the β-2M$^-$SPα-6^{med}THY1$^-$ population (Table S2). Several of these genes have been reported previously to be relevant in murine SSCs and spermatogonial progenitors. These include *PIWIL4*, *FGFR2*, *GFRA1*, *ADGRA3/GPR125*, *PIK3CA*, *SALL1*, *SALL3*, *TEC*, *ZBTB33*, and *ABCG2/BCRP1*. The top 25 genes are listed in Figure 4A, and the expression of some of these was confirmed by qRT-PCR (Figure S6A). Using an scRNA-seq approach, Guo et al. (2018) identified six gene clusters defining states 0–4 in human adult SSC development. When the β-2M$^-$SPα-6$^+$THY1$^+$ gene list was compared with genes expressed in these six clusters, we found 194 genes common to both lists, of which 97.4% (189 of 194) were distributed in clusters 1, 2, and 3, corresponding to states 0 and 1 in SSC development (Figure 4B). Similar results were obtained when the β-2M$^-$SPα-6$^+$THY1$^+$ gene list was compared with another single-cell transcriptomic analysis of human SSCs (Sohni et al., 2019); 113 common genes were found, of which 71.7% (81 of 113) were distributed in clusters 1A, 1B, 1C, and 2 describing the SSC population (Figure S6B). Compared with the transcriptome of the SSC highly enriched PLPPR3$^+$ population (Tan et al., 2020), we observed that 29% of genes (228 of 784) of the β-2M$^-$ SPα-6$^+$THY1$^+$ population was shared with the PLPPR3$^+$ population, whereas only 11.3% (216 of 1,910) of the transcriptome of PLPPR3$^+$ cells was found in the β-2M-SPα-6^{med}THY1$^-$ population (Figure S6C). This suggested that the β-2M$^-$SPα-6$^+$THY1$^+$ population contained the primitive states of SSC development.

Molecular function and cellular component annotations of the β-2M$^-$SPα-6$^+$ THY1$^+$ gene list according to PANTHER Gene Ontology classification showed that the top biological processes are involved in control of cell movement (motility and cell migration) and in transcriptional regulation of gene expression (Tables S3 and S4). Using Ingenuity Pathway Analysis (IPA), we found that the bone morphogenetic protein pathway, pigment epithelium-derived factor (PEDF) pathway, epithelial-mesenchymal transition (EMT) pathway, and embryonic stem cell pluripotency pathway were among the most highly regulated canonical pathways in the β-2M$^-$SPα-6$^+$THY1$^+$ population (–log p > 3; Figure 4C). In addition, nuclear hormone receptor estrogen receptor ESR1, N-myc downstream-regulated gene 1 (NDRG1), and granulocyte-colony-stimulating factor (G-CSF; filgrastim) signaling were predicted to be potential top upstream regulators of the physiology of primitive spermatogonia (Figure 4D).

Next we focused on transcriptional regulators (TRs) because they play crucial roles in differentiation. Among the 492 TRs that varied across the stages of spermatogenesis (fc > 2, p < 0.02; Table S2), we observed 3 core groups (Figure 5A). The first core was expressed in immature spermatogonia and partially overlapped with the set of TRs expressed in somatic tissues and differentiating spermatogonia. The second core seemed to be activated when spermatogonia became committed to differentiate up to meiosis. The third core began to activate at the onset of meiosis and was fully expressed in later stages. We identified 109 TRs that were preferentially enriched in the β-2M$^-$SPα-6$^+$THY1$^+$ population of immature spermatogonia (Figure 5B; Table S2), including *TAF4B*, *SALL1*, *PRDM1*, and *PRDM14*, known to play a role in germinal lineage development. Enrichment analysis using PANTHER highlighted a role of RUNX and transforming growth factor (TGF)/bone morphogenetic protein (BMP)/ SMAD pathways (Figure 5C), which are involved in stem cell biology (Mevel et al., 2019; Mullen and Wrana, 2017). Interestingly, String analysis identified a network of interactions between 35 of these TRs involved in the regulation of the β-2M$^-$SPα-6$^+$THY1$^+$ population (Figure 5D).

We analyzed the cell state transition between the β-2M$^-$ SPα-6$^+$THY1$^+$ and β-2M$^-$ SPα-6^{med} THY1$^-$ populations by performing gene set enrichment analysis (GSEA). The genes involved in the inflammatory transcriptional program were enriched in β-2M$^-$ SPα-6$^+$THY1$^+$ cells (Table S5; Figure 5E; false discovery rate [FDR] < 0.1). Of note, GSEA also suggests also a role for RAS, transforming growth factor β (TGF-β), Hedgehog, Notch, and the EMT pathway in regulation of differentiation of primitive spermatogonia. Transition from the β-2M$^-$ SPα-6$^+$THY1$^+$ state to the β-2M$^-$ SPα-6^{med}THY1$^-$ state seems to be elicited by genes involved in the MYC, E2F, or unfolded protein response pathway (Table S6). This cell state transition appears to be supported by a switch from hypoxic to oxidative phosphorylation metabolism (Figure 5F).

Comparative transcriptomics analysis of populations of primitive spermatogonia in humans and mice

As shown previously (Barroca et al., 2009; Corbineau et al., 2017), spermatogonial progenitors were discriminated by flow cytometry in mice as follows: β-2M$^-$SPα-6$^+$c-kit$^-$ cells correspond to undifferentiated spermatogonia, including SSCs, and β-2M$^-$SPα-6$^+$c-kit$^+$ cells correspond to differentiating spermatogonia (Figure 6A). Transcriptomics analysis

Figure 4. Expression signature of the primitive spermatogonial T$^+$ population enriched in SSCs
(A) List of the top 25 DEGs.
(B) Heatmap showing the distribution of the expression of genes of the T$^+$ population according to the six gene clusters defining the states 0–4 in human adult SSC development as defined by Guo et al. (2018).
(C) List of the top canonical pathways identified via an Ingenuity Pathway Analysis (IPA).
(D) Putative upstream regulators identified using IPA.

A

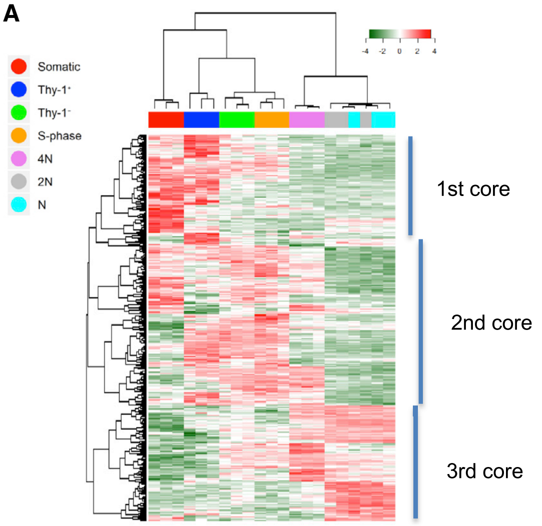

B

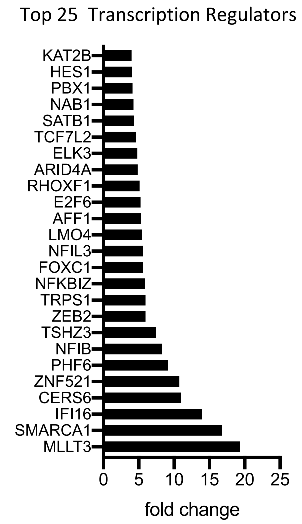

C

REACTOME PATHWAYS	FOLD	FDR
LOSS OF FUNCTION OF SMAD4 IN CANCER (R-HSA-3304347)	> 100	3.20E-02
SMAD2/3 MH2 DOMAIN MUTANTS IN CANCER (R-HSA-3315487)	> 100	3.04E-02
SMAD4 MH2 DOMAIN MUTANTS IN CANCER (R-HSA-3311021)	> 100	2.91E-02
RUNX3 REGULATES BCL2L11 (BIM) TRANSCRIPTION (R-HSA-8952158)	> 100	2.07E-03
RUNX3 REGULATES CDKN1A TRANSCRIPTION (R-HSA-8941855)	79.79	3.05E-03
TRANSCRIPTIONAL REGULATION OF PLURIPOTENT STEM CELLS (R-HSA-452723)	42.31	1.11E-04
RUNX3 REGULATES NOTCH SIGNALING (R-HSA-8941856)	39.89	1.46E-02
NOTCH4 INTRACELLULAR DOMAIN REGULATES TRANSCRIPTION (R-HSA-9013695)	37.23	2.16E-03
RUNX2 REGULATES BONE DEVELOPMENT (R-HSA-8941326)	37.23	1.98E-05

E

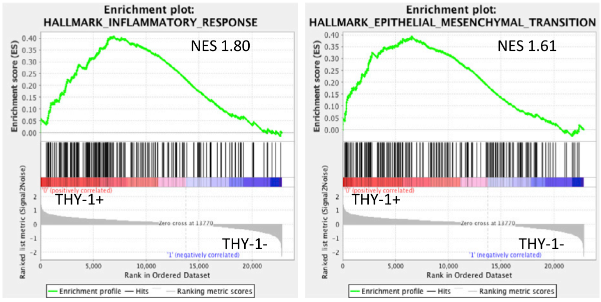

D

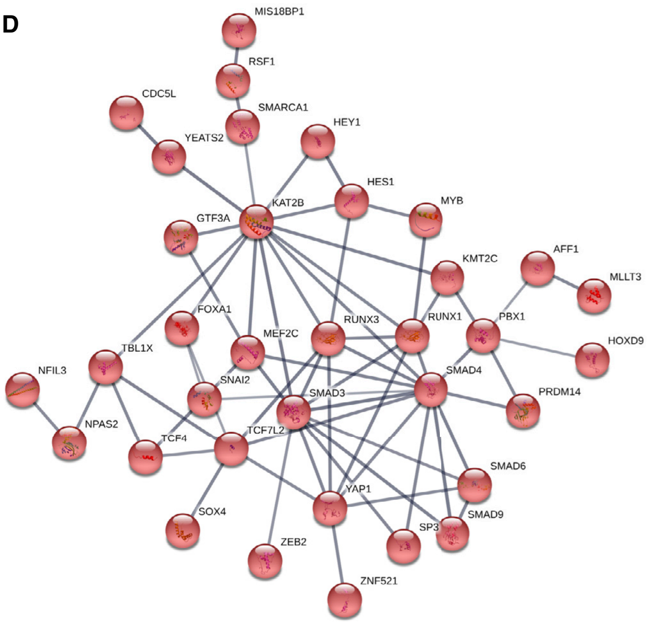

F

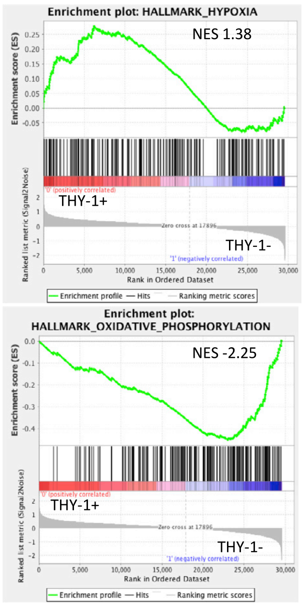

(legend on next page)

was performed on sorted populations of β-2M⁻SPα-6⁺c-kit⁻ and β-2M⁻SPα-6⁺c-kit⁺ spermatogonia (Figure 6B). 1,238 and 913 genes, respectively, were differentially expressed between these two populations (fc > 1.5, $p < 0.02$; Table S7). Genes reported previously to be relevant for undifferentiated spermatogonia were expressed positively in the transcriptomics profile of the β-2M⁻SPα-6⁺c-kit⁻ population, including *Ret*, *Gfra1*, *Etv5*, *Nanos2*, *Nanos3*, *Glis3*, *Sall1*, *Sall4*, *Cdh1*, *Bcl6b*, *Bnc2*, *CD24a*, *Ddit4*, *Ngn3*, *Tec*, *zbtb16*, *Eomes*, *T*, *Nefm*, *Lhx1*, *Smad6*, *TcL1*, *Id4*, *CD9*, *Tspan8*, *Itga6*, *PiwiL4*, *Utf1*, and *Stat3*. We compared the lists of genes enriched in the most primitive spermatogonial population in humans and mice and found that 134 genes were conserved between the human β-2M⁻SPα6⁺THY1⁺ and murine β-2M⁻SPα-6⁺c-kit⁻cell populations (fc > 1.5, $p < 0.02$ for both lists; Figure 6C; Table S7), including *Piwil4*, *Gfra1*, *Pik3ca*, *Sall1*, *Txnip*, *Tec*, and *Zbtb16*, implicated in SSCs and spermatogonial progenitors. Some genes, such as *Chd1*, *Foxo4*, *Pbx1*, *Tcf4*, and *Hes1*, were shown to play roles in the physiology of embryonic or adult stem cells. String analysis identified a network of 76 interacting genes in this set (Figure 6D).

The transcription factor *HES1* plays a role in maintenance of murine SSCs *in vitro*

To validate the transcriptomics profile of the human β-2M⁻SP⁺α-6⁺THY1⁺ population, we focused on the basic-helix-loop-helix (bHLH) transcriptional repressor HES1, which is involved in development of hematopoietic, neural, and intestinal stem cells (Liu et al., 2015). *Hes1* was also conserved in the transcriptome of murine SPβ-2M⁻α-6⁺c-kit⁻ undifferentiated spermatogonia. We confirmed the highest level of expression of *HES1* mRNA in the β-2M⁻SP⁺α-6⁺THY1⁺ population by qRT-PCR (Figure 7A). The HES1 protein was detected in human cells that contacted the basement membrane (Figure 7B) and particularly in MAGEA4⁺ spermatogonia (Figure 7C). We also observed HES1 in GATA-4⁺ Sertoli cells (Figure S6D). We used culture of murine SSCs, which is the best-characterized mammalian model, to study the role of HES1. *Hes1* was silenced in murine adult SSC culture after transfection with small interfering RNA (siRNA), and SSC functionality was assessed 7 days later, as described previously (Oatley et al., 2010), by *in vitro* colony formation ability tests and transplantation assays (Yeh et al., 2007). *Hes1* mRNA was significantly downregulated 48 h after siRNA transfection (Figure 7D). Seven days after transfection, the total numbers of germ cells (Figure 7E) and of cells able to form germinal cell clusters *in vitro* (Figure 7F) were lower, suggesting alteration of SSC maintenance when *Hes1* expression was reduced. To confirm this effect, EGFP-expressing cells 7 days after transfection were transplanted into the seminiferous tubules of γ-irradiated, germ-cell-depleted testes. Ten weeks after transplantation, *Hes1* siRNA-treated donor cells had colonized the recipient testes and were producing normal spermatogenesis (Figure 7G). However, their colony formation activity was lower than that observed in cells treated with a control siRNA, indicating that the SSC content of the cultures was reduced after *Hes1* silencing (Figure 7H). Because HES1 is involved in regulation of quiescence under serum starvation conditions in several cell types (Sang et al., 2008), we tested the effects of enforced expression of HES1 on SSCs poised for quiescence. Using growth factor and serum deprivation conditions (minimal conditions), we induced quiescence in cultured SSCs, as shown by the increase in Ki-67⁻ cells (Figure S6E). SSCs were transduced with lentiviral vectors expressing human HES1, ΔBHES1, or a control (GFP only) construct, with ΔBHES1 representing an HES1 mutant defective in its DNA-binding function (Yu et al., 2006), and GFP-positive cells were sorted by flow cytometry (Figure S6F). As expected, after 96 h under minimal conditions (i.e., without factors), we observed that there were fewer cells in GFP control cultures than in cultures grown in medium supplemented with serum and growth factors (i.e., complete medium) (Figure 7I). However, the number of cells was higher in HES1 expression-enforced cell cultures than in GFP control cell cultures grown under minimal culture conditions. No effects were observed in cultures of enforced mutant ΔBHES1-expressing cells. The frequencies of the numbers of cells with the ability to form germinal colonies *in vitro* remained unchanged in enforced HES1-expressing cell cultures after 96 h of growth under minimal conditions (Figure 7J). Cell death was observed in SSCs cultured under minimal conditions (Figure 7K), as reported previously in other cell types (Jeffers et al., 2003). Strikingly, we observed that the level of cell death was lower when HES1 expression was enforced and returned to the level observed in control cultures grown in complete medium. Inducing ectopic constitutive expression of human *HES1* in murine SSCs and progenitors seemed to protect the cells against cell death when grown under medium starvation conditions. These data indicate that HES1 plays a role in maintenance of SSCs cultured *in vitro*.

Figure 5. Identification of TRs enriched in the primitive spermatogonial T⁺ population
(A) Heatmap of TRs expressed during spermatogenesis and associated hierarchical clustering.
(B) List of the top 25 differentially expressed TRs in the T⁺ population. (C) PANTHER enrichment analysis of the T⁺ set of TRs.
(D) Network of 35 TRs identified using String analysis.
(E and F) GSEA of the cell state transition between the T⁺ and T⁻ spermatogonial populations

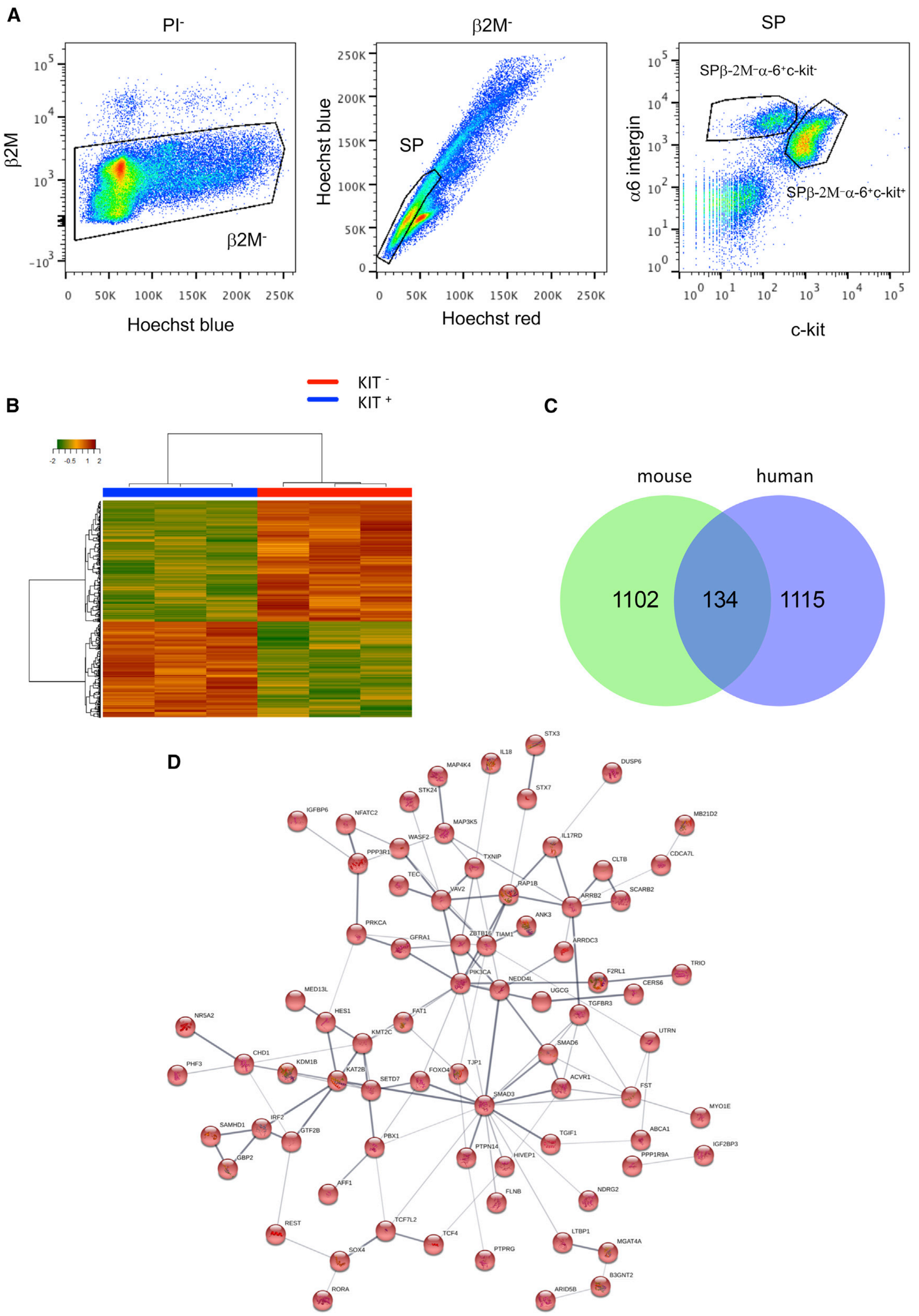

(legend on next page)

DISCUSSION

In humans, identification of SSCs is still limited because of a notable lack of markers. Human A_{dark} and A_{pale} spermatogonia show highly similar transcriptomics profiles (Jan et al., 2017), suggesting that morphological nuclear criteria are not necessarily related to progenitor and stem cell states. Different cell states have been delineated in the primitive spermatogonial and SSC population using scRNA-seq (Wang et al., 2018; Guo et al., 2018; Hermann et al., 2018; Sohni et al., 2019; Shami et al., 2020). However, these putative SSC subsets must be validated by functional assays. TSPAN33 and PLPPR3, identified in these subsets, have been confirmed recently as SSC markers using testicular transplantation assays (Tan et al., 2020; Shami et al., 2020). We designed a combination panel of four SSC markers, described previously in mice (Barroca et al., 2009; Corbineau et al., 2017), that allowed us to identify an SSC population highly enriched with the β-2M$^-$SPα-6$^+$THY1$^+$ phenotype, which was validated using transplantation functional assays.

Some of the markers used in our study, such as ITGA6 and THY1, have been reported previously to be human SSC markers, but they were tested individually and not in combination with other markers (Valli et al., 2014). We show that ABCG2 is involved in the DNA dye efflux that results in the human SP phenotype, similar to what has been observed in mice (Lassalle et al., 2004). A relatively high degree of conservation was observed between rodent and primate SSC markers, although the SP phenotype is still under debate, probably because of the different mouse models and experimental procedures and/or different gating used to define it (Kubota et al., 2003). Compared with other mammalian models, a caveat regarding stem cell identification in humans is the issue of lineage tracing analysis, and analysis of the regenerative capacity after testicular transplantation is still the best functional assay to test the stem cell potential. A testicular transplantation assay showed that the β-2M$^-$SPα-6$^+$ THY1$^+$ population is highly enriched in stem cell activity, and *in vitro* assays showed that these cells have the capacity to form short-term germinal clusters. The stem cell activity of this population (187 colonies/10^5 transplanted donor cells) indicates that the combination of the 4 markers used here improved the definition of this stem cell subpopulation compared with previous strategies (Dovey et al., 2013; Valli et al., 2014). Comparison of the transcriptome of the β-2M$^-$SP$^+$α-6$^+$THY1$^+$ population with that of scRNA-seq studies of human spermatogonia (Guo et al., 2018; Sohni et al., 2019) and that of RNA-seq of PLPPR3$^+$ spermatogonia (Tan et al., 2020) suggests that this population contains the primitive states of human spermatogonia.

We identified genes sets and a network of TRs that are preferentially expressed in this population of human undifferentiated spermatogonia highly enriched for SSCs. Some have been reported previously to be relevant for maintenance of SSC and spermatogonial progenitors in mice (e.g., PIWIL4, FGFR2, GFRA1, ADGRA3/GPR125, PIK3CA, SALL1, SALL3, TEC, ZBTB33, and ABCG2/BCRP1). Gene Ontology annotations highlighted cell migration and motility as top biological processes in human β-2M$^-$SP$^+$α-6$^+$THY1$^+$ cells. Migration of SSCs over the seminiferous tubules niche is observed frequently in mice and plays a role in determination of their fate toward self-renewal or differentiation (Nakagawa et al., 2010). TGF/BMP/SMAD, PEDF, EMT, and the embryonic stem cell pluripotency pathways were among the most highly regulated in human primitive spermatogonia. Interestingly, PEDF has been reported to be secreted by human testicular peritubular cells (Windschüttl et al., 2015). EMT is a highly conserved cellular process that involves transformation of epithelial cells into mesenchymal cells and has been implicated in embryogenesis, tissue repair, and tumorigenesis. EMT signaling via stringent attachment of SSCs and spermatogonial progenitors to the basement membrane could play a role in their physiologic status and cell fate determination. NDRG1 and G-CSF signaling, predicted to be potential top upstream regulators of primitive spermatogonia, belong to pathways involved in regulation of murine SSCs (Kanatsu-Shinohara et al., 2016; Kotzur et al., 2017). Inflammatory signaling, known to affect the hematopoietic stem cell fate (Pietras, 2017), has also been found to be relevant to human β-2M$^-$SP$^+$α-6$^+$THY1$^+$ SSCs.

SSC maintenance was affected in culture *in vitro* when *Hes1* expression was reduced. Constitutive downregulation of *Hes1* can affect stem cells and can lead to neuronal differentiation (Liu et al., 2015; Kageyama et al., 2008).

Figure 6. Comparative analysis of DEGs in human and mouse populations of adult primitive spermatogonia
(A) Flow cytometry analysis of mouse α-6$^+$ testicular cells selected by magnetic activated cell sorting (MACS) based on blue and red Hoechst fluorescence and the markers β2M, α-6 integrin, and c-kit. The SPβ2M$^-$α-6$^+$c-kit$^-$ and SPβ2M$^-$α-6$^+$c-kit$^+$ populations are indicated. Cells were also selected on FSC parameters to exclude elongated spermatids.
(B) Heatmap of the gene expression in the mouse undifferentiated SPβ-2M$^-$α-6$^+$c-kit$^-$ and differentiated SPβ-2M$^-$α-6$^+$c-kit$^+$ spermatogonial population and associated hierarchical clustering.
(C) Venn diagram showing the relationships between the transcriptomes of human T$^+$ and mouse β-2M$^-$SPα-6$^+$c-kit$^-$ spermatogonial populations. The number of genes in each group is indicated.
(D) Network of 76 interacting genes using String analysis in the conserved set of genes between mouse and human models.

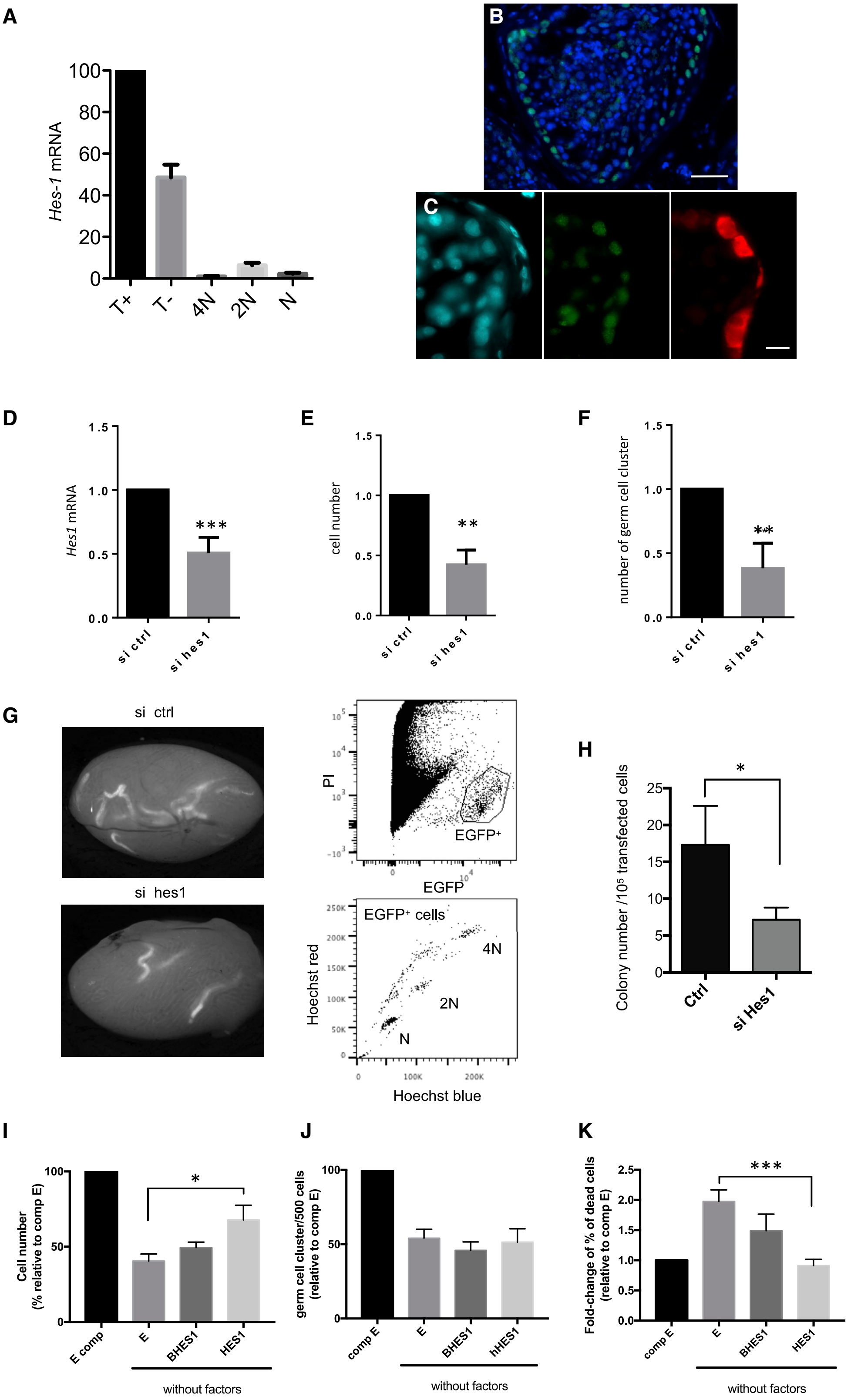

(legend on next page)

Ectopic constitutive expression of human HES1 has a protective effect in murine SSCs and progenitors when they are exposed to medium starvation conditions and poised for quiescence, as seen in other cell types (Sang et al., 2008). Targeted overexpression of HES1 has been shown previously to have a survival effect in melanoblasts by protecting the cells from elimination by apoptosis (Moriyama et al., 2006), in line with our observations on cell death in SSC culture. Hence, the role of HES1 in the SSC physiology, at least *in vitro*, questions the potential role of the NOTCH pathway in SSCs, which remains controversial (Hasegawa et al., 2012; Huang et al., 2013; Garcia et al., 2017). Other factors, such as FGF2, can also induce HES1 expression (Sato et al., 2010). Although the mechanisms involved in the function of HES1 need further investigation, especially by *in vivo* analyses, our results suggest that HES1 plays a role in the physiology of murine SSCs.

Studies using high-throughput RNA profiling to comprehensively analyze gene expression in spermatogenesis will increase our understanding of the molecular pathways involved in germ cell differentiation and promote identification of the molecular defects responsible for infertility. Deciphering the molecular pathways that regulate self-renewal and proliferation in SSCs could also help with developing and improving culture conditions for *in vitro* amplification of human SSCs, which is critically important for applying SSCs in therapeutic treatments.

EXPERIMENTAL PROCEDURES

For details, see the supplemental experimental procedures.

Experimental model and human materials

Adult human testis biopsies were obtained from individuals with obstructive azoospermia with normal spermatogenesis who consented to inclusion in this study (institutional review board [IRB]-approved protocol IRB 00003835, 2012/40ICB).

Flow cytometry analysis of testicular single-cell suspensions

Testicular single-cell suspensions were prepared from human biopsies. Vybrant staining (1 μg/mL) and immunolabeling of α-6 integrin, THY1, and β2M of the cell suspensions were performed as described previously (Barroca et al., 2009; Corbineau et al., 2017). This protocol was applied to 16 human donors in this study, providing representative flow cytometry profiles as illustrated in Figure 1A.

Transcriptome

RNA samples were analyzed using an Affymetrix Human Gene 2.1 ST Array and an Affymetrix GeneChip Mouse Gene 2.0 ST Array (Thermo Fisher Scientific).

Statistics

All values are shown as mean ± SEM. Statistical analyses were performed by Student's t test (GraphPad Prism software): ns, not significant, $p > 0.05$; *$p < 0.05$; **$p < 0.01$; ***$p < 0.001$.

Data and code availability

The accession number for the microarray data reported in this paper is GEO: GSE155509.

SUPPLEMENTAL INFORMATION

Supplemental information can be found online at https://doi.org/10.1016/j.stemcr.2022.02.017.

Figure 7. The transcription factor HES1 is involved in maintenance of murine SSCs
(A) Analysis by qRT-PCR of the expression of *HES1* in human T^+, T^-, spermatocyte I (4N), spermatocyte II (2N), and spermatid (N) populations.(n = 3 experiments).
(B and C) Immunofluorescent detection of HES1 in human testes.
(B) HES1, green; DAPI, blue; scale bar, 40 μm.
(C) HES1, green; MAGE4, red; DAPI, blue; scale bar, 10 μm.
(D) *Hes1* silencing in transfected SSCs using siRNA (n = 7 transfection experiments).
(E) Analysis of the total number of germ cells (n = 10 colony tests from 7 transfection experiments).
(F) Total number of germ cell clusters formed 7 days after transfection (n = 7 transfection experiments).
(G) Analysis of regenerative spermatogenesis after transplantation of SSCs 7 days after transfection. Shown is detection of EGFP-fluorescent seminiferous tubules on macroscopic observation in recipient testes 2 months after transplantation in control (top) and *Hes1* siRNA-treated (bottom), EGFP-expressing SSCs. Also shown is red/blue Hoechst 33342 fluorescence analysis of $EGFP^+$ cells obtained from a recipient testis transplanted with *Hes1* siRNA-treated, EGFP-expressing SSCs. Meiotic spermatocyte I (2N), spermatocyte II (4N), and postmeiotic (N) cells are indicated.
(H) Colonization of recipient testes was lower in testes transplanted with culture of *Hes1* siRNA-treated SSCs than in those transplanted with control siRNA-treated SSCs 7 days after transfection (n = 8 recipient testes from 6 transfection experiments).
(I–K) The effects of enforced expression of human HES1 in cultures of SSCs exposed to growth factors and serum deprivation conditions (J) on total cell number (n = 4 experiments), frequency of *in vitro* cluster-initiating cells (n = 12 colony tests from 4 experiments), and (K) cell death (n = 4 experiments). HES1, ΔBHES1 (BHES1), and GFP control (E) cells were grown with growth factors under serum deprivation conditions (without factors) or complete medium (E comp).

AUTHOR CONTRIBUTIONS

M.G., V.F., B.L., V.B.-L., and P.F. provided project design. M.G., V.F., B.L., and P.F. performed experiments and analyzed and interpreted data. A.S.G., C.L., I.H., A.J., F.H., I.A., and L.R. participated in experimentation and provided comments. F.D., S.J., and F.L. participated in microarray and bioinformatics analyses. F.P. provided NSG mice. N.T., C.P., J.P.W., and V.B.-L. supervised sample acquisition. M.G., V.F., and P.F. prepared the manuscript.

CONFLICTS OF INTEREST

The authors declare no competing interests.

ACKNOWLEDGMENTS

The authors acknowledge support of the members of the CECOS from CHU Cochin. This work was supported by grants from ANR, the Agence de Biomédecine, CEA IRBIO, EDF, and the Fondation ARC pour la recherche sur le cancer.

Received: December 11, 2020
Revised: February 25, 2022
Accepted: February 25, 2022
Published: March 24, 2022

REFERENCES

Allen, J.D., van Loevezijn, A., Lakhai, J.M., van der Valk, M., van Tellingen, O., Reid, G., Schellens, J.H., Koomen, G.J., and Schinkel, A.H. (2002). Potent and specific inhibition of the breast cancer resistance protein multidrug transporter in vitro and in mouse intestine by a novel analogue of fumitremorgin C. Mol. Cancer Ther. *1*, 417–425.

Barroca, V., Lassalle, B., Coureuil, M., Louis, J.P., Le Page, F., Testart, J., Allemand, I., Riou, L., and Fouchet, P. (2009). Mouse differentiating spermatogonia can generate germinal stem cells in vivo. Nat. Cell Biol. *11*, 190–196.

Bissig-Choisat, B., Wang, L., Legras, X., Saha, P.K., Chen, L., Bell, P., Pankowicz, F.P., Hill, M.C., Barzi, M., Leyton, C.K., et al. (2015). Development and rescue of human familial hypercholesterolaemia in a xenograft mouse model. Nat. Commun. *6*, 7339.

Bleau, A.M., Hambardzumyan, D., Ozawa, T., Fomchenko, E.I., Huse, J.T., Brennan, C.W., and Holland, E.C. (2009). PTEN/PI3K/Akt pathway regulates the side population phenotype and ABCG2 activity in glioma tumor stem-like cells. Cell Stem Cell *4*, 226–235.

Brinster, R.L. (2007). Male germline stem cells: from mice to men. Science *316*, 404–405.

Corbineau, S., Lassalle, B., Givelet, M., Souissi-Sarahoui, I., Firlej, V., Romeo, P.H., Allemand, I., Riou, L., and Fouchet, P. (2017). Spermatogonial stem cells and progenitors are refractory to reprogramming to pluripotency by the transcription factors *Oct3/4*, c-*Myc*, *Sox2* and *Klf4*. Oncotarget *8*, 10050–10063.

de Rooij, D.G. (2017). The nature and dynamics of spermatogonial stem cells. Development *144*, 3022–3030.

Dovey, S.L., Valli, H., Hermann, B.P., Sukhwani, M., Donohue, J., Castro, C.A., Chu, T., Sanfilippo, J.S., and Orwig, K.E. (2013). Eliminating malignant contamination from therapeutic human spermatogonial stem cells. J. Clin. Invest *123*, 1833–1843.

Ehmcke, J., and Schlatt, S. (2006). A revised model for spermatogonial expansion in man: lessons from non-human primates. Reproduction *132*, 673–680.

Eildermann, K., Gromoll, J., and Behr, R. (2012). Misleading and reliable markers to differentiate between primate testis-derived multipotent stromal cells and spermatogonia in culture. Hum. Reprod. *27*, 1754–1767.

Falciatori, I., Borsellino, G., Haliassos, N., Boitani, C., Corallini, S., Battistini, L., Bernardi, G., Stefanini, M., and Vicini, E. (2004). Identification and enrichment of spermatogonial stem cells displaying side-population phenotype in immature mouse testis. FASEB J. *18*, 376–378.

Garcia, T.X., Parekh, P., Gandhi, P., Sinha, K., and Hofmann, M.C. (2017). The NOTCH ligand JAG1 regulates GDNF expression in Sertoli cells. Stem Cells Dev. *26*, 585–598.

Guo, J., Grow, E.J., Mlcochova, H., Maher, G.J., Lindskog, C., Nie, X., Guo, Y., Takei, Y., Yun, J., Cai, L., et al. (2018). The adult human testis transcriptional cell atlas. Cell Res. *28*, 1141–1157.

Hara, K., Nakagawa, T., Enomoto, H., Suzuki, M., Yamamoto, M., Simons, B.D., and Yoshida, S. (2014). Mouse spermatogenic stem cells continually interconvert between equipotent singly isolated and syncytial states. Cell Stem Cell *14*, 658–672.

Hasegawa, K., Okamura, Y., and Saga, Y. (2012). Notch signaling in Sertoli cells regulates cyclical gene expression of Hes1 but is dispensable for mouse spermatogenesis. Mol. Cell Biol. *32*, 206–215.

Hermann, B.P., Sukhwani, M., Winkler, F., Pascarella, J.N., Peters, K.A., Sheng, Y., Valli, H., Rodriguez, M., Ezzelarab, M., Dargo, G., et al. (2012). Spermatogonial stem cell transplantation into rhesus testes regenerates spermatogenesis producing functional sperm. Cell Stem Cell *11*, 715–726.

Hermann, B.P., Cheng, K., Singh, A., Roa-De La Cruz, L., Mutoji, K.N., Chen, I.C., Gildersleeve, H., Lehle, J.D., Mayo, M., Westernströer, B., et al. (2018). The mammalian spermatogenesis single-cell transcriptome, from spermatogonial stem cells to spermatids. Cell Rep. *25*, 1650–1667.e8.

Huang, Z., Rivas, B., and Agoulnik, A.I. (2013). NOTCH1 gain of function in germ cells causes failure of spermatogenesis in male mice. PLoS One *8*, e71213.

Jan, S.Z., Vormer, T.L., Jongejan, A., Röling, M.D., Silber, S.J., de Rooij, D.G., Hamer, G., Repping, S., and van Pelt, A.M.M. (2017). Unraveling transcriptome dynamics in human spermatogenesis. Development *144*, 3659–3673.

Jeffers, J.R., Parganas, E., Lee, Y., Yang, C., Wang, J., Brennan, J., MacLean, K.H., Han, J., Chittenden, T., Ihle, J.N., et al. (2003). Puma is an essential mediator of p53-dependent and -independent apoptotic pathways. Cancer Cell *4*, 321–328.

Kageyama, R., Ohtsuka, T., and Kobayashi, T. (2008). Roles of Hes genes in neural development. Dev. Growth Differ. *50*, S97–S103.

Kanatsu-Shinohara, M., Tanaka, T., Ogonuki, N., Ogura, A., Morimoto, H., Cheng, P.F., Eisenman, R.N., Trumpp, A., and Shinohara, T. (2016). Myc/Mycn-mediated glycolysis enhances mouse spermatogonial stem cell self-renewal. Genes Dev. *30*, 2637–2648.

Kotzur, T., Benavides-Garcia, R., Mecklenburg, J., Sanchez, J.R., Reilly, M., and Hermann, B.P. (2017). Granulocyte colony-stimulating factor (G-CSF) promotes spermatogenic regeneration from surviving spermatogonia after high-dose alkylating chemotherapy. Reprod. Biol. Endocrinol. *15*, 7.

Kubota, H., Avarbock, M.R., and Brinster, R.L. (2003). Spermatogonial stem cells share some, but not all, phenotypic and functional characteristics with other stem cells. Proc. Natl. Acad. Sci. U S A *100*, 6487–6492.

Lassalle, B., Bastos, H., Louis, J.P., Riou, L., Testart, J., Dutrillaux, B., Fouchet, P., and Allemand, I. (2004). Side Population' cells in adult mouse testis express Bcrp1 gene and are enriched in spermatogonia and germinal stem cells. Development *131*, 479–487.

Liu, Z.H., Dai, X.M., and Du, B. (2015). Hes1: a key role in stemness, metastasis and multidrug resistance. Cancer Biol. Ther. *16*, 353–359.

Margolin, G., Khil, P.P., Kim, J., Bellani, M.A., and Camerini-Otero, R.D. (2014). Integrated transcriptome analysis of mouse spermatogenesis. BMC Genomics *15*, 39.

Medrano, J.V., Rombaut, C., Simon, C., Pellicer, A., and Goossens, E. (2016). Human spermatogonial stem cells display limited proliferation in vitro under mouse spermatogonial stem cell culture conditions. Fertil. Steril *106*, 1539–1549.e8.

Mevel, R., Draper, J.E., Lie-A-Ling, M., Kouskoff, V., and Lacaud, G. (2019). RUNX transcription factors: orchestrators of development. Development *146*, dev148296.

Moriyama, M., Osawa, M., Mak, S.S., Ohtsuka, T., Yamamoto, N., Han, H., Delmas, V., Kageyama, R., Beermann, F., Larue, L., et al. (2006). Notch signaling via Hes1 transcription factor maintains survival of melanoblasts and melanocyte stem cells. J. Cell Biol. *173*, 333–339.

Mullen, A.C., and Wrana, J.L. (2017). TGF-β family signaling in embryonic and somatic stem-cell renewal and differentiation. Cold Spring Harb. Perspect. Biol. *9*, a022186.

Nakagawa, T., Sharma, M., Nabeshima, Y., Braun, R.E., and Yoshida, S. (2010). Functional hierarchy and reversibility within the murine spermatogenic stem cell compartment. Science *328*, 62–67.

Nickkholgh, B., Mizrak, S.C., Korver, C.M., van Daalen, S.K., Meissner, A., Repping, S., and van Pelt, A.M. (2014). Enrichment of spermatogonial stem cells from long-term cultured human testicular cells. Fertil. Steril *102*, 558–565.e5.

Oatley, J.M., Kaucher, A.V., Avarbock, M.R., and Brinster, R.L. (2010). Regulation of mouse spermatogonial stem cell differentiation by STAT3 signaling. Biol. Reprod. *83*, 427–433.

Pietras, E.M. (2017). Inflammation: a key regulator of hematopoietic stem cell fate in health and disease. Blood *130*, 1693–1698.

Sang, L., Coller, H.A., and Roberts, J.M. (2008). Control of the reversibility of cellular quiescence by the transcriptional repressor HES1. Science *321*, 1095–1100.

Sato, T., Shimazaki, T., Naka, H., Fukami, S., Satoh, Y., Okano, H., Lax, I., Schlessinger, J., and Gotoh, N. (2010). FRS2α regulates Erk levels to control a self-renewal target Hes1 and proliferation of FGF-responsive neural stem/progenitor cells. Stem Cells *28*, 1661–1673.

Shami, A.N., Zheng, X., Munyoki, S.K., Ma, Q., Manske, G.L., Green, C.D., Sukhwani, M., Orwig, K.E., Li, J.Z., and Hammoud, S.S. (2020). Single-cell RNA sequencing of human, macaque, and mouse testes uncovers conserved and divergent features of mammalian spermatogenesis. Dev. Cell *54*, 529–547.e12.

Sohni, A., Tan, K., Song, H.W., Burow, D., de Rooij, D.G., Laurent, L., Hsieh, T.C., Rabah, R., Hammoud, S.S., Vicini, E., et al. (2019). The neonatal and adult human testis defined at the single-cell level. Cell Rep *26*, 1501–1517.e4.

Tan, K., Song, H.W., Thompson, M., Munyoki, S., Sukhwani, M., Hsieh, T.C., Orwig, K.E., and Wilkinson, M.F. (2020). Transcriptome profiling reveals signaling conditions dictating human spermatogonia fate in vitro. Proc. Natl. Acad. Sci. U S A *117*, 17832–17841.

Turner, J.M. (2007). Meiotic sex chromosome inactivation. Development *134*, 1823–1831.

Valli, H., Sukhwani, M., Dovey, S.L., Peters, K.A., Donohue, J., Castro, C.A., Chu, T., Marshall, G.R., and Orwig, K.E. (2014). Fluorescence- and magnetic-activated cell sorting strategies to isolate and enrich human spermatogonial stem cells. Fertil. Steril *102*, 566–580.e7.

Vogt, P.H., Bender, U., Zimmer, J., and Strowitzki, T. (2017). Human Y chromosome and male infertility: forward and back from azoospermia factor chromatin structure to azoospermia factor gene function. Genetics of human infertility. Monogr. Hum. Genet. *21*, 57–73.

Wang, M., Liu, X., Chang, G., Chen, Y., An, G., Yan, L., Gao, S., Xu, Y., Cui, Y., Dong, J., et al. (2018). Single-cell RNA sequencing analysis reveals sequential cell fate transition during human spermatogenesis. Cell Stem Cell *23*, 599–614.e4.

Wang, P.J., McCarrey, J.R., Yang, F., and Page, D.C. (2001). An abundance of X-linked genes expressed in spermatogonia. Nat. Genet. *27*, 422–426.

Windschüttl, S., Kampfer, C., Mayer, C., Flenkenthaler, F., Fröhlich, T., Schwarzer, J.U., Köhn, F.M., Urbanski, H., Arnold, G.J., and Mayerhofer, A. (2015). Human testicular peritubular cells secrete pigment epithelium-derived factor (PEDF), which may be responsible for the avascularity of the seminiferous tubules. Sci. Rep. *5*, 12820.

Yeh, J.R., Zhang, X., and Nagano, M.C. (2007). Establishment of a short-term in vitro assay for mouse spermatogonial stem cells. Biol. Reprod. *77*, 897–904.

Yu, X., Alder, J.K., Chun, J.H., Friedman, A.D., Heimfeld, S., Cheng, L., and Civin, C.I. (2006). HES1 inhibits cycling of hematopoietic progenitor cells via DNA binding. Stem Cells *24*, 876–888.

Zhou, S., Schuetz, J.D., Bunting, K.D., Colapietro, A.M., Sampath, J., Morris, J.J., Lagutina, I., Grosveld, G.C., Osawa, M., Nakauchi, H., et al. (2001). The ABC transporter Bcrp1/ABCG2 is expressed in a wide variety of stem cells and is a molecular determinant of the side-population phenotype. Nat. Med. *7*, 1028–1034.

Zohni, K., Zhang, X., Tan, S.L., Chan, P., and Nagano, M. (2012). CD9 is expressed on human male germ cells that have a long-term repopulation potential after transplantation into mouse testes. Biol. Reprod. *87*, 27.

Stem Cell Reports, Volume *17*

Supplemental Information

Transcriptional profiling of β-2M^{-}SPα-6^{+}THY1^{+} spermatogonial stem cells in human spermatogenesis

Maelle Givelet, Virginie Firlej, Bruno Lassalle, Anne Sophie Gille, Clementine Lapoujade, Isabelle Holtzman, Amandine Jarysta, Farahd Haghighirad, Florent Dumont, Sébastien Jacques, Franck Letourneur, Françoise Pflumio, Isabelle Allemand, Catherine Patrat, Nicolas Thiounn, Jean Philippe Wolf, Lydia Riou, Virginie Barraud-Lange, and Pierre Fouchet

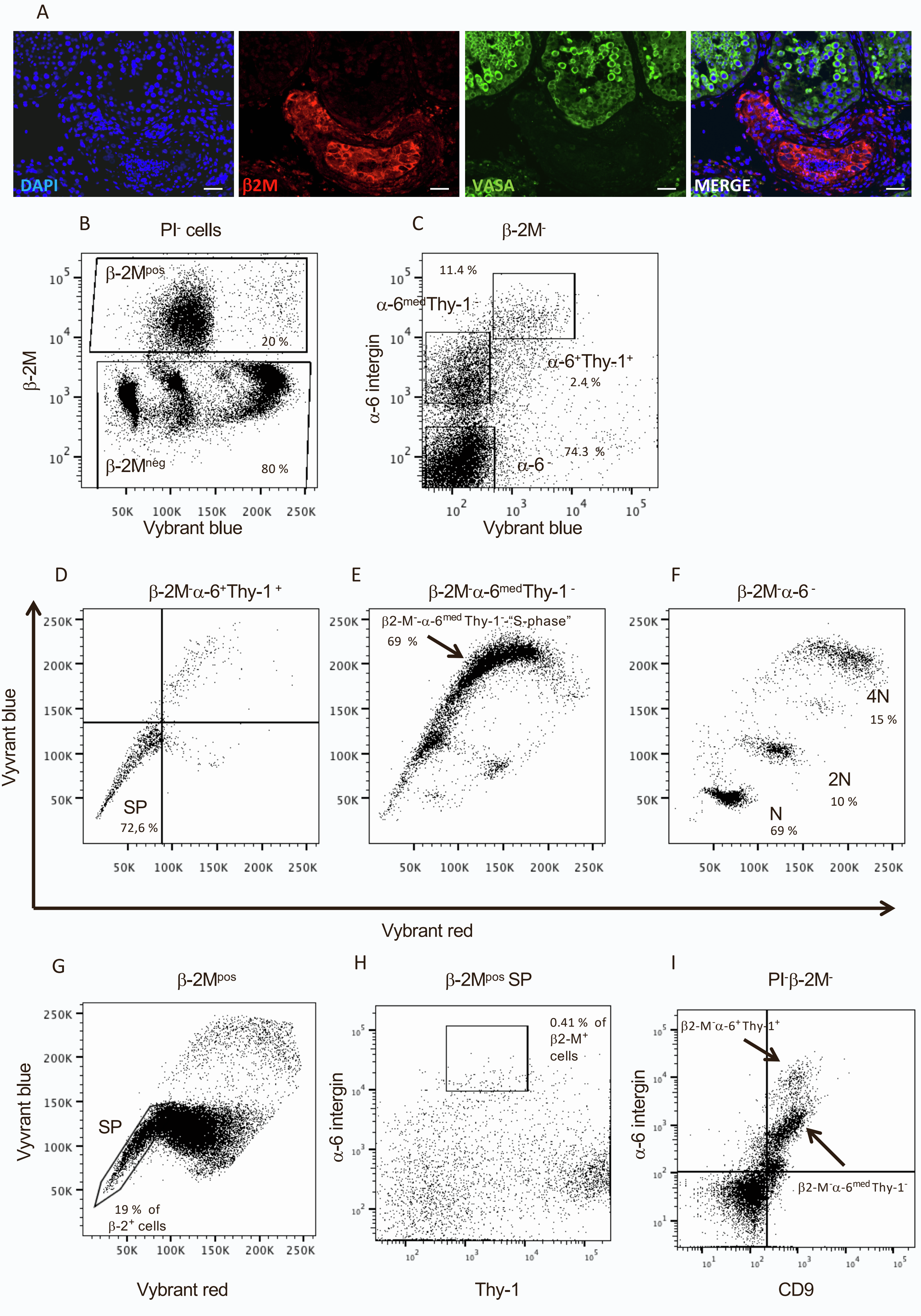
A
DAPI
β2M
VASA
MERGE
B
PI- cells
β-2Mpos
20 %
β-2Mnég
80 %
β-2M
Vybrant blue
C
β-2M-
11.4 %
α-6medThy-1-
α-6+Thy-1+
2.4 %
74.3 %
α-6-
α-6 intergin
Vybrant blue
D
β-2M-α-6+Thy-1+
SP
72,6 %
E
β-2M-α-6medThy-1-
β2-M--α-6med Thy-1-"S-phase"
69 %
F
β-2M-α-6-
4N
15 %
2N
10 %
N
69 %
Vyvrant blue
Vybrant red
G
β-2Mpos
SP
19 % of
β-2+ cells
Vyvrant blue
Vybrant red
H
β-2Mpos SP
0.41 % of
β2-M+
cells
α-6 intergin
Thy-1
I
PI-β-2M-
β2-M-α-6+Thy-1+
β2-M-α-6med Thy-1-
α-6 intergin
CD9

Figure S1

Supplemental Figure S1: β2M^{-} α-6^{+}THY1^{+} cells are mainly found in the SP population. (A) Immunofluorescent analysis of β2M expression in human testicular tissue. β-2M fluorescent signal was observed in the interstitial tissue, but not in DDX4-positive germ cells, scale bar: 20 microns. (B) β2M expression and Vybrant blue fluorescence in viable human testicular cells (PI-negative). (C) THY1 and α-6 integrin expression in β2M-negative cells. β2M^{-}α-6^{+}THY1^{+}, β2M^{-}SPα-6^{med}THY1^{-}, and α-6^{neg} cells are indicated. (D) (E) (F) Vybrant blue and red fluorescence in β2M^{-}α-6^{+}THY1^{+} (D), β2M^{-} α-6^{med}THY1^{-} (E), and α-6^{neg} populations (F) as defined in Fig. S1C. Side population (SP); meiotic and postmeiotic subpopulations of spermatocyte I (4N DNA content), spermatocyte II (2N DNA content), and spermatid (N DNA content) cells, and "β2M^{-}-α-6^{med}THY1^{-}-"S-phase" cells are indicated. (G) Vybrant blue and red fluorescence in β2M-positive testicular cells as defined in Fig. S1B. (H) THY1 and α-6 integrin expression in β2M^{+}SP cells. Low frequency of somatic SPα-6^{+}THY1^{+} cells were also detected in the β-2M+ population (I) CD9 and α-6 integrin expression in viable PI^{-}β2M^{-} cells. Cells corresponding to β2M^{-}α-6^{+}THY1^{+} and β2M^{-}α-6^{med}THY1^{-} populations are indicated.

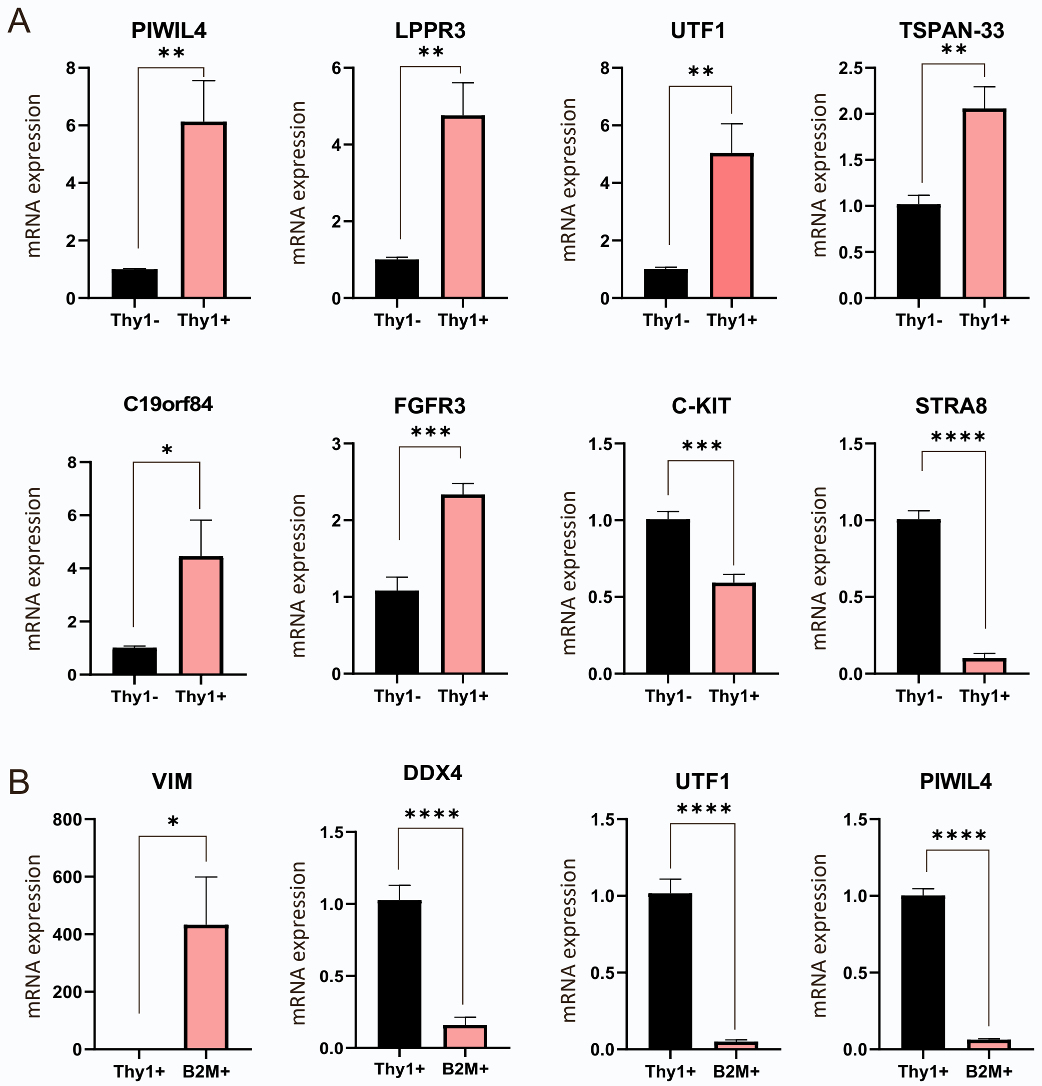
A
PIWIL4
LPPR3
UTF1
TSPAN-33
C19orf84
FGFR3
C-KIT
STRA8
B
VIM
DDX4
UTF1
PIWIL4
mRNA expression
Thy1-
Thy1+
B2M+
**
***
****
*

Figure S2

Supplemental Figure S2: (A) Analysis by RT-qPCR of the expression of *PIWIL4* (n=4), *C19orf84* (n=5), *TSPAN33* (n=6), *PLPPR3* (n=5), *FGFR3* (n=5), *UTF1* (n=5), *KIT*(n=5) and *STRA8* (n=5) gene spermatogonial markers in the β-2M$^-$SPα-6$^+$THY1$^+$ (T$^+$) and β-2M$^-$SPα-6^{med}THY1$^-$ (T$^-$) populations (pool of RNA from 3 different donors), (B) Analysis by RT-qPCR of the expression of the somatic *VIM* gene marker (n=5), and of *DDX4/VASA* (n=6), *PIWIL4* (n=4) and *UTF1* (n=5) gene spermatogonial markers in the β-2M$^-$SPα-6$^+$THY1$^+$ (Thy1$^+$) and β-2M$^+$ (B2M$^+$) populations. (pool of RNA from 2 different donors)

A

Symbol	FC
CABS1	51.10
LINC01760	48.08
LOC105376980	39.29
LOC101928317	28.75
LYZL6	25.25
FBXW10	24.39
LOC105374836	21.98
LINC01766	21.11
ACTRT2	21.03
LINC01921	19.86
MCHR2-AS1	19.60
PLAAT5	19.06
LOC107983959	17.83
ACTL7A	17.75
GTSF1L	17.05
FAM209A	15.72
IQCF4	15.58
LINC00919	15.04
CAPZA3	14.84
TMCO2	14.68
CCDC54	14.57
LOC107986582	14.48
HMGB4	14.44
ZC2HC1B	14.21
P3R3URF	14.08

B

Symbol	FC
SNORD13P1	17.63
LINC02116	12.04
FAM197Y9	10.83
MIR4324	10.61
H3-4	9.10
FBXO47	8.33
LINC02460	7.53
HESX1	7.34
UBR5-AS1	7.17
HOTAIR	5.46
GEMIN8P4	5.31
HOXC5	5.07
RAD51AP2	5.06
HLA-B	5.02
RAB41	4.83
OTUD6A	4.78
TNFAIP8L3	4.66
LOC105375605	4.58
LOC100499489	4.57
OR3A2	4.56
SPO11	4.52
USP6	4.43
PRSS41	4.32
PRSS38	4.25
LOC105372210	4.20

C

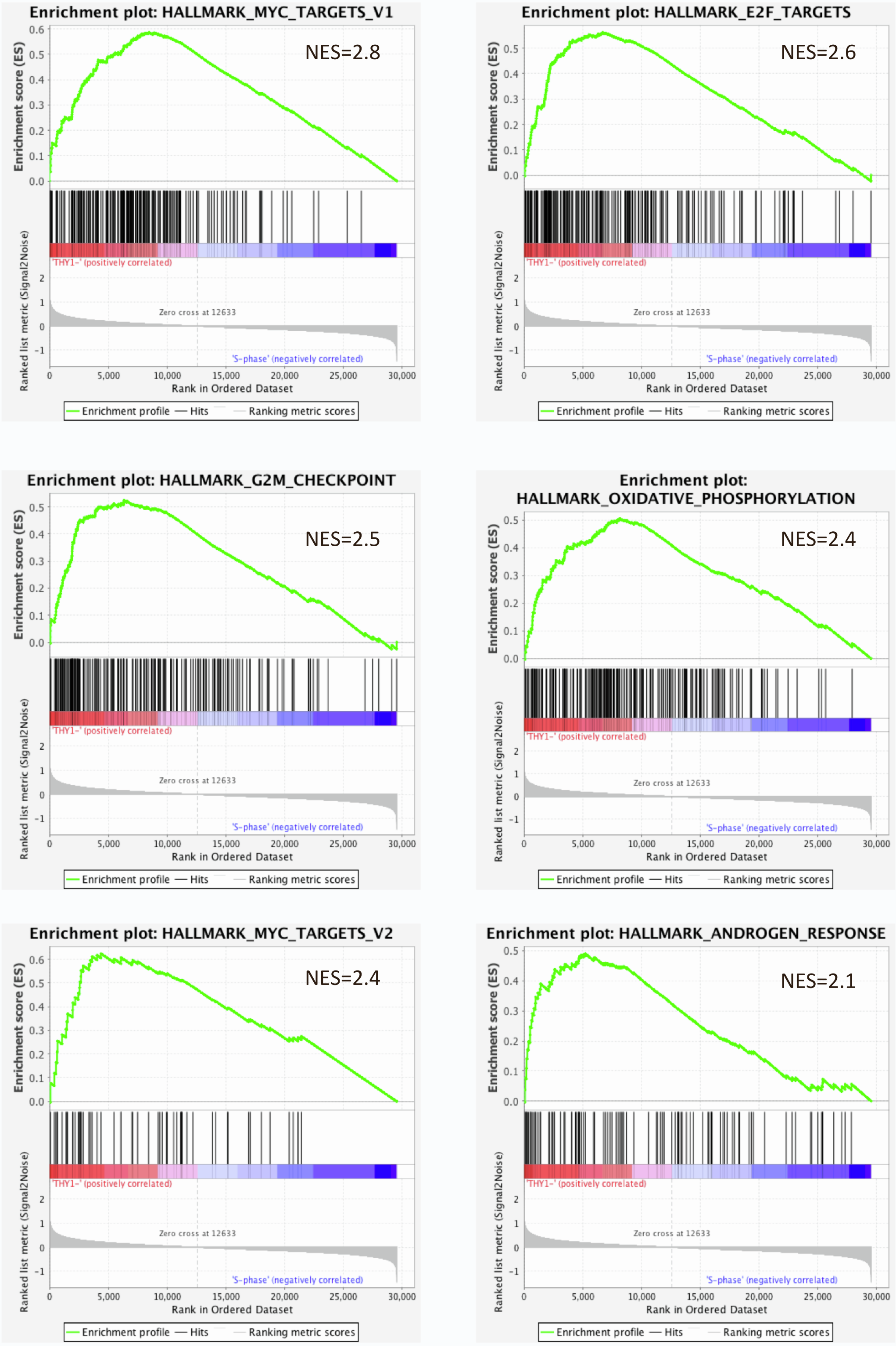

D

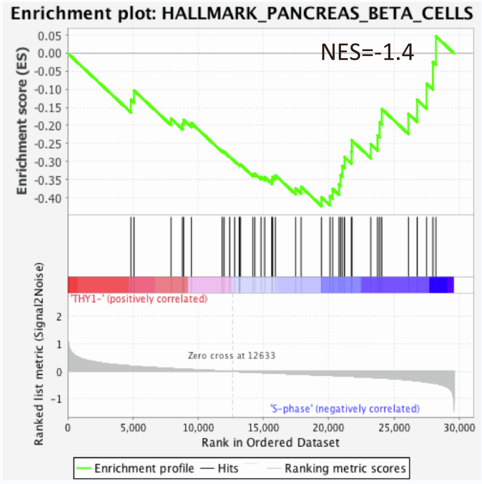

Figure S3

Supplemental Figure S3: Expression signature from comparison of β-2M$^-$ SPα-6^{med} THY1$^-$ with β-2M$^-$$\alpha$-6^{-med}THY1$^-$-"S-phase" spermatogonial cells

(A)Top-25 list of differentially expressed genes in β-2M$^-$ SPα-6$^+$THY1$^-$, (B) Top-25 list of differentially expressed genes in β-2M$^-$$\alpha$-6^{-med}THY1$^-$-"S-phase". (C and D) GSEA enrichment plots from GSEA hallmark analysis of pathways (C) in β-2M$^-$ SPα-6^{med}THY1$^-$ (FDR=0) and (D) in β-2M$^-$$\alpha$-6^{-med}THY1$^-$-"S-phase" (FDR $\leq$ 0.05).

A

Symbol	FC
FBXW10	88,29
LOC107986582	74,37
MCHR2-AS1	58,63
HHLA3-AS1	49,91
TMIGD3	47,68
PLAAT5	46,85
LINC02619	41,48
LINC00882	38,88
ROPN1L-AS1	38,36
AQP5	36,96
HSD52	36,45
LOC101928721	34,38
LINC01766	32,94
C9orf57	32,25
CABS1	32,12
LOC105375843	31,73
LINC00911	31,37
LOC100507071	31,36
LOC100240728	31,33
LOC105378044	30,23
ZNF385D	30,06
PPFIA2-AS1	29,30
ACR	29,17
TMEM225	28,70
ZC2HC1B	28,40

B

Symbol	FC
SSX3	56,14
H2AC20	50,20
ZNF280C	28,83
TEX19	25,77
STEAP1	25,36
FAM197Y9	25,22
H2AC8	24,89
HPRT1	24,26
H3C11	22,87
CT45A10	22,24
PAGE1	22,14
PRR20G	19,81
WDR44	19,21
CCNB3	19,17
ATP2B1	18,67
H13	18,63
CT55	17,53
PRDM7	17,36
CDC6	17,07
GBA	16,95
PRSS38	16,88
FBXO5	16,22
MAGEC1	16,05
ATP11C	15,88
BEND2	15,26

C

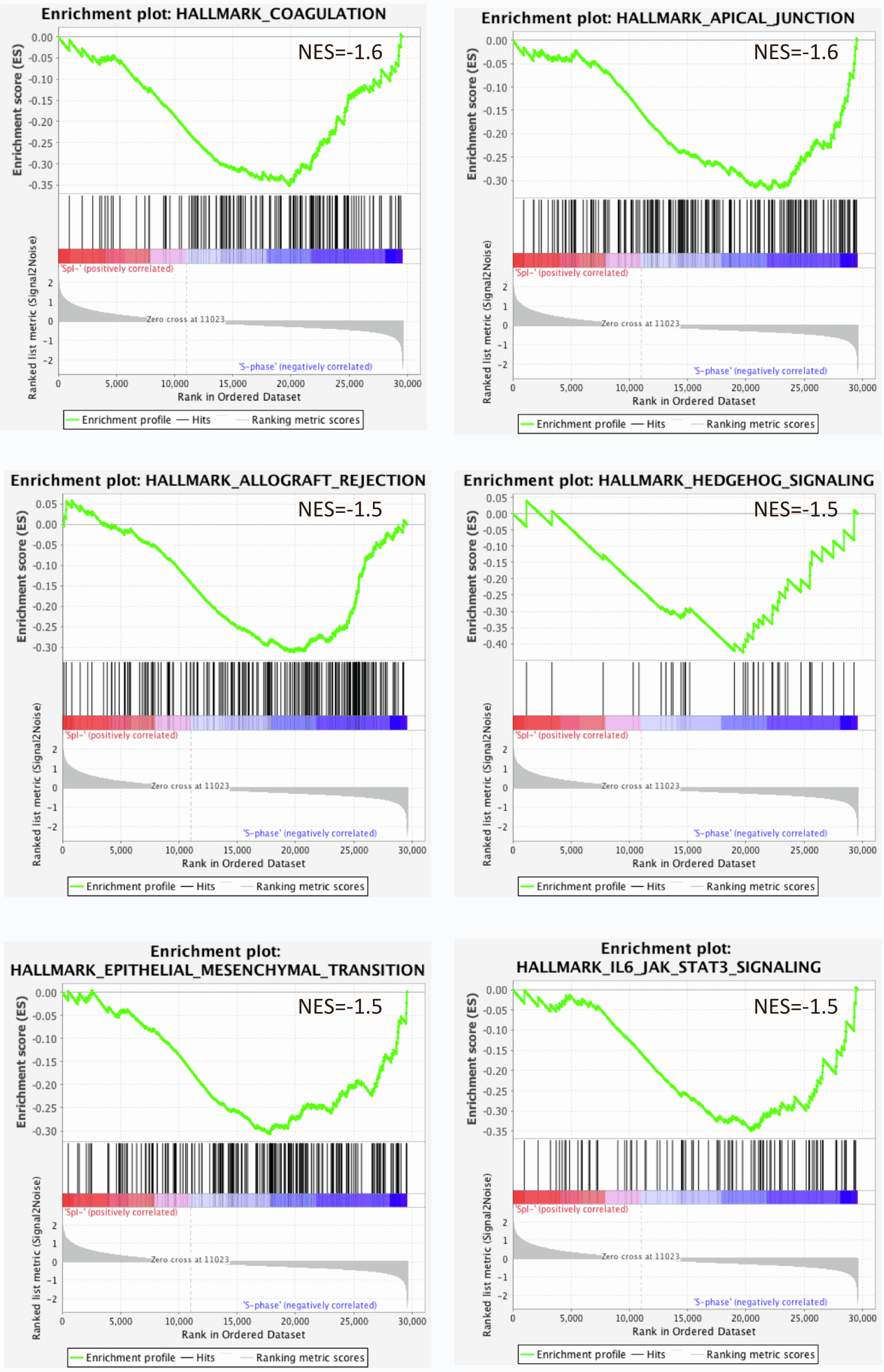

D

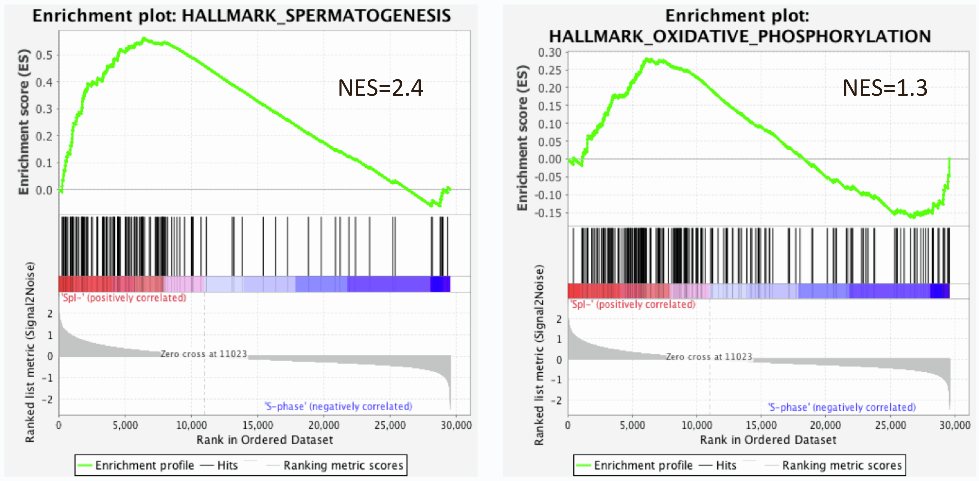

Figure S4

Supplemental Figure S4: Expression signature from comparison of β-2M$^{-}\alpha$-6^{-med}THY1^{-} -“S-phase” with spermatocyte I cells.

(A)Top-25 list of differentially expressed genes in β-2M$^{-}\alpha$-6^{-med}THY1^{-}-“S-phase”, (B) Top-25 list of differentially expressed genes in spermatocyte I. (C and D) GSEA enrichment plots from GSEA hallmark analysis of pathways (C) in β-2M$^{-}\alpha$-6^{-med}THY1^{-}-“S-phase” (FDR<0.1) and (D) in spermatocyte I (FDR <0.3).

A

Symbol	FC
H16	57,24
C9orf57	55,85
H34	53,14
H2AC1	40,28
GOLGA2P11	32,67
SELENOT	28,85
HLTF	23,08
MAD2L1	21,23
LOC100507384	20,86
H2BC1	20,15
C4orf46	19,95
TDRG1	19,85
LOC105370612	18,75
MARK2P9	18,17
NBPF1	18,00
LINC00865	17,61
LINC02721	17,25
LINC02475	17,22
LOC105369649	16,92
LOC105370613	16,10
IQCB1	15,89
H11	15,67
C18orf63	15,35
NAE1	14,54
TSNAX	14,16

B

Symbol	FC
LINC00524	24,56
LOC101927269	22,39
LINC00411	21,87
LOC105370739	21,45
LOC101928093	21,01
LINC01919	20,95
LOC107984561	19,96
LOC107985743	18,41
LOC105370248	17,99
CACNA1C-IT3	17,79
LOC105372038	17,18
LOC102724080	16,26
LOC105369714	15,89
LOC105371010	15,70
DIAPH1-AS1	14,47
LOC105374258	14,40
LOC105378885	14,39
LINC01717	14,11
LOC105375559	14,07
OXCT2	13,90
LOC105371371	13,80
HSFX4	13,74
LOC105375377	13,73
LOC105371342	13,71
LOC105372750	13,70

C

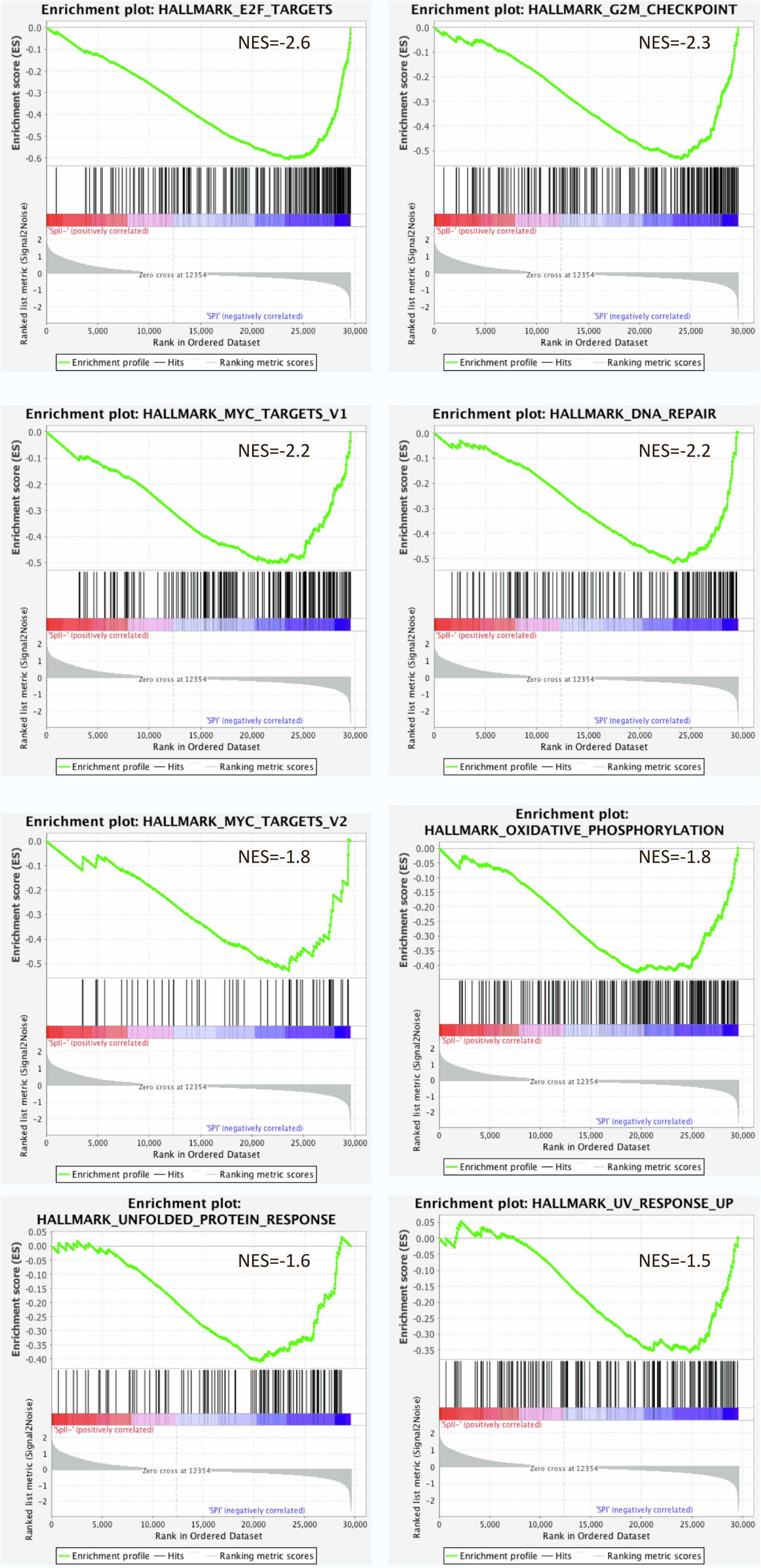

Figure S5

Supplemental Figure S5: Expression signature from comparison of spermatocyte I with spermatocyte II cells.

(A)Top-25 list of differentially expressed genes in spermatocyte I, (B) Top-25 list of differentially expressed genes in spermatocyte II (C) GSEA enrichment plots from GSEA hallmark analysis of pathways in spermatocyte I (FDR ≤ 0.005). No enrichment of pathways was found in spermatocyte II with FDR<1 and NES>1.3.

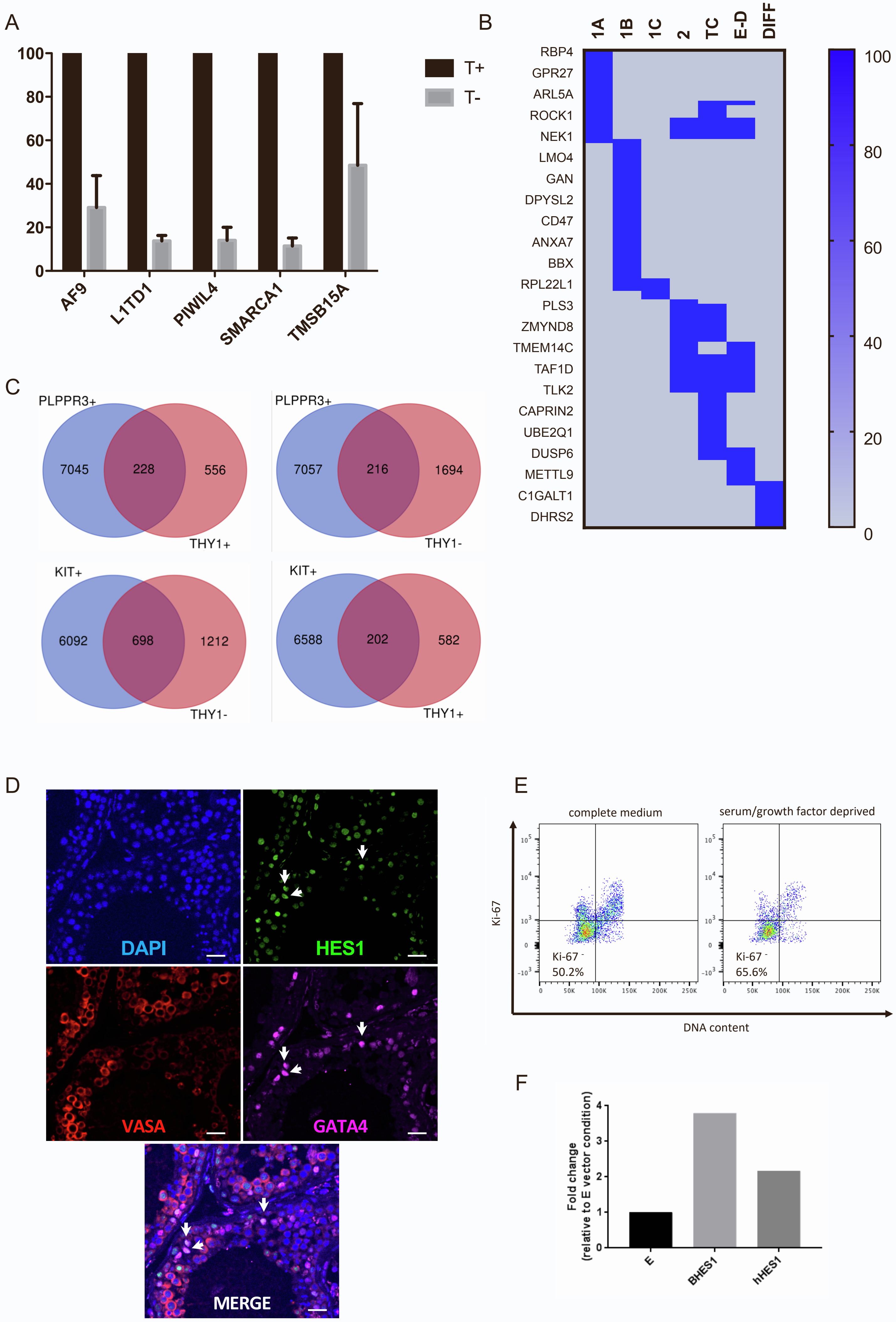
A
T+
T-
AF9
L1TD1
PIWIL4
SMARCA1
TMSB15A
B
1A
1B
1C
2
TC
E-D
DIFF
RBP4
GPR27
ARL5A
ROCK1
NEK1
LMO4
GAN
DPYSL2
CD47
ANXA7
BBX
RPL22L1
PLS3
ZMYND8
TMEM14C
TAF1D
TLK2
CAPRIN2
UBE2Q1
DUSP6
METTL9
C1GALT1
DHRS2
C
PLPPR3+
THY1+
7045
228
556
PLPPR3+
THY1-
7057
216
1694
KIT+
THY1-
6092
698
1212
KIT+
THY1+
6588
202
582
D
DAPI
HES1
VASA
GATA4
MERGE
E
complete medium
serum/growth factor deprived
Ki-67
Ki-67 - 50.2%
Ki-67 - 65.6%
DNA content
F
Fold change (relative to E vector condition)
E
BHES1
hHES1

Figure S6

Supplemental Figure S6: Analysis of genes enriched in the $\beta 2M^{-}SP\alpha\text{-}6^{+}THY1^{+}$ transcriptome

(A) Validation by qRT-PCR of genes found to be overexpressed in $\beta 2M^{-}SP\alpha\text{-}6^{+}THY1^{+}$ (T^{+}) compared to $\beta 2M^{-}SP\alpha\text{-}6^{med}THY1^{-}$ (T^{-}) on Affymetrix microarray. (B) Heatmap showing the distribution of the expression of genes in $\beta 2M^{-}SP\alpha\text{-}6^{+}THY1^{+}$ population according to the gene clusters defining the different states in the human adult SSC development as defined by Sohni et al., {Sohni et al., 2019, #56519}. Clusters SSC 1A, 1B, 1C and 2 describing stem cell population, transition cells (TC), Early differentiating spermatogonia (ED) and Differentiating spermatogonia (DIFF) (Sohni et al., 2019). (C) Venn diagram showing the relationships between the transcriptomes of human $\beta\text{-}2M^{-}SP\alpha\text{-}6^{+}THY1^{+}$, $\beta\text{-}2M^{-}SP\alpha\text{-}6^{med}THY1^{-}$, $PLPPR3^{+}$ (Tan et al., 2020) and KIT^{+} (Tan et al., 2020) spermatogonial populations. The number of genes in each group is indicated. (D) Immunofluorescent detection of HES1 and GATA-4 in human testes from obstructive azoospermia patient: DAPI (Blue), HES1 (green), VASA (red), GATA-4 (magenta), arrows (HES1-positive Sertoli cells), scale bar: 20 microns. (E) Ki-67/DNA content analysis of SSCs cultured in complete serum or under serum/growth factor-deprived conditions. (F) mRNA expression of *HES1* and *ΔBHES1* in SSCs transduced with *HES1* (HES1), *ΔBHES1* (BHES1), and *GFP* control (E) vectors.

Supplemental Table S1: List of DEGs enriched in the β–2M^{-}α-6^{med}THY1^{-}-“S-phase”, spermatocyte I (spI) and spermatocyte II (spII) populations at the spermatocyte I/β–2M^{-} α-6^{med}THY1^{-}-“S-phase” and spermatocyte II/spermatocyte I cell state transitions according to their chromosomal positions.

Supplemental Table S2: (A) List of DEGs in the β–2M^{-}SPα-6^{+}THY1^{+} (T^{+}), β-2M^{-}SPα-6^{med}THY1^{-} (T^{-}), spermatocyte I (4N), spermatocyte II (2N), and spermatid (N) populations at the different transitions of cell states (fc>2, p<0.02). (B) List of DEGs in the β–2M^{-}SPα-6^{+}THY1^{+} population (fc>2, p<0.02). (C) List of transcriptional regulators (TR) that varied across spermatogenesis and preferentially enriched in the β–2M^{-}SPα-6^{+}THY1^{+} population (fc>2, p<0.02).

GO cellular component complete	fold Enrichment	raw P-value	FDR
filopodium (GO:0030175)	4.45	4.86E-06	4.24E-04
lamellipodium (GO:0030027)	3.32	4.96E-06	4.14E-04
growth cone (GO:0030426)	2.84	2.20E-04	1.34E-02
site of polarized growth (GO:0030427)	2.73	3.45E-04	1.82E-02
RNA polymerase II transcription factor complex (GO:0090575)	2.73	7.56E-04	3.52E-02
nuclear transcription factor complex (GO:0044798)	2.51	8.26E-04	3.76E-02
actin-based cell projection (GO:0098858)	2.50	8.67E-04	3.86E-02
cell leading edge (GO:0031252)	2.45	6.41E-06	5.14E-04
secretory granule lumen (GO:0034774)	2.36	2.06E-04	1.29E-02
cytoplasmic vesicle lumen (GO:0060205)	2.33	2.39E-04	1.41E-02

Supplemental Table S3: Cellular component annotations of the β–2M^{-}SPα-6^{+}THY1^{+} gene list obtained according to the GO PANTHER classification

GO molecular function	fold Enrichment	raw P-value	FDR
protein binding involved in heterotypic cell-cell adhesion (GO:0086080)	14.51	1.62E-05	2.47E-03
Rac guanyl-nucleotide exchange factor activity (GO:0030676)	9.24	4.67E-04	4.91E-02
Rho guanyl-nucleotide exchange factor activity (GO:0005089)	5.59	2.04E-04	2.41E-02
transcription cofactor binding (GO:0001221)	5.13	3.41E-04	3.84E-02
cell-cell adhesion mediator activity (GO:0098632)	4.93	4.34E-04	4.66E-02
transmembrane receptor protein kinase activity (GO:0019199)	4.12	1.64E-04	2.05E-02
DNA-binding transcription repressor activity, RNA polymerase II-specific (GO:0001227)	3.12	1.79E-06	3.84E-04
DNA-binding transcription repressor activity (GO:0001217)	3.12	1.79E-06	3.67E-04
protein tyrosine kinase activity (GO:0004713)	3.03	4.22E-04	4.64E-02
phosphatase binding (GO:0019902)	2.84	2.20E-04	2.54E-02

Supplemental Table S4: Molecular function annotations of the β–2M$^-$SPα-6$^+$THY1$^+$ gene list obtained according to the GO PANTHER classification

NAME	NES	NOM p-val	FDR q-val
HALLMARK_UV_RESPONSE_DN	1.854	0.000	0.003
HALLMARK_INFLAMMATORY_RESPONSE	1.803	0.000	0.005
HALLMARK_COAGULATION	1.784	0.000	0.004
HALLMARK_ALLOGRAFT_REJECTION	1.693	0.000	0.007
HALLMARK_KRAS_SIGNALING_UP	1.689	0.000	0.006
HALLMARK_ANGIOGENESIS	1.647	0.007	0.008
HALLMARK_EPITHELIAL_MESENCHYMAL_TRANSITION	1.619	0.000	0.009
HALLMARK_TGF_BETA_SIGNALING	1.614	0.012	0.008
HALLMARK_HEDGEHOG_SIGNALING	1.543	0.019	0.015
HALLMARK_COMPLEMENT	1.485	0.005	0.024
HALLMARK_IL6_JAK_STAT3_SIGNALING	1.482	0.015	0.023
HALLMARK_APICAL_SURFACE	1.406	0.053	0.046
HALLMARK_INTERFERON_GAMMA_RESPONSE	1.402	0.008	0.044
HALLMARK_NOTCH_SIGNALING	1.388	0.077	0.046
HALLMARK_KRAS_SIGNALING_DN	1.374	0.018	0.049
HALLMARK_HYPOXIA	1.321	0.023	0.074

Supplemental Table S5: Pathways enriched in the β–2M^{-}SPα-6^{+}THY1^{+} population (GSEA analysis).

NAME	NES	NOM p-val	FDR q-val
HALLMARK_SPERMATOGENESIS	-2.862	0.000	0.000
HALLMARK_MYC_TARGETS_V1	-2.608	0.000	0.000
HALLMARK_E2F_TARGETS	-2.561	0.000	0.000
HALLMARK_OXIDATIVE_PHOSPHORYLATION	-2.251	0.000	0.000
HALLMARK_G2M_CHECKPOINT	-2.104	0.000	0.000
HALLMARK_DNA_REPAIR	-2.061	0.000	0.000
HALLMARK_MYC_TARGETS_V2	-1.999	0.000	0.000
HALLMARK_UV_RESPONSE_UP	-1.828	0.000	0.001
HALLMARK_UNFOLDED_PROTEIN_RESPONSE	-1.752	0.000	0.002

Supplemental Table S6: Pathways enriched in the β-2M$^-$ SPα-6^{med} THY1$^-$ population (GSEA analysis)

Supplemental Table S7: (A) List of DEGs from comparison between the undifferentiating spermatogonia (KIT-) and the differentiating spermatogonia (KIT+) populations in mice (fc>1.5, p<0.02). (B) List of genes conserved between the human β-2M^{-}SPα6^{+}THY1^{+} and murine β-2M^{-}SPα-6^{+}c-kit- cell populations (fc>1.5, p<0.02 for both lists).

gene	primer	5'-3'
AF9	F	CAACGTTACCGCCATTTG
	R	GTCTGGGATGGTGTGAAG
CXCL9	F	GCATCATCTTGCTGGTTCTGATTGG
	R	GCGACCCTTTCTCACTACTGGGGT
L1TD1	F	TCCCACAAAAGGAAGAAATAAATC
	R	GCTCTATGCTTTGAGTCTATTAGGG
PIWIL4	F	AATGCTCGCTTTGAACTAGAGAC
	R	ATTTTGGGGTAGTCCACATTAAATC
SMARCA1	F	ACGGCCTCCAAAACAGCCAAATG
	R	TGAGCCAGAGCTGGATTTGGGATA
TMSB15A	F	CCGCGAACAGCCTTTCAC
	R	CGACAAGTCTGGCTTATCACTCA
GAPDH	F	GTCGGAGTCAACGGATTTGG
	R	AGCAGCCCTGGTGACCAG
NANOS2	F	GGCTGGAGATGTTGAGAGCAA
	R	AAAGGAAATCCAGTGGCGC
GFRA1	F	GGGAGAAGCCCAACTGTTTG
	R	GACAGCTGCTGACAGACCTTGA
ABCG2	F	CAGGAGGCCTTGGGATACTT
	R	GCTATAGAGGCCTGGGGATT
ID4	F	GAGCCGCGCTGTCCAGGTGTG
	R	CTGCTCTTCCCCCTCCCTCTCTAGT
PLZF	F	AGCGGTTCCTGGATAGTTTGC
	R	TTCGAAAACTGTGCACCACACT
NANOS2	F	GTCTTCGCAGGCTCACCT
	R	GGCATTGAAAGGTGTCAGC
VASA	F	GAAGCTGATCGCATGTTGGATA
	R	TGCAGCCAACCTTTGAATTTC
RFX4	F	CCCGGTCCAAACTCGGAAC
	R	TGGCTCTTATTACAGTGTCCAGT
RFX2	F	CTATGGGATTCGTCTGAAGCC
	R	GGAGACATCTATGTACTGCTGGT
TNP2	F	CAGAGTTGCAGACAGAGCCAT
	R	TCATAGTCTTTTTGTGGCGCTT
CREM	F	ACACCACCTAGTATTGCTACCA
	R	GGATTGTTCCACCTTGGGCTAT
GUS	F	CCGAGTGAAGATCCCCTTTTTA
	R	CTCATTTGGAATTTTGCCGATT
HES1	F	CCAGCCAGTGTCAACACGA
	R	AATGCCGGGAGCTATCTTTCT

TSPAN33	F	CCGCTGGTGAAATACCTGCTC
	R	AGGGCTGCTTCTGCATGCTT
LPPR3[1]	F	CTTCTGCCCTGCTTCTACTTCG
	R	CATAGCACTGGAAGCCCACC
UTF1[1]	F	CGGCTCCCAGCGAACCAG
	R	GACGGGCTGAAGCGGAGC
FGFR3[1]	F	CCGAGCGGATGGACAAGAAG
	R	GACCAGGCTCCACTGCTGAT
VIM[1]	F	GGACCAGCTAACCAACGACAAAG
	R	CTCTCTCTGAAGCATCTCCTCCT
PIWIL4[1]	F	CATCAAGTTCTCCCGTGTGC
	R	GACACAGAAATGGCAAACCC
C19orf84[1]	F	AGATGGAACAACCAAAGGACG
	R	GTTCAGGAGCAAGGGTGGAG
KIT	F	GGAAGCCTCTTCCCAAGGAC
	R	GCTGGCCTCACTTTCAGGAT
DDX4/VASA[3]	F	AAGAGAGGCGGCTATCGAGATGGA
	R	CGTTCACTTCCACTGCCACTTCTG
NANOS3[2]	F	ACGCTTCTGCCCACTTAC
	R	TTCTTGCCTGCCGAGTTT
STRA8[4]	F	AATCCCATGACAGAGCAAC
	R	TTATCCAGGGTTTGCTCCAG

[1]From Sohni et al., 2019; [2]From Hermann et a, 2018, [3] From Anderson et al., Conserved and divergent patterns of expression of DAZL, VASA and OCT4 in the germ cells of the human fetal ovary and testis. *BMC Dev Biol.* **7**, 136 (2007),[4] From Medrano et al., Human somatic cells subjected to genetic induction with six germ line-related factors display meiotic germ cell-like features Scientific report, 6, 24956 (2016).

Supplemental Table S8: Primer list

Supplemental Experimental procedures

Experimental model and human materials

Adult human testis biopsies from obstructive azoospermia patients with normal spermatogenesis were obtained from the CECOS Hospital Cochin. All patients consented to inclusion in this research study (IRB-approved protocol: IRB 00003835; 2012/40ICB; French Institutional Review Board-Comité de Protection des Personnes, Ile de France IV). The C57Bl6/J, FVB/N, and immunodeficient NOD/Shi-scid/IL-2Rγ^{null} (NSG) mice were housed in our animal facility. All experiments were performed in compliance with European legislation and the guidelines of the Ethics Committee of the French Ministry of Agriculture (Agreement B9203202). All animal-related procedures were performed in compliance with the European Communities Council Directive of 22th September 2010 (EC/2010/63) and were approved by Comité d'Ethique en Expérimentation Animale, Direction de la Recherche Fondamentale, CEA (authorization 14-081; CEtEA-CEA DRF IdF).

Testicular single-cell suspensions, immunomagnetic and flow cell sorting, and flow cytometry analysis

Testicular single-cell suspensions were prepared from human biopsies. The tissue was incubated in trypsin 0.25% containing collagenase I (final concentration, 0.5 mg/ml) for 20 minutes at 34°C. The cell suspension was then filtered (20 μm). The vital DNA dye Vybrant, which was previously shown to identify cells with the SP phenotype during hematopoiesis, was used instead of Hoechst 33342, owing to the wavelength

excitation maxima (369 nm) of the Vybrant fluorophore (Telford et al., 2007). Vybrant staining (1 µg/ml) of the cell suspensions was performed as previously described (Barroca et al., 2009; Corbineau et al., 2017), and cells were labeled with anti-α6 integrin-PE (GoH3) (BD Pharmingen), β2M-FITC (BD Pharmingen) and anti-THY1-APC (BD Pharmingen) antibodies. Propidium iodide (Sigma) was added before cell sorting to exclude dead cells. Vybrant efflux inhibition was performed by pre-incubating human testicular cells (10^6 cells/ml) for 30 minutes at 32°C in incubation medium supplemented with the specific BCRP1 inhibitor Ko143 (200 nM). The effect of the Ko143 on the SP phenotype was estimated using the formula (% SP^{Ctrl} - % SP^{Ko143} /%SP^{Ctrl}) X100.

Murine testicular single-cell suspensions were prepared from 2- to 3-month-old FVB/N mice as previously described (Barroca et al., 2009; Corbineau et al., 2017). The immunomagnetic selection of $\alpha6^{+}$ cells was performed using anti-α6 integrin-PE (GoH3) (BD Pharmingen) and anti-PE (Miltenyi Biotec) microbeads according to the manufacturers' protocols. Hoechst staining (5 µg/ml) of the cell suspensions was performed. The cells were then labeled with β2m-FITC (Santa Cruz) and anti-CD117-APC (2B8) antibodies (BD Pharmingen). Propidium iodide (Sigma) was added before cell sorting to exclude dead cells (Barroca et al., 2009; Corbineau et al., 2017). Single-cell suspensions were analyzed using a FACSCalibur™ or LSRII flow cytometer system (BD Biosciences). BD Trucount™ Tubes (BD 340334) were used to assess the number of germinal cells and dead cells using propidium iodide and flow cytometry in SSC cultures. The data were analyzed with DIVA or FlowJo software. Cell sorting was performed using a FACSAria cytometer (BD Biosciences).

RNA extraction and quantitative RT-PCR

mRNA was prepared using RNeasy® Micro and Mini kits (Qiagen). The mRNA was then reverse-transcribed with a Quantitect kit (Qiagen). Quantitative RT-PCR was performed using an AB7900 device (Applied Biosystems) with Fast SYBR® Green Master Mix (Applied Biosystems). The primers are listed in Table S8.

SSC and MEF cultures

Murine adult SSC lines were obtained from C57Bl6/J and EGFP mice (Okabe et al., 1997) and maintained on mitomycin C-treated mouse embryonic fibroblasts (MEFs) as previously described (Barroca et al., 2009; Corbineau et al., 2017). The SSC culture medium was composed of Stem Span (Stemcell Technologies) and B27 supplement (Life Technologies) and supplemented with recombinant human GDNF (40 ng·ml^{-1}, R&D Systems), recombinant rat GFRA1 (300 ng·ml^{-1}, R&D Systems), FGF2 (1 ng·ml^{-1}, Life Technologies), and ES-CultTM Fetal Bovine Serum (1%, Stemcell Technologies). Every 3-4 days, the SSC clusters were split *via* enzymatic digestion with 0.05% trypsin-EDTA (Life Technologies). MEF cultures were established *via* trypsin digestion of 13.5 days post-coital (dpc) embryos, and the resulting cells were cultured in DMEM supplemented with 10% FBS, L-glutamine and penicillin/streptomycin. Human β-2M$^-$SP$^+$$\alpha$-6$^+$Thy-1$^+$ SSCs were maintained on gelatin-coated plates in SSC culture medium.

Hes1 expression was knocked down using siRNAs (5′-CGACACCGGACAAACCAAA-3′). SSCs culture were dissociated and 1 × 10^5 cells were transfected with *Hes1* siRNAs or negative control siRNAs by electroporation at 1400 V for 20 ms (2 pulses) using a Neon Transfection System and the Neon® Transfection System 10 µL Kit

(Thermo Fisher Scientific) according to the manufacturer's instructions. Transfected cells were then plated on MEFS in 96-well plates.

Lentiviral vector production and transduction of cells in SSC cultures

Viral particles were produced *via* transient transfection of 293T cells with the following lentiviral vectors: wt HES1, ∆BHES1 (gift from L. Chen, obtained from Addgene) (Yu et al., 2006), and pTRIP-GFP. The packaging plasmids were pCMVDR-8.92 and pMD2G. Viral supernatants were collected 48–72 h later. SSCs were infected by exposing the SSCs to viral supernatants overnight in SSC medium containing 5 μg ml^{-1} polybrene. The SSCs were then plated on mitomycin C-treated MEFs and expanded after selection *via* cell sorting based on GFP fluorescence.

Human and Mouse Testis Cell Transplantation

NSG and C57BL6J mice were used as the recipients for human and mouse cell transplantation, respectively. To deplete endogenous spermatogenesis, the recipient mice were treated at 6-8 weeks of age with busulfan (40 mg/kg at least 4 weeks before donor cell transplantation). Human or murine donor cells were resuspended in DMEM supplemented with 10% heat-inactivated fetal calf serum, 100 mg/ml DNase I (DN25, Sigma-Aldrich) and 4% trypan blue solution (T8154, Sigma-Aldrich) for transplantation as previously described (Barroca et al., 2009). A 10-microliter solution of donor cells was introduced into the seminiferous tubules of the testis of the recipient mouse *via* an injection through the efferent ductules as previously described. Ten weeks after transplantation, the recipient testis were collected and analyzed by either macroscopic observation of fluorescence in mice or immunohistochemistry in human tissues as described below. The capacity of donor-cells to home, proliferate and give rise to high

number of spermatogonial progeny in the recipient testis is linked to the stem cell potential. The colonies containing the higher number of spermatogonial progeny must originate from the most primitive spermatogonia, with the highest stem cell potential. For human, two categories of cell clusters were arbitrarily defined, one likely derived from donor-cells with a lower regenerative potential (4-8 cell clusters) and one derived from donor-cells with higher regenerative potential (>8 cells). Macroscopic observations of recipient testis tissues were performed using an Olympus epifluorescence microscope to detect the presence of EGFP-fluorescent seminiferous tubules.

Histology and immunofluorescence

In human cell analyses, the recipient testis were fixed in 4% paraformaldehyde, frozen in Tissue-Freezing medium (OCT, Sakura, Netherlands), and sectioned at a thickness of 10 microns. The sections were stained overnight at 4°C with primary antibodies (see below) against human nuclei (MAB 1281, Millipore), MAGEA4 (HPA021942, Sigma-Aldrich), PLZF (sc22839, Santa-Cruz Biotech), VASA (ab13840, Abcam), or HES1 (ab119776, Abcam). Then, the sections were incubated for 2 h at room temperature with the corresponding secondary antibodies (Life-Technology, OR, USA), donkey anti-mouse Alexa-fluor 488 and donkey anti-rabbit Alexa-fluor 594, respectively. Cell nuclei were counterstained with 4',6-diamidino-2-phenylindole (DAPI). VASA/β2M and HES1/GATA4/VASA expression analysis were performed on formalin-fixed paraffin-embedded sections of human testis with primary antibodies against β2M (ab175031, Abcam), VASA (ab13840, Abcam), HES1 (ab196328, Abcam) and GATA4 (sc1237, Santa Cruz Biotech), and then with the corresponding secondary antibodies. Imaging

was performed using an Olympus AX70 epifluorescence microscope equipped with a CoolSNAP Myo camera (Photometrics) in Micro-Manager software (version 1.4.16, open-source microscopy) or a Nikon A1 laser fluorescence confocal microscope with NIS Elements software (version 4.51, Nikon).

Transcriptome

From several donors, three cell samples were sorted for each differentiation stage, in order to prepare RNA to be subsequently hybridized to Affymetrix high-density oligonucleotide chips. Germinal populations were directly sorted *via* flow cytometry in lysis buffer obtained from an RNeasy Micro kit (Qiagen). The quantity and quality of the RNA were analyzed using a 21000 Bioanalyzer (Agilent Technologies). Only samples with an RNA integrity number (RIN) equal to or above 7 were used for the transcriptomic analyses. RNA samples were then analyzed using an Affymetrix® Human Gene 2.1 ST Array and an Affymetrix GeneChip™ Mouse gene 2.0 ST Array (Thermo Fisher Scientific). Data were robust multi-array average normalized with R/Bioconductor, and ANOVA test was applied to extract DEGs. Data were analyzed using Panther (http://www.pantherdb.org) (Mi et al., 2019), Ingenuity Pathway Analysis, and String (http://string-db.org) (Szklarczyk et al., 2019) were used. Gene set enrichment analysis (GSEA) was also performed as described previously (Subramanian et al., 2005). GEO accession number for microarray data: GSE155509

Additional References

Mi, H., Muruganujan, A., Ebert, D., Huang, X., and Thomas, P.D. (2019) PANTHER version 14: more genomes, a new PANTHER GO-slim and improvements in enrichment analysis tools. Nucleic Acids Res, *47*, D419–D426.

Okabe, M., Ikawa, M., Kominami, K., Nakanishi, T., and Nishimune, Y. (1997) Green mice' as a source of ubiquitous green cells. FEBS Lett, *407*, 313–319.

Subramanian, A., Tamayo, P., Mootha, V.K., Mukherjee, S., Ebert, B.L., Gillette, M.A., Paulovich, A., Pomeroy, S.L., Golub, T.R., Lander, E.S. et al. (2005) Gene set enrichment analysis: a knowledge-based approach for interpreting genome-wide expression profiles. Proc Natl Acad Sci U S A, *102*, 15545–15550.

Szklarczyk, D., Gable, A.L., Lyon, D., Junge, A., Wyder, S., Huerta-Cepas, J., Simonovic, M., Doncheva, N.T., Morris, J.H., Bork, P. et al. (2019) STRING v11: protein-protein association networks with increased coverage, supporting functional discovery in genome-wide experimental datasets. Nucleic Acids Res, *47*, D607–D613.

Telford, W.G., Bradford, J., Godfrey, W., Robey, R.W., and Bates, S.E. (2007) Side population analysis using a violet-excited cell-permeable DNA binding dye. Stem Cells, *25*, 1029–1036.
